# Recurrence affects the geometry of visual representations across the ventral visual stream in the human brain

**DOI:** 10.1371/journal.pbio.3003354

**Published:** 2025-08-25

**Authors:** Siying Xie, Johannes Singer, Bati Yilmaz, Daniel Kaiser, Radoslaw M. Cichy

**Affiliations:** 1 Department of Education and Psychology, Freie Universität Berlin, Berlin, Germany; 2 Department of Mathematics and Computer Science, Physics, Geography, Mathematical Institute, Justus Liebig University Gießen, Gießen, Germany; 3 Center for Mind, Brain and Behavior (CMBB), Philipps-University Marburg, Justus Liebig University Gießen and Technical University Darmstadt, Giessen, Germany; 4 Cluster of Excellence “The Adaptive Mind”, Philipps-University Marburg, Justus Liebig University Gießen and Technical University Darmstadt, Giessen, Germany; 5 Berlin School of Mind and Brain, Faculty of Philosophy, Humboldt-Universität zu Berlin, Berlin, Germany; 6 Bernstein Center for Computational Neuroscience Berlin, Berlin, Germany; McGill University, CANADA

## Abstract

The human brain orchestrates object vision through an interplay of feedforward processing in concert with recurrent processing. However, where, when, and how recurrent processing contributes to visual processing is incompletely understood due to the difficulties in teasing apart feedforward and recurrent processing. We combined a backward masking paradigm with multivariate analysis on EEG and fMRI data to isolate and characterize the nature of recurrent processing. We find that recurrent processing substantially shapes visual representations across the ventral visual stream, starting early on at around 100 ms in early visual cortex (EVC) and two later phases of around 175 and 300 ms in lateral occipital cortex (LOC), adding persistent rather than transient neural dynamics to visual processing. Using convolutional neural network models for comparison with the brain, we show that recurrence changes the feature format in LOC from predominantly mid-level to more high-level features. Finally, we show that recurrence is mediated by four distinct spectro-temporal neural components, which span the theta to beta frequency range. Together, our results reveal the nature and mechanisms of the effects of recurrent processing on the visual representations in the human brain.

## Abstract 2

The specific roles of feedforward and recurrent processing in human visual object recognition remain incompletely understood. In this neuroimaging and computational modelling study the authors isolate and characterize the nature of recurrent processing, providing deeper insights into when, where, and how it affects object representations.

## Introduction

Human visual object recognition is orchestrated by the interplay of feedforward and recurrent computations. Anatomically, this is mediated by feedforward as well as recurrent connections. Here, we define recurrence broadly, including lateral connections within a cortical region, as well as short- and long-range, cortico-cortical and subcortical-cortical feedback [1–3]. Feedforward sweep brings in information from the retina, enabling core object recognition through basic visual analysis [4,5]. Then, recurrent computations begin right after the first influx of feedforward information into the cortex [6–8]. Recurrent activity contributes to object recognition not only when the viewing conditions are challenging [9–15], but also when objects are in plain view [16–18].

While the existence and importance of both feedforward and recurrent computations in object recognition is undoubted, their exact nature, i.e., where, when, and how they affect visual processing remains incompletely understood [19–22]. This is partly because their empirical dissection is challenging: shortly after the first feedforward sweep, feedforward and recurrent activity overlap in space and time [23–25], hindering their unique characterization.

Here, we used the classical experimental protocol of backward masking [26–29] to isolate the role of recurrent from feedforward activity [30–34]. In backward masking a salient visual mask is shown shortly after a target image, impacting recurrent activity related to the target while leaving feedforward activity unaffected [31,35–37]. Backward masking alters early and late recurrent responses within individual brain regions [10,38], as well as disrupts recurrent connections between neighboring [31,33] and distant [39,40] brain regions. Thus, the comparison of brain activity when participants view masked versus unmasked target images isolates the contribution of recurrent activity.

We recorded human brain activity with EEG and fMRI to resolve visual responses in time and space when a set of naturalistic object stimuli were either backward masked or not using a sequence of synthesized texture images. Participants performed a 2-alternative forced-choice (2-AFC) task on 20% of EEG trials and identified immediate repetitions in a one-back task on 10% of fMRI trials. We then applied multivariate pattern analysis [41–43] to recover the neural representations of object image content across the different masking regimes, over time and space.

Comparing the neural activity related to the target images in the masked and unmasked conditions, we determined where, when, and how recurrent activity contributes to human object vision. We first characterized the spatiotemporal dynamics of visual recurrent activity, then examined its spectral basis using time-frequency decomposition [44–47]. Finally, we assessed how recurrence shapes visual representations by comparing neural data to those from a feedforward convolutional neural network (CNN) [48–50]. It is important to note that we did not use the CNN to capture brain activity and its recurrent dynamics. The CNN rather serves as a reference model for inferring representational formats in visual cortex, where the model’s well-characterized hierarchical structure allows us to gauge the relative feature complexity within neural representations.

## Results

**Fig 1:**
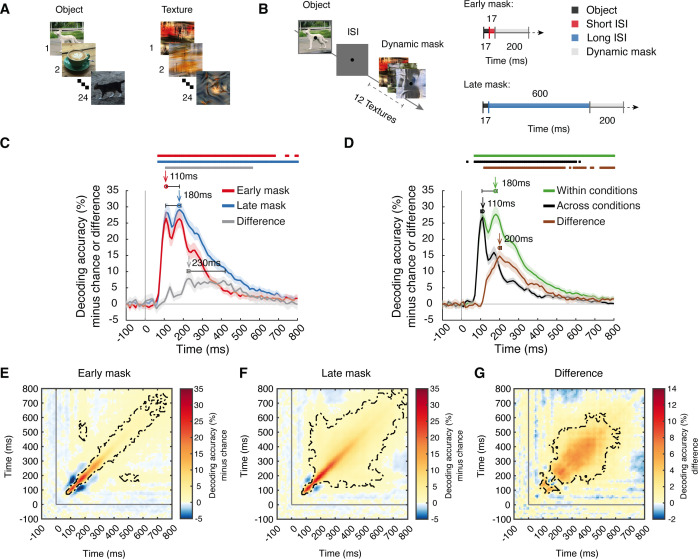
Experimental design and temporal dynamics of visual object representations. **(A)** Stimulus set. We used 24 real-world object images on natural backgrounds as target stimuli and 24 synthesized image textures created from an additional set of real-world object images for dynamic masks. Images shown in the figure are self-taken photographs used as substitutes to comply with copyright requirements. **(B)** Experimental paradigm and timing parameters. On each trial, a briefly shown target object image was backward masked by a dynamic mask (i.e., a sequence of image textures) in one of two conditions: the early mask condition (short 17 ms ISI) and the late mask condition (long 600 ms ISI). **(C)** Results of object identity decoding in the early mask (red) and late mask (blue) conditions and their difference (gray). **(D)** Results of object identity decoding within (green, corresponds to the average of red and blue in (C)) and across (black) masking conditions and their difference (brown). For **(C, D)**, chance level is 50%; significant above-chance level decoding is denoted by colored asterisks at the corresponding time points (*N* = 31, *p* < 0.05, right-tailed permutation tests, cluster definition threshold *p* < 0.005, cluster-threshold *p* < 0.05, 10,000 permutations); vertical gray line at 0 ms indicates stimulus onset; shaded margins of time courses indicate 95% confidence intervals of the decoding performance determined by bootstrapping (1,000 iterations); horizontal error bars indicate 95% confidence intervals for peak latencies. **(E–G)** Results of time-generalized decoding object identity in the **(E)** early mask condition, **(F)** late mask condition, and **(G)** the difference. For **(E–G)**, chance level is 50%; time point combinations with significantly above-chance level decoding are outlined in black dashed lines (*N* = 31, right-tailed permutation tests, cluster definition threshold *p* < 0.005, cluster-threshold *p* < 0.05, 10,000 permutations); vertical and horizontal gray lines indicate stimulus onset. The data underlying this figure can be found at https://osf.io/U3VG9/.

We presented 24 images of everyday objects on real-world backgrounds (Fig 1A) to human participants while recording their brain activity with EEG (*N* = 31) and fMRI (*N* = 27) in separate sessions. On each trial, the target image was backward masked in one of two masking conditions: early mask or late mask (Fig 1B). In the early mask condition, a dynamic mask rapidly followed the target after 17 ms. The rapid succession of target and mask yields effective backward masking that disrupts recurrent processing [31,35–37]. In contrast, in the late mask condition, the mask appeared after a delay of 600 ms, leaving recurrent processing unaffected across an extended time window while otherwise keeping the stimulation across the whole trial the same. Participants had high task performance in both masking conditions across both EEG and fMRI sessions, with high correctness and d-prime scores overall (EEG correctness minus chance (50%): early mask = 29.03 ± 8.67%, late mask = 37.51 ± 6.11%; fMRI d-prime: early mask = 2.01 ± 1.01, late mask = 4.53 ± 2.41), and as expected, the task performance was worse for the early mask than the late mask condition (EEG: *p* < 0.001; fMRI: *p* < 0.001; see S8 Table for full behavioral results).

We used a multivariate pattern analysis framework to assess visual object representations captured by EEG and fMRI [42,51] to classify the objects in the target images from brain data. Across trials, the masks were randomly paired with the stimuli. Thus, the successful classification of target images is based on the neural activity elicited by the target image only, even if it overlaps with neural activity elicited by the mask, as the mask content was independent (and thus not predictive) of the target image.

We then characterized and compared object representations across the early mask and late mask conditions, revealing the temporal, spatial, and spectral characteristics as well as the representational format of the recurrent aspects of visual processing.

### The temporal dynamics of recurrent visual activity

To reveal the temporal dynamics of object representations in the early mask and late mask conditions, we conducted time-resolved multivariate pattern classification of object identity using EEG data. Classifying between all pairs of the 24 object images and averaging across pairs yielded a grand average object decoding time course for both masking conditions (Fig 1C, for statistical details, see S1 Table). We assessed statistical significance using cluster-based inference (*N* = 31, right-tailed permutation tests, cluster definition threshold *p* < 0.005, cluster-threshold *p* < 0.05, 10,000 permutations), and report peak latencies as time points at which objects are best discriminated by neural representations with 95% confidence intervals derived by bootstrapping (1,000 samples) in brackets.

We observed a qualitatively similar and typical results pattern [51,52] in both masking conditions. Decoding accuracies fluctuated around baseline until 70 ms after image onset, when they steeply rose to two peaks at ~100 ms and ~200 ms. The peak latencies for the objects in the early mask condition (110 ms [110–180 ms]) and the late mask condition (180 ms [110–190 ms]) coincided with the first and second peak, respectively, without being significantly different (*p* > 0.05, S1 Table). This result demonstrates the presence of robust visual information in both masking conditions, warranting further analysis.

Comparing the decoding performance between the two masking conditions, we observed higher decoding in the late mask condition emerging after the first decoding peak (Fig 1C, gray curve, cluster 110–560 ms, peak latency 230 ms [220–420 ms]). This pattern was also present when decoding objects across the categorical boundary defined by naturalness or animacy (S1A, S1B Fig, and S2 Table). Together, this provides a first characterization of the timing of recurrent activity.

The modest difference in the time-resolved decoding result patterns between the early and the late mask conditions might be interpreted as indicating a relatively minor role of recurrent processing in visual object processing. However, this conclusion is premature: similar overall time courses might hide qualitatively different visual representations across the two masking conditions.

To investigate whether the representations are strongly affected by recurrent processing, we decoded object identity across the two masking conditions [53,54]. The rationale is that if visual representations are only weakly affected by recurrent processing, decoding results should be similar for the decoding within- and across-masking conditions. However, if recurrent processing affects visual representations more strongly, the across-condition decoding accuracy should be lower than the within-condition accuracy. We found that cross-decoding was strongly reduced after the first peak (110 ms [100–110 ms], Fig 1D, black curve) when compared to decoding within each masking condition (Fig 1D, green curve, corresponds to the average of the blue and red curves in Fig 1C). The difference between within- and across-conditions was significant after the first within-condition decoding peak (Fig 1D, brown curve, clusters between 120 ms and 800 ms), with a peak at 200 ms (200–210 ms) (for statistical details, see S1 Table). This result pattern was also obtained when comparing within- and across-conditions decoding for training the classifiers on either the early or the late mask condition (S1C, S1D Fig, and S2 Table). This indicates that recurrent processing strongly affects visual object representations after the first feedforward sweep from 120 ms onward, thus detailing the temporal dynamics of recurrent processing.

If recurrent processing strongly affects visual object representations, the dynamics with which those representations emerge should also differ depending on the amount of recurrent activity involved. To assess this, we used time-generalization analysis (TGA) [55] by decoding object identity across all time point combinations in the EEG epoch. This resulted in time-time matrices for each masking condition (Fig 1E and 1F) and their difference (Fig 1G).

We observed similarities and differences between the two masking conditions. Concerning the similarities, in both masking conditions, significant effects were present from ~70 ms onwards, and decoding accuracies were highest close to the diagonal (i.e., similar time points for training and testing), indicating that fast-evolving, transient representations dominate the neural dynamics. Further, we also observed significant off-diagonal generalization from 150 ms on in both masking conditions, indicating the additional presence of stable and persistent representations. This shows that in both masking conditions, visual processing depends on both transient and persistent representations.

However, we also observed two key differences. For one, there was more widespread temporal generalization in the late mask than in the early mask condition (Fig 1E and 1F), and this difference was significant (Fig 1G). This suggests a stronger presence of persistent representations due to recurrent processing in the late mask condition. Second, we observed that below-chance decoding accuracies at the time point combinations, i.e., ~ 100 ms and ~200 ms (Fig 1E–1G ), were lower in the early mask condition than in the late mask condition, emerging as a positive difference in their comparison (Fig 1G).

How are the negative decoding accuracies in off-diagonal regions of the Time-generalization analysis (TGA) to be explained? While commonly observed [51,55], the underlying neural dynamics are unknown. One tentative idea is that they might reflect systematic, stimulus-locked shifts in oscillatory phase patterns between the two time points at which the negative decoding occurs [56,57] (for graphical illustration, see S2 Fig). Assuming that in the early mask condition recurrent processing is reduced while feedforward processing is unaffected. This links feedforward activity to time-locked oscillatory components that are covered by time-varying recurrent activity in the late mask condition. In turn, in the early mask condition recurrent activity is reduced, and the time-locked feedforward-related oscillatory activity is uncovered. This result pattern was confirmed when comparing the decoding between within-condition of the late mask and the cross-decoding (S3A–S3C Fig), and it was reversed when comparing the decoding between the within-condition of the early mask and the cross-decoding (S3D–S3F Fig), supporting our interpretation.

Together, our results provide two key insights into the temporal dynamics of recurrent visual processing: first, recurrent processing affects visual object representations from ~100 ms onward, after the first feedforward sweep, and most strongly around 200 ms; secondly, it contributes specifically to the emergence of persistent representations.

### The spatial profile of recurrent visual activity

Next, we determined the spatial profile of recurrent processing across the visual brain. For this, we used an equivalent multivariate pattern analysis scheme and comparison strategy between masking conditions as for the temporal dynamics, but applied in a spatially resolved way to fMRI data.

**Fig 2:**
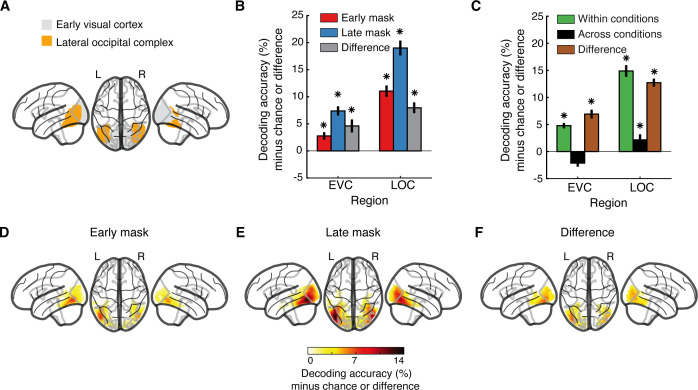
Cortical locus of visual object representations. **(A)** Visualization of the early visual cortex (i.e., V1, V2, and V3 combined) and the lateral occipital complex regions of interest. **(B)** Results of object identity decoding in the early mask condition (red), the late mask condition (blue), and their difference (gray). **(C)** Results of object identity decoding within (green, corresponds to the average of red and blue in **(B)**) and across (black) masking conditions and their difference (brown). For **(B, C)**, chance level is 50%; significant above-chance level decoding is denoted by black asterisks above the bars (*N* = 27, *p* < 0.05, right-tailed permutation tests, FDR-corrected); error bars indicate standard errors of the mean. **(D–F)** Results of the searchlight decoding in the **(D)** early mask condition, **(E)** late mask condition, and **(F)** the difference. For **(D–F)**, chance level is 50%; only voxels with significant above-chance level decoding are shown (*N* = 27, right-tailed permutation tests, cluster definition threshold *p* < 0.005, cluster-threshold *p* < 0.05). The data underlying this figure can be found at https://osf.io/U3VG9/.

We focused on two regions of interest (ROI) in the visual ventral stream: the early visual cortex (EVC) (i.e., V1, V2, and V3 combined) as the entry point of visual information in the cortex [58,59] and the lateral occipital complex (LOC) (Fig 2A) as a central high-level hub for object representations [60–62]. We decoded object identity in both masking conditions (Fig 2B) as well as across masking conditions (Fig 2C) and compared the results (*N* = 27, sign-permutation tests, FDR-corrected, *p* < 0.05).

In line with the EEG results, there was above-chance decoding of object identity in both ROIs in both masking conditions (Fig 2B, blue and red bars, all ROI-results FDR-corrected). Further comparing masking conditions, we found higher decoding accuracies for the late mask condition in EVC and LOC (Fig 2B, gray bars), indicating that recurrent processing affects representations in both regions.

Akin to the EEG analysis, we next determined the degree to which recurrent activity alters visual representations. For this, we compared the within-condition decoding results (Fig 2C, green bars, corresponds to the average of red and blue bars in Fig 2B) to the across-conditions results (Fig 2C, black bars), noting their difference (Fig 2C, brown bars). In both ROIs, the decoding accuracy was strongly reduced when decoding across masking conditions. In LOC, but not EVC, there was low but significant cross-decoding accuracy. An equivalent results pattern emerged when comparing within- and across-conditions decoding for training the classifiers on either the early or the late mask condition (S4A and S4B Fig). This indicates that recurrent activity strongly impacts visual representations in both EVC and LOC.

To explore the differences between the two masking conditions across the whole brain, we used an fMRI searchlight analysis [63,64]. Consistent with the ROI results, we found object identity information across the ventral visual stream in both masking conditions (Fig 2D and 2E, right-tailed permutation tests, cluster definition threshold *p* < 0.005, cluster-threshold *p* < 0.05, 5,000 permutations, for statistical details regarding peak locations and spatial extent, see S3 Table). Comparing decoding in the early mask versus the late mask conditions revealed widespread effects in the ventral stream with a maximum in the high-level ventral cortex (Fig 2F). This reinforces the view that recurrent activity strongly affects visual representations across the ventral stream.

### Recurrent processing affects the format of visual representations

**Fig 3:**
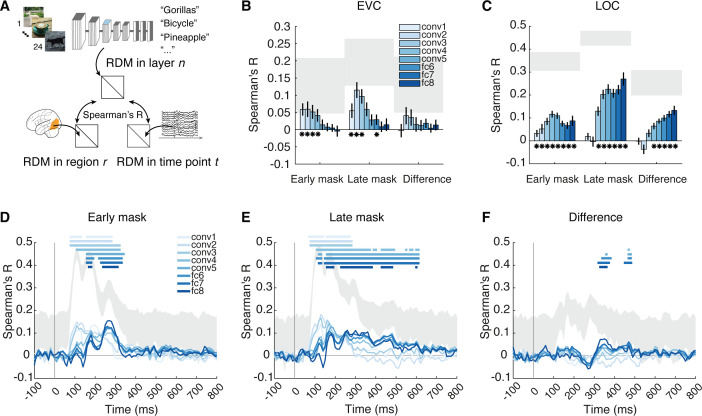
The representational format of visual representations is resolved in space or time. **(A)** Representational similarity analysis (RSA) linking brain responses to layer-wise activation patterns in a convolutional neural network (CNN) model (AlexNet trained on object categorization). We obtained representational dissimilarity matrices (RDMs) for each layer of the CNN, each region of interest in fMRI, and each time point in EEG. We then compared (Spearman’s R) the CNN RDMs with the EEG and fMRI RDMs, respectively. **(B, C)** RSA results linking **(B)** early visual cortex, and **(C)** LOC to CNN layers. For **(B, C)**, significant correlations are marked by black asterisks above bars (*N* = 27, *p* < 0.05, right-tailed permutation tests, FDR-corrected); error bars depict standard errors of the mean; shaded gray areas indicate the noise ceiling. **(D-F)** RSA results linking CNNs to EEG in the **(D)** early mask condition, **(E)** late mask condition, and **(F)** difference therein. For **(D–F)**, significant correlations at time points are denoted by asterisks colored by layer (*N* = 31, right-tailed permutation tests, cluster definition threshold *p* < 0.005, cluster-threshold *p* < 0.05, 10,000 permutations); shaded gray areas represented the noise ceiling. The data underlying this figure can be found at https://osf.io/U3VG9/.

We next investigated how recurrent processing affects the format of visual representations. For this, we used representational similarity analysis (RSA) [43,65] to compare representations in the brain and in the layers of an 8-layer AlexNet CNN model trained on object categorization [66,67] (Fig 3A). We used a feedforward CNN as a tool to examine how feature representations of different complexity emerge across space and time when recurrence is intact or disrupted. The rationale is that correspondence between layers in the CNN hierarchy reflects the complexity of brain representations, with early layers capturing low-complexity features and later layers representing highercomplexity features [68–70].

We began the investigation of the format of visual representations in EVC and LOC using fMRI. We report the significant layers, with a focus on those with the highest correlation, referred to as peak layers. The 95% confidence intervals for the peak range were estimated through bootstrapping (1,000 samples) and shown in brackets. In EVC, we identified significant correspondences with the early to middle CNN layers 1–4. The strongest correspondence was in layer 2 (i.e., conv2, 95% Cis in early mask condition: conv1 to conv4; late mask condition: conv1 to conv3) (Fig 3B). Although the difference between masking conditions appeared largest in the early layers, it was not statistically significant. This suggests that feedforward and recurrent processing in EVC primarily involve the processing of low-level features. A supplementary analysis that compared the visual representations as revealed by the within- and across-conditions decoding to the CNN model showed an equivalent result pattern (S5A Fig), further strengthening this view.

In contrast, in LOC, we observed three key findings that together suggest a shift in representational format from lower to higher visual feature complexity through recurrent activity, indexed by a shift in peak correspondence from early to late CNN layers. First, although both masking conditions showed correspondence with middle to deep CNN layers (layers 3–8; Fig 3C), the peak correspondence differed: in the early mask condition, it occurred in a middle layer (conv4; [conv4, fc8]), whereas in the late mask condition, it shifted to the deepest layer (fc8; [fc8, fc8]). This shift had an effect size of 0.56 (Hedges’ g) and was statistically significant (*p* = 0.039, two-tailed permutation test). Second, comparisons between masking conditions revealed differences in middle to deep layers 4–8, with a peak in the deepest layer (i.e., fc8, [fc7, fc8]). Third, in the early mask condition, LOC activity corresponded to early CNN layers (1–2), but this was not observed in the late mask condition. These patterns were also evident when comparing within- and across-conditions decoding results (S5B Fig). Together, they converge to support that recurrent activity in LOC shifts the representational format from lower to higher feature complexity.

Next, we assessed the change in the representational format of visual representations across time using EEG. We observed correspondence to all layers of the CNN in both masking conditions (Fig 3D and 3E) with a temporal progression in peak correspondence from lower layers early in time to deepest layers later in time [69,71] (for statistical details, see S4 Table). This shows that in both masking conditions, visual representations emerge along a cascaded processing hierarchy characterized by increasing feature complexity [5,20,72,73]. To assess the feature complexity and timing of recurrent processing directly, we determined the difference in correspondence between the masking conditions (Fig 3F). We found that the difference was highest in the middle and deep layers between ~300 ms and 500 ms. This indicates that recurrent activity changes the representational format to higher-complexity features in later time points. Consistent with this conclusion, equivalent results patterns were observed in a supplementary analysis comparing the visual representations revealed by the within- to across-conditions decoding to the CNN model (S5D Fig).

Finally, for both EEG- and fMRI-based analyses, we confirmed the main results pattern using another CNN architecture (i.e., ResNet50 [74], S6 Fig), indicating the generalizability of the conclusions across models.

Together, this shows that recurrent processing leaves the format of EVC representations relatively unaffected in terms of visual feature complexity. In contrast, recurrent processing strongly changes the format of LOC and late representations from lower to higher complexity, revealing the nature of its effect on the representational format.

### The spatiotemporal dynamics of changes in representational format through recurrence

**Fig 4:**
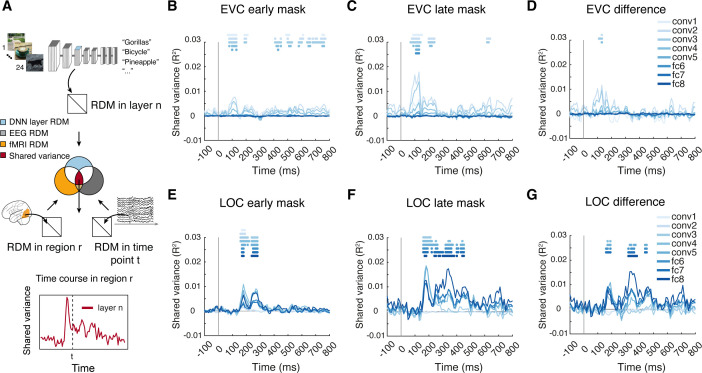
The format of spatiotemporally resolved visual representations. **(A)** Commonality analysis based on representational similarity analysis, linking temporal dynamics (EEG), cortical locus (fMRI), and feature complexity (convolutional neural network [CNN] layers of AlexNet). This yielded time courses of shared variance for each CNN layer in early visual cortex (EVC) and LOC, respectively (here: layer 3 in LOC). **(B–G)** Time courses of shared variance with CNN features in the **(B, E)** early mask condition, **(C, F)** late mask condition, and **(D, G)** difference between them, in EVC **(B–D)** and LOC **(E–G),** respectively. For **(B–G)**, significant effects at time points are denoted by asterisks color-coded by CNN layer (*N* = 31, right-tailed permutation tests, cluster definition threshold *p* < 0.005, cluster-threshold *p* < 0.05, 10,000 permutations). The data underlying this figure can be found at https://osf.io/U3VG9/.

Visual processing evolves dynamically across spatial locations in the brain and across time simultaneously, necessitating a spatiotemporally resolved view [41,75]. However, the analyses so far assessed visual representations and their format separately in space and time. For a fully spatiotemporally resolved view, we used RSA-based commonality analysis [76,77] (Fig 4A), providing time courses of shared variance with each CNN layer in EVC and LOC (for statistical details, see S6 Table).

In EVC, we observed an emergence of visual representations of low- to mid-complexity with peaks early in time, predominantly at 120–130 ms, both in the early mask condition and the late mask condition (CNN layers 1–6, Fig 4B and 4C). The difference between masking conditions emerged early (peaks at ~90–130 ms) and was in low-to-middle complexity, too (CNN layers 1–5, Fig 4D). This shows that recurrent activity impacts visual representations in EVC early in time and in a low-to-mid-complexity format.

In LOC, we observed the emergence of visual representations of all complexity levels at a later stage than in EVC, with two peaks at ~200 ms and 300 ms in both masking conditions (Fig 4E and 4F). The difference between masking conditions was in features of middle-to-high complexity (CNN layers 4–8, Fig 4G). This shows that recurrent activity impacts visual representations in LOC later in time and in a mid-to-high complexity format.

In sum, recurrent activity modulates EVC representations early in processing in low-to-mid complexity format, and LOC representations later in processing in mid-to-high complexity format.

### The spectro-temporal basis of recurrent processing

**Fig 5:**
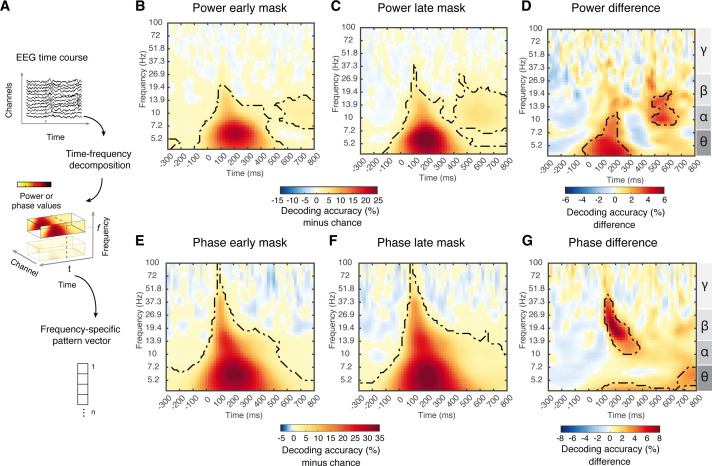
Spectral characteristics of visual representations. **(A)** Using time-frequency decomposition we extracted frequency-specific response pattern vectors across EEG channels for power [63] and phase values (63 × 2 = 126) separately. **(B–G)** Results of time- and frequency-resolved object identity decoding in the **(B, E)** early mask condition and **(C, F)** late mask condition, and **(D, G)** difference between them, based on power values **(B–D)** and phase values **(E–G)**. For **(B–G)**, chance level was 50%; time-frequency combinations with significant above-chance decoding are outlined by black dash lines (*N* = 31, right-tailed permutation tests, cluster definition *p* < 0.005, significance *p* < 0.05, 10,000 permutations); the vertical gray line indicates stimulus onset, and the right y–axis labels indicate frequency bands. The data underlying this figure can be found at https://osf.io/U3VG9/.

The transmission of visual information in feedforward and recurrent fashion is fundamentally indexed by neural activity in distinct frequency bands [78,79]. Based on previous work in human and nonhuman primates, we hypothesized that recurrent processing should be evident in the low-frequency range between the theta- and the beta range [80–83]. Thus, in the next step, we investigated the spectral characteristics of visual processing in the early mask condition and the late mask condition. For this, we decoded object identity from EEG data resolved both in time and frequency (Fig 5A), considering power and phase of the signals separately.

Across both masking conditions and for both power (Fig 5B and 5C) and phase (Fig 5E and 5F), we observed significant object decoding in a broad frequency range. The decoding peak was consistently within the theta band (~6 Hz) at ~200 ms (for statistical details see S7 Table). This establishes the sensitivity of the analysis and warrants further inspection by contrasting the masking conditions.

Comparing the results of the early mask condition to the late mask condition, we observed four components with distinct temporal and spectral characteristics (Fig 5D and 5G; for statistical details see S7 Table). Two clusters were in the power domain (Fig 5D) and two in the phase domain (Fig 5G). In detail, in the power domain, there was a cluster before 300 ms in the theta–alpha frequency range (peak at 4.27 Hz, 160 ms), and a later cluster after 400 ms in the alpha–beta frequency range (peak at 10.72 Hz, ~ 540 ms). In the phase domain, there was a cluster between 100 ms and 400 ms in the alpha–beta frequency range (peak at 19.35 Hz, 200 ms) and a cluster in the theta range across the entire temporal range after stimulus onset (peak at 10.03 Hz, 560 ms). A supplementary analysis comparing the within- and across-conditions decoding (S7 Fig) revealed a more widespread effect that largely encompassed the clusters observed here.

Together, this establishes the spectro-temporal basis underlying recurrent visual processing as four distinct components with specific spectro-temporal profiles.

## Discussion

We combined a backward masking paradigm with multivariate analysis on EEG and fMRI data, along with computational model comparison as a tool, to characterize when, where and how recurrent processing affects object representations. Harvesting the detailed structure of visual representations beyond grand-average responses to visual stimulation, we showed that recurrence substantially affects the image-specific geometry of visual representations.

First, regarding the spatiotemporal dynamics, we found that recurrence affects visual representations across the ventral visual stream, early on at ~100 ms in EVC and in two later phases of ~175 ms and 300 ms in LOC, adding persistent rather than transient neural dynamics to visual processing. Next, we determined the feature complexity and spectral basis of the effect of recurrence on visual representations. We found that recurrence shifts the feature format in LOC from mid- to high-level feature complexity and is mediated by four distinct spectro-temporal components in the theta to beta frequency range.

### Backward masking as a tool to dissect recurrent processing

A key assumption on which our interpretations rest is that the difference between early and late mask conditions in neural activity isolates recurrent processing to a relevant degree. While not undoubted [84,85], this assumption is supported by a large number of studies linking backward masking to recurrent rather than feedforward processing [23,31,35,37,86], particularly affecting interregional and feedback communication within visual areas [37,40,87].

An alternative explanation is that the early mask condition yields a lower signal-to-noise ratio (SNR) than the late mask condition, thus confounding our results. However, the supplementary within-condition versus across-condition decoding analyses show that within-condition decoding in the early mask condition remains robust, whereas across-condition decoding between early and late masks relatively deteriorates (S1D, S3D–S3F, and S4B Figs). This pattern suggests that the SNR in the early mask condition allows for robust decoding, so that the observed effects are plausibly attributable to altered (i.e., disrupted) recurrent processing rather than SNR differences.

Our results invite future backward masking studies employing multivariate analysis to further confirm and dissect the sources of recurrent activity identified here. This might in particular involve causal interventions such as transcranial magnetic stimulation (TMS) [88] to determine the sources of recurrent activity across cortex, and layer-specific fMRI analysis [89–91] to distinguish recurrent from feedforward processing based on cortical layers [1,2,92].

### The spatiotemporal dynamics of recurrent processing

Our separate analyses of EEG and fMRI data revealed a broad impact of recurrent processing: it affects visual representations starting at 100–120 ms, with a peak at 200 ms in a wide plateau, and across the ventral visual stream.

The combination of EEG and fMRI dissected these broad effects into distinct components for EVC and LOC. In EVC, recurrence affected visual representations rapidly, with a peak effect at 100 ms. This is in the range of previously observed early effects of recurrence in nonhuman primate EVC [7], associated with contextual modulation and figure-ground segregation [93–95], where feedback signals originate from within the ventral visual stream. Our study does not allow for dissecting different types of recurrence, and the observed effects could stem from recurrent activity within (early) visual cortex but also from rapid long-range feedback from beyond visual cortex [33,96].

In LOC, recurrence affected visual representations later, with two peaks at ~175 ms and 300 ms. The earlier peak at 175ms is consistent with effects of masking observed in monkeys invasively in V4/pIT [35], potentially originating from prefrontal cortex [39,97] and modulating visual activity in monkey V4 and IT [40,98]. The later peak at 300 ms might reflect pattern completion, as indicated by delayed responses in invasive studies of human IT in a similar time frame [14,38]. The origin of this late effect might be medial temporal lobe regions such as parahippocampal cortex that activate as early as 270 ms after stimulus onset [6,99,100]. Alternatively, attentional effects might be driving the late effect, consistent with reports of human and nonhuman attentional modulation in high-level ventral visual cortex starting at 150 ms [7,101–104].

Our results cannot ultimately determine whether nonvisual regions contributed to the observed effects, as here, fMRI coverage was restricted to the ventral visual stream. Future research assessing the whole brain, including frontal [40,105–107] and parietal [108–111] regions, is needed.

Temporal generalization analysis added further insights that recurrence specifically contributed to the emergence of persistent, rather than transient representations. This is consistent with the observation that masking reduces firing duration in single cells in monkey IT, [112,113] and that masking reduces persistence in the visual representations of occluded objects in humans [12]. Together, this supports the view that recurrence plays an active role in accruing and maintaining important information online for further processing and decision-making [51,114–118].

A limitation of our work is that it characterizes visual representations without directly linking them to object recognition. Further work should relate the observed effects to behavior, for example, by contrasting correct and error trials [119,120] or linking neural activity to behavior on a trial-by-trial basis [121,122].

### Recurrence transforms the feature format in LOC from mid- to high-level complexity

Using a feedforward CNN to assess feature complexity, we found that recurrence modulates representations in the lateral occipital cortex (LOC) but not in EVC.

In LOC, we observed a shift of representational format from predominantly mid-level to more high-level features through recurrent processing. This has three implications. First, it adds algorithmic specificity to the observations from invasive recordings in nonhuman primates that feature coding in high-level ventral visual cortex is dynamic, changing the code over time from global to fine-grained [123], individual object parts to multipart configuration [124], and from a code supporting detection to one for discrimination [125]. Second, it qualifies the finding that masking affects firing rate and stimulus specificity in monkey IT [34,112], linking those observations to the lack of recurrent activity mediating high-complexity features [10,40]. Finally, it converges with visual imagery and working memory studies indicating that recurrent processing carries high-complexity features [47,126]. However, a limitation of our finding is that we cannot distinguish whether the observed effect indicates the addition of new features to LOC representations through recurrence that are absent in feedforward processing [16], or the modulation of the gain of already present features, e.g., through attention [127–130].

In contrast to LOC where we observed a shift in the peak layer to features of higher complexity through recurrence, we did not find evidence for a change in feature complexity in EVC from its low-level complexity format (Fig 3B and 3C): analogous to the case of LOC, this suggests two different mechanisms underlying recurrence in EVC. One is that recurrent activity in EVC amplifies features encoded already in the feedforward sweep [19]. The other is that it adds new features of low-level complexity, consistent with observations of dynamical feature coding in orientation and color [131,132] and changes to receptive field structure [133]. To distinguish these potential mechanisms of recurrence in both LOC and EVC, future work is needed, for example, investigating the finer-grained encoding of single features rather than feature complexity [134,135] and modulating attentional state [136–138].

Please note that here we used feedforward CNNs as an established tool to quantify visual feature complexity [68–70], rather than as a model of recurrent human visual processing. For modeling recurrent visual processing, recurrent CNNs [10,139–141] are needed. This is a burgeoning research field, delineating how feedforward and recurrent activity [21,141,142] respectively account for core object recognition [16], as well as visual behavior [140]. We believe our empirical data could serve as an interesting benchmark for model fitting.

### The spectral basis of recurrent processing

Our results extend prior work by identifying four distinct spectro-temporal components (theta–beta range) with specific temporal profiles that subserve recurrent processing [45,143–145]. Our findings refine the view that low-frequency rhythms may generally serve as a neural index for recurrent processing [81,82] by showing that recurrent processes can further be subdivided into early recurrent processes (in the phase domain), followed by later recurrent processes (in the power domain).

Our results further support the broad notion that theta [146], alpha [82,147] and beta [80–82,148] frequencies mediate recurrent activity and play an active role in cognition [149–152] and vision in particular [47,153–155], rather than in inhibition of irrelevant information [147,156] or cortical idling [157,158].

While our spectral analysis reveals robust stimulus-related changes in power and phase, it is important to note that these spectral signatures may reflect phase-locked evoked transients rather than intrinsic, non-phase-locked oscillatory dynamics. Thus, although we observe spectral modulations, future work is needed to disentangle oscillatory from nonoscillatory components [57,159,160] underlying these effects.

Our main contribution lies in identifying four distinct spectro-temporal components, thereby refining our understanding of the spectral basis of recurrent processing. Future work is required to characterize these components in more detail (for a supplementary analysis linking the components to EVC and LOC and clarifying their feature format, see S8 Fig), and to determine whether they serve distinct functional roles.

## Conclusions

In sum, recurrent activity substantially affects the ventral visual stream, first in EVC and subsequently in LOC. Recurrent processing drives a shift in the feature format of LOC from mid- to high-level complexity and is linked to distinct spectro-temporal components in the theta to the beta frequency range. These findings characterize where, when, and how recurrence affects visual representations, furthering the understanding of how the recurrent information flow in the brain mediates visual object perception.

## Materials and methods

### Participants in EEG and fMRI experiments

We conducted two independent experiments: an EEG and an fMRI experiment. Thirty-two participants took part in the EEG experiment, of whom one was excluded due to high-frequency noise in the recordings (*N* = 31, mean age 26.6 years, standard deviation 4.8 years, 20 female). Twenty-eight participants took part in the MRI experiment, of whom one was excluded due to failure of the stimulus presentation equipment (*N* = 27, mean age 27.7 years, standard deviation 4.6 years, 19 female). There was an overlap of four participants between the EEG and the fMRI participant sample. All participants had normal or corrected-to-normal vision. The study was conducted according to the Declaration of Helsinki, with the exception that it was not preregistered, and approved by the ethics committee of the Department of Education and Psychology at Freie Universität Berlin (protocol approval number: 021.2023). Written informed consent was obtained from all participants prior to data collection.

### Stimulus set

The stimulus set consisted of a set of target object images and a set of image textures used to create dynamic object masks.

The set of target object images consisted of 24 object images (Fig 1A). Each image showed an object of a different object category and was cropped quadratically to the size of the centrally presented object. The 24 object images were a subset of a larger set of 118 images [161]. The rationale for selecting the stimulus subset was as follows. Brain responses to natural images are typically highly correlated across the stages of the visual processing hierarchy. That is, two images that elicit similar responses at one stage tend to elicit similar responses at another stage, too. This makes assessing the role of different processing stages and the information they send in a forward or backward direction using multivariate analysis methods particularly difficult: due to the high correlations observed, experimental effects cannot be uniquely assigned to particular stages. To improve the chances of eliciting dissociable responses across the visual processing hierarchy in our experiment, we selected the stimulus set that yielded low correlations between the entry EVC and the endpoint (inferior temporal cortex, IT) of the ventral visual pathway. For this, we used fMRI data in EVC and IT for the 118-image superset from a previous experiment [161]. We assessed the similarity of representations in EVC and IT for the 118 images using RSA [43,65]. To select 24 images that yielded uncorrelated responses, we used a genetic algorithm [162] for optimization. In detail, the optimization constraint was to minimize the absolute value of correlation between EVC and IT representational dissimilarity matrices (RDMs). The RDMs for the chosen 24-stimulus set yielded the desired low similarity between EVC and IT (*R* = 0.0018) on the preexisting fMRI data set. In comparison, this was lower than a random selection of 24 stimuli would have been (as assessed by 1,000 random draws, average *R* = 0.211, standard deviation = 0.101).

We created a set of image textures to be used for dynamic backward masks. For this, we chose a different subset of 24 object images randomly from the 118-image set. Each selected image was converted into a texture using the parametric synthesis method described in [163]. This algorithm analyzes and matches various spatial statistics (e.g., orientation, scale, phase correlations) in overlapping patches of the original source images, ensuring that the texture of the resulting image preserves similar color, luminance, and local orientation distributions as the source while discarding coherent shape cues. We then visually confirmed that none of the resulting textures contained identifiable objects. Finally, to create each dynamic mask, we randomly selected 12 textures from our pool of 24 and arranged them in a random sequence, yielding 24 distinct 12-frame masks.

### Experimental procedures

#### Main experiment and experimental design.

We presented object images to participants in a backward masking paradigm (Fig 1B). The general experimental design, stimulus presentation parameters, and trial structure were equivalent in both the EEG and the fMRI experiments. We describe the crucial elements common to EEG and fMRI first before detailing the modality-specific differences.

On each trial, a single object image (referred to as “target”) was briefly displayed for 17 ms, followed by a 200 ms dynamic mask. Object images and dynamic masks were randomly paired for each trial. We manipulated the target’s visibility by varying the inter-stimulus interval (ISI) between the target and mask. This defined two conditions: in the early mask condition, the ISI was 17 ms; in the late mask condition, the ISI was 600 ms. Note that the late mask condition, until 600 ms, corresponds to a condition without a mask.

During each trial, one of the 24 dynamic masks was presented. Stimuli were presented centrally on a gray background with a size of 5 × 5 degrees visual angle, overlaid with a bull’s-eye fixation symbol with a diameter of 0.1-degree visual angle [164]. The texture images of dynamic mask were positioned and sized identically to the target object images. Participants were instructed to fixate on the fixation symbol throughout the experiment. We used Psychophysics Toolbox [165] for experimental presentation. In the EEG experiment, stimuli were presented on a Samsung 2233RZ monitor with a refresh rate of 60 Hz. In the fMRI experiment, stimuli were projected using a Canon SX60 multimedia projector, also at 60 Hz.

#### EEG experimental procedures.

In the EEG experiment, participants completed a total of 2,544 main trials partitioned into 26 blocks of 3.5 min each. Throughout the experiment, each object image was presented a total of 53 times in both the early mask condition and the late mask condition.

We assessed the participants’ recognition performance with additional task trials that were interspersed every 4–6 (average 5) main trials. The task was to identify the object image in the previous trial from a pair of images in a 2-AFC task. For this, two images were presented side by side for 500 ms: one of the images presented was the image from the previous trial, and the other image was randomly chosen from the remaining 23 images. Participants indicated their response with a button press.

Participants were instructed to refrain from blinking throughout the experiment except during the additional interspersed task trials, when participants were asked to blink when they gave their responses. While the inter-trial interval (ITI) between main trials was between 900 ms and 1,100 ms, following the 2-AFC trial, the ITI was extended to 2,000 ms to prevent motor artifacts from influencing the EEG recordings of the subsequent trial.

Participants had high task performance in both masking conditions, suggesting that they attended to the stimuli even under viewing challenging conditions (for details and statistics, see S8 Table). Further, as expected, the task performance was worse for the early mask condition than for the late mask condition trials. This confirms the efficacy of the backward masking procedure in reducing object visibility.

#### fMRI experimental procedures.

In the fMRI experiment, participants performed a total of 12 runs, each lasting 6.5 min. In each run, each object image was presented twice in the early mask condition and the late mask condition, resulting in 96 main trials per run. The trial-onset synchrony was 3,000 ms. Main trials were interspersed with null trials (34 per run), during which only the background, but no stimulus was shown.

Participants were instructed to attend to the object images and respond with a button press if an object image was repeated in two consecutive trials (i.e., a one-back task on the target images). Object repetitions occurred 10 times per run. As in the EEG experiment, participants had overall high task performance, with worse performance for the early mask condition than for the late mask condition trials (for details and statistics, see S8 Table).

#### fMRI localizer experiment.

To define the ROIs EVC and object-selective lateral occipital cortex (LOC), we performed a separate fMRI localizer run. The localizer run was conducted prior to the fMRI main experiment runs. The stimulus set comprised 40 images of objects and scrambled objects each.

The localizer run used an fMRI block design. Each block lasted 15 s. During each block, 20 stimuli were centrally presented within an area of 5 × 5 degrees visual angle at a rate of 650 ms on and 100 ms off. There were 6 object- and scrambled-object-blocks each. They were presented in counterbalanced order and randomly interspersed with 7 baseline blocks during which only the background was shown.

Participants were instructed to fixate on a centrally presented fixation symbol that was presented throughout the experiment, and to respond to one-back repetitions of images with a button press. Repetitions occurred a total of 9 times over the course of the localizer experiment.

### EEG data acquisition, preprocessing, and time-frequency decomposition

We recorded EEG data using an ActiCap 64 electrodes system and a Brainvision actiChamp amplifier. Sixty-four electrodes were placed according to the 10−10 system, with an additional ground electrode and a reference electrode placed on the scalp. The signals were sampled at a rate of 1,000 Hz and online filtered between 0.03 and 100 Hz. All electrodes’ impedances were kept below 10 kΩ during the recording.

We preprocessed EEG data offline using the Brainstorm-3 toolbox [166]. We removed noisy channels (average 2.2 channels per participant, standard deviation 1.8 channels) identified through visual inspection. We then filtered the data with a low-pass filter at 40 Hz. Eyeblinks and eye movement artifacts were detected using independent component analysis. We visually inspected the resulting components and removed those resembling the spatial properties of eyeblinks and eye movements (average 2.7 components per participant, standard deviation 0.9 components). We segmented the continuous data in epochs between −200 ms and 800 ms with respect to the target image onset and baseline-corrected the segmented data by subtracting the mean of the 200 ms interval before stimulus onset from the entire epoch. We finally applied multivariate noise normalization on the preprocessed data to improve the SNR and reliability of the data [167]. This formed the data for the temporally resolved decoding analyses.

For time-frequency analysis, we preprocessed the data again in the same way except for two differences: 1) we did not apply offline filtering, and 2) we segmented the continuous data into longer epochs (−600 ms to 1,200 ms) to enable better estimation of signals at lower frequencies.

#### Time-frequency decomposition of the EEG data.

We performed time-frequency decomposition by applying complex *Morlet* wavelets. The wavelets, resembling complex sine waves modified by a Gaussian function, covered frequencies from 4 Hz to 100 Hz in 50 logarithmically spaced increments. The Gaussian taper characteristics varied across this frequency range, with temporal full-width-half-maximum (FWHM) ranging from 20 ms to 500 ms as frequency decreased and spectral FWHM ranging from 1 Hz to 31 Hz as frequency increased.

We applied the complex *Morlet* wavelets for each channel and each trial of the EEG data at 2 ms intervals (i.e., 500 Hz). At each time point, this yielded 50 distinct frequency coefficients corresponding to the range of 4–100 Hz. At each time-frequency point, we computed two measures: the power and phase of the oscillation. To determine the absolute power values, we took the square root of the resulting time-frequency coefficients. To determine the phase values, we determined the real (sine) and imaginary (cosine) components from the time-frequency coefficients. This decomposition procedure yielded frequency-resolved EEG signals to be used for further time-frequency resolved decoding analyses. To decrease computation time and disk space usage, we downsampled the time points of frequency-resolved signals at 20 ms intervals after time-frequency decomposition.

### fMRI data acquisition, preprocessing, and univariate analysis

We acquired T2* and T1-weighted MRI data using a 3T Siemens Tim Trio scanner with a 32-channel head coil. We acquired T2*-weighted BOLD images using a gradient-echo Echo-planar imaging (EPI) sequence. The acquisition parameters were as follows: TR = 2,000 ms, TE = 30 ms, FOV = 224 × 224 mm^2^, matrix size = 112 × 112, voxel size = 2 × 2 × 2 mm^3^, flip angle = 70°, with 30 slices and a 20% gap. The acquisition volume covered the occipital and temporal lobes and was oriented parallel to the inferior temporal cortex. Additionally, we obtained a T1-weighted image for each participant as an anatomical reference (MPRAGE; TR = 1,900 ms, TE = 2.52 ms, TI = 900 ms, matrix size = 256 × 256, voxel size = 1 × 1 × 1 mm^3^, and 176 slices).

We performed fMRI data preprocessing using SPM12 (https://www.fil.ion.ucl.ac.uk/spm/software/spm12/). This involved realignment, slice-time correction, co-registration to the anatomical image, and normalization to Montreal Neurological Institute (MNI) space. For the fMRI data of the localizer experiment, but not the main experiment, we additionally applied smoothing with a Gaussian kernel (FWHM = 5 mm). For the fMRI data from the main experiment, we additionally estimated noise components using the Tapas PhysIO toolbox [168,169] by creating tissue-probability maps from each participant’s anatomical image and extracting noise components from the white matter and Cerebrospinal fluid (CSF) maps combined with the fMRI time series.

We used general linear models (GLMs) to estimate responses for the 48 experimental conditions (i.e., the 24 object images presented in either the early mask condition or the late mask condition) in the main experimental runs and responses for 3 experimental conditions blocks (i.e., objects, scrambled objects, and baseline) in the localizer runs.

The analysis was conducted in a participant-specific fashion. In all cases, we applied the GLM estimation to the preprocessed fMRI data for each run. We entered experimental condition onsets and durations as regressors into the GLM. Nuisance regressors comprised noise components and movement parameters. Our workflow involves two separate hemodynamics response function (HRF) modeling procedures using GLMs.

First, for the experimental runs with an event-related design, we used voxel-specific HRF modeling. To capture regional and individual variability in BOLD timing, we evaluated 20 different GLMs by convolving regressors with 20 distinct HRFs as derived from a large fMRI dataset [170] for each voxel. We then identified the HRF that resulted in the lowest average residual [171] per voxel and chose the corresponding estimates for further analysis.

Second, for the localizer run with a block design, we used the canonical HRF. We computed two contrasts from the resulting GLM parameter estimates that were used at a later step for voxel selection in the ROI analysis. The first contrast was defined as object + scrambled objects > baseline to define EVC. The second contrast was defined as objects > scrambled objects to define LOC. This yielded two t-value maps for the localizer run per participant.

#### Definition of fMRI ROIs.

For each participant, we identified two ROIs within the ventral visual stream: EVC and lateral occipital complex (LOC). To determine the boundaries of these ROIs, we used participant-specific t-value maps from the localizer run threshold at *p* < 0.0001 intersected with anatomical masks. For the EVC definition, we intersected the thresholded t-value map (object + scrambled objects > baseline) with the combined anatomical region masks of V1, V2, and V3 obtained from the Glasser Brain Atlas [172]. For the LOC definition, we intersected the thresholded t-value map (objects > scrambled objects) with a mask of LOC derived from a functional atlas [173]. We removed any voxels shared between the EVC and LOC ROIs to avoid overlap. This process resulted in the definitions of two ROIs for each participant.

### Multivariate pattern analysis on EEG and fMRI data

An analytical challenge in comparing neural activity evoked by target images versus target image with a backward mask is the confounding effect introduced by the mask. Previous studies addressed this challenge by using subtraction design, for example, by including trials showing only the mask and subtracting the resulting neural activity from the neural activity evoked by the stimulus plus mask [31,174]. Here, instead, we used a content-sensitive multivariate pattern analysis on EEG and fMRI data to dissect neural activity of the target image from neural activity evoked by the mask. The rationale is that in our design, target and mask stimuli were statistically independent, so multivariate pattern analysis classifying target object images revealed neural activity related to object images rather than the mask.

We performed multivariate pattern analysis on EEG and fMRI data using linear support vector machines [175] as implemented in the LIBSVM toolbox [176] in MATLAB (2021a). We conducted all analyses on a participant-specific basis.

#### Temporally resolved decoding analysis from EEG data.

To determine when the brain processes object information, we conducted a time-resolved decoding analysis [51,177]. We examined EEG data from −200 ms to 800 ms with respect to target image onset, in 10 ms intervals. At each time point, we extracted trial-specific EEG channel activations and arranged them into 64-dimensional pattern vectors for each of the 24 object image conditions for each masking condition, separately. We conducted two types of analysis: within- and across-masking conditions object decoding.

In the within-masking condition analysis, we separately decoded object conditions for the early mask and the late mask conditions. For each of the 24 image conditions, we first randomly grouped trials into four equally sized bins and averaged them to create four pseudo-trials to enhance the SNR. Employing a leave-one-out cross-validation approach, we then divided these pseudo-trials into training (three pseudo-trials) and testing sets (one pseudo-trial) to pairwise decode object identity. We then decoded object conditions pairwise for all object condition combinations. The resulting decoding accuracies were arranged into a 24 × 24 decoding accuracy matrix, with rows and columns corresponding to the decoded object conditions. This matrix is symmetric across the diagonal, with the diagonal being undefined. We repeated this analysis 100 times, randomly assigning trials to pseudo-trials each time. Averaging results over repetitions yielded one 24 × 24 decoding accuracy matrix for each time point, separately for the early and late mask conditions.

In the across-masking conditions analysis, we proceeded accordingly, but assigned pseudo-trials to the training set and testing set from different masking conditions. That is, we trained on data recorded in the early mask condition and tested on data from the late mask condition (or vice versa). We averaged the results across both training and testing directions. This yielded one 24 × 24 decoding accuracy matrix for each time point.

In both analyses, averaging across the 24 × 24 entries of decoding accuracy at each time point resulted in a grand-average decoding accuracy time course.

#### Time-generalization analysis (TGA) using decoding.

We used time-generalization analysis (TGA) to determine how visual representations relate to each other across different time points. We proceeded as for the within masking condition time-resolved decoding analysis, except that classifiers trained on data from a particular time point were tested iteratively on data from all other time points. The rationale here is that successful generalization across time points indicates the similarity of visual representations over time. This analysis yielded 24 × 24 decoding accuracy matrices for each combination of time points from −200 ms to +800 ms. By averaging the entries of each decoding accuracy matrix across time point combinations, we obtained a temporal generalization matrix (TGM), where rows and columns are indexed by training and testing time points, respectively. As the training and testing directions have no interpretable meaning in this context, we symmetrized the TGM by averaging both train-test directions.

#### Time-frequency-resolved decoding analysis from EEG frequency power and phase.

To determine the spectral properties of visual object representations in the two masking conditions, we conducted a time-frequency-resolved decoding analysis. This analysis was identical to the time-resolved analysis described above, but instead of decoding from raw activation values, we decoded object identity from patterns of power or phase value. We performed the analysis separately for 50 frequency bins spanning from 4 Hz to 100 Hz, using either power or phase values. In the power-based analysis, decoding was based on 64 power values corresponding to the 64 EEG channels. For the phase-based analysis, decoding used 128 values corresponding to the concatenation of the 64 sine and 64 cosine values. This resulted in one 24 × 24 decoding accuracy matrix for each time point and frequency bin, for the power- and phase-based analyses. Averaging across the 24 × 24 entries of decoding accuracy resulted in a grand average time-frequency matrix, where time points and frequency bins are indexed in rows and columns, respectively.

#### Spatially resolved decoding analysis from fMRI data.

We conducted two types of decoding analyses on the fMRI data: ROI-based and volumetric searchlight-based decoding [63,64] on the fMRI data.

For the ROI-based analysis, we arranged beta values from voxels of a given ROI into pattern vectors for each of the 24 experimental conditions and each of the 12 runs of the main fMRI experiment. To enhance SNR, we grouped 3 runs into 4 bins and averaged across runs, creating four pseudo-run fMRI pattern vectors [178]. Then, for each ROI, we performed object decoding on these pseudo-run fMRI pattern vectors in a leave-one-pseudo-run-out manner. Averaging across iterations yielded a 24 × 24 decoding accuracy matrix for each ROI, participant, and masking condition.

For the searchlight-based analysis, for each voxel in the 3D fMRI volume, we defined spheres of voxels around it with a radius of four voxels. For each sphere, we arranged voxel values into pattern vectors. We then decoded object identity as described for the ROI-based analysis. This yielded a 24 × 24 decoding accuracy matrix for each voxel in the 3D fMRI volume for each participant and each masking condition.

In both ROI and searchlight-based analyses, averaging across the 24 × 24 entries of decoding accuracy resulted in either a single value or a 3D map of grand average decoding accuracy, respectively.

### Representational similarity analysis (RSA)

RSA is a framework to relate representations across different measurement and signal spaces, such as those defined by different brain imaging modalities (EEG and fMRI) or computational models [43,65]. The idea is to abstract from incommensurate measurement spaces into a common similarity space where representations can be directly compared.

For each masking condition, the analysis proceeded in two steps. In the first step, within each signal space of interest (e.g., fMRI responses in ROI, EEG broadband responses at particular time points, EEG spectral responses at time-frequency combinations, and activations of CNN layers), we calculated the dissimilarity between condition-specific multivariate activity patterns for all pairwise combinations of the 24 object conditions. We aggregated the results in RDMs, where rows and columns were indexed by the 24 object conditions. These RDMs summarize the representational geometry within each signal space. In the second step, we compared the RDMs across signal spaces using Spearman correlations, yielding a measure of their similarity. We provide the details for each of the two steps below.

#### Step 1: Construction of RDMs.

For the brain data, we used the decoding accuracy matrices resulting from the decoding analyses detailed above as RDMs. This yielded RDMs a) from the temporally resolved EEG decoding analysis for each time point, b) from the time-frequency-resolved EEG decoding analysis for every time point and frequency combination, separately for power and phase, and c) from the spatially resolved fMRI decoding analysis for each ROI.

For the computational model, we built RDMs from an AlexNet architecture trained for object categorization on the ImageNet dataset [66,67]. AlexNet is an 8-layer CNN commonly used as a baseline for brain-CNN comparisons [179]. We fed our object stimuli into the pretrained AlexNet and extracted the activation patterns for each stimulus from each of the five convolutional layers (conv1 to conv5) and the three fully connected layers (fc6, fc7, and fc8).

To test the generalizability of our conclusion across different CNN models, we also built RDMs using the ResNet50 architecture [74], pretrained on the ImageNet dataset [66] for object categorization. ResNet50 features a distinct architecture compared to AlexNet, consisting of an initial convolutional layer followed by four residual blocks, each containing multiple convolutional layers with skip connections, and leading to a final classification layer. We fed the object stimuli into ResNet50 and extracted the activation patterns for each stimulus from the last layer of each of the four residual blocks (block1 to block4) as well as from the final classification layer (fc).

We quantified the dissimilarity of the activation patterns by calculating 1-Pearson’s R for each pair of stimuli. This resulted in eight RDMs for AlexNet layers and five RDMs for ResNet50 layers.

#### Step 2a: Standard RSA—Relating CNN RDMs to EEG and fMRI RDMs.

To characterize the format of neural representations, we related CNN RDMs from each layer to EEG and fMRI RDMs (Fig 3A). The idea is that ascending layers of a CNN capture features of increasing complexity. Thus, relating neural representations to each CNN layer informs about the feature complexity of the neural representations [48–50].

For the EEG-based analysis, we correlated the CNN RDMs with EEG RDMs across all time points obtained from temporally resolved EEG decoding analysis. This yielded a time course of correlation values for each CNN layer, participant, and masking condition. For the fMRI-based analysis, we correlated the CNN RDMs with RDMs from two ROIs (i.e., EVC and LOC), yielding a correlation value per ROI for each CNN layer, participant, and masking condition.

#### Step 2b: Commonality analysis—shared variance among EEG, fMRI, and CNN RDMs.

To investigate the temporal dynamics of specific visual features emerging in brain regions, we extended standard RSA to commonality analysis [76,77] (Fig 4A). Specifically, we computed the coefficients of shared variance separately among EEG RDMs at each time point, fMRI RDMs in each ROI, and CNN RDMs for each layer. This resulted in a time course of shared variance (R^2^) for each CNN layer, ROI, participant, and masking condition.

#### Noise ceilings.

We calculated an upper and lower bound for the noise ceiling [65], i.e., the maximal correlation in the RSA analyses that might be achieved given the noisiness of the data. This was done for the EEG data and fMRI data (i.e., ROIs) separately. To estimate the lower bound, we correlated each participant’s RDM with the average RDM of all other participants. To estimate the upper bound, we correlated each participant’s RDM with the average RDM of all participants. We averaged the results, thus obtaining estimates of the lower and upper noise ceilings for each EEG time point or time point and frequency combination, as well as for all fMRI ROIs.

### Statistical analyses

We used sign permutation tests [180] that do not make assumptions about the data distribution. We compared the statistics of interest (i.e., decoding accuracies minus 50% chance level, correlation coefficients in RSA, coefficients of shared variance in commonality analysis, and accuracy or coefficient differences between conditions) against the null hypothesis that the statistic was zero or less. To obtain a null distribution, we multiplied participant-specific data randomly by either +1 or −1 and computed the statistic of interest for 10,000 permutations. *p*-values were calculated by comparing the observed statistic to this null distribution. These tests were one-sided (right-tailed), reflecting the directional hypothesis that the observed values were greater than zero.

To correct for multiple comparisons with a small number of unrelated comparisons, we used FDR correction at a *p* < 0.05 [181]. In cases involving a large number of comparisons in contiguous and correlated results (i.e., time points, frequencies, or voxels), we used cluster-based inference [182]. For the cluster-size-based inference, we calculated the statistic of interest both for the empirical results and for each permutation sample under the null hypothesis. This resulted in 1-dimensional (e.g., decoding time courses, RSA-based correlation time courses, time courses of shared variance), 2-dimensional (e.g., decoding time-time matrices, decoding time-frequency matrices, RSA-based correlation matrices), or 3-dimensional (i.e., fMRI volumetric decoding results) *p*-value maps. We defined clusters based on temporal or spatial contiguity with a cluster definition threshold at *p* < 0.005. We determined the maximum cluster size for each permutation sample, yielding a distribution of the maximum cluster size statistic. We set the cluster-threshold at *p* < 0.05.

We calculated 95% confidence intervals for the peak latencies or peak layers in the resulting time courses or layer-specific correlations (e.g., decoding time courses, RSA-based correlation time courses, or time courses of shared variance). To do this, we generated 1,000 bootstrap samples by randomly sampling participants with replacements. For each sample, we identified the peak latency or peak layer, yielding a distribution from which we report the 95% confidence intervals.

To assess whether the difference in peak layers between masking conditions was statistically reliable, we followed the sign permutation procedure, generating a null distribution by randomly reassigning condition labels within each participant and recalculating the mean difference across 10,000 permutations. We report the two-tailed p-value and the effect size of the observed difference, calculated using Hedges’ g.

## Supporting information

S1 Fig Temporal dynamics of visual object representations for the two masking conditions. **(A, B)** Temporal dynamics of object representations across categorical boundaries of naturalness **(A)** and animacy **(B)**. **(C, D)** Pairwise object identity decoding results within (green) and across masking conditions (black), along with their differences (brown), are presented separately for the **(C)** late mask condition and **(D)** the early mask condition. Cross-classification results are sorted by training set. For **(A**–**D)**, decoding chance level was 50%; significant above-chance level decoding is denoted by colored asterisks at the corresponding time points (*N* = 31, *p* < 0.05, right-tailed permutation tests, cluster definition threshold *p* < 0.005, cluster-threshold *p* < 0.05, 10,000 permutations); vertical gray line at 0 ms indicates stimulus onset; shaded margins of time courses indicate 95% confidence intervals of the decoding performance determined by bootstrapping (1,000 iterations); horizontal error bars indicate 95% confidence intervals for peak latencies.
(DOCX)

S2 Fig Potential neural source of negative off-diagonal decoding accuracies in temporal generalization analysis. **(A)** Results of time-generalized object identity decoding in the early mask condition, as shown in Fig 1E. Blue and red dots indicate an example pair of corresponding time points for negative off-diagonal decoding accuracies. **(B)** Hypothetical oscillatory mechanism underlying negative off-diagonal decoding. Two identical, time-locked responses for object A and B differ in phase (black solid curve and orange dashed curves). For illustration, the phase shift is set to half a cycle (π), corresponding to the time difference between two off-diagonal time points identified in (A) (blue and red circles). At time point 0π, signal A > signal B, whereas at π, signal B > signal A, illustrating a reversal in relative amplitude. A classifier trained at 0π may thus predict object identity at π with opposite labeling, resulting in below-chance classification accuracy.
(DOCX)

S3 Fig Results of temporal generalization analysis decoding object identity within- and across-conditions. **(A)** Results of time-generalized object identity decoding within the late mask condition (same as Fig 1F). **(B)** Cross-decoding object identity using a classifier trained on the late mask condition. **(C)** The differences between **(A)** and **(B)**. The difference plot reveals positive decoding results in the off-diagonal areas (as shown in the square rectangle in Fig 1G). This occurs due to higher decoding accuracies in the within-condition decoding (late mask) than the across-condition decoding (trained on late mask). This confirms the main results pattern: in the within late mask condition decoding **(A)**, the negative off-diagonal decoding results are veiled by recurrent processes. In the across-conditions decoding **(B)**, results are intermediate between the late mask and early mask results. Subtracting the former from the latter results in positive off-diagonal decoding accuracies. **(D)** Results of temporal generalization analysis decoding object identity within the early mask condition (same as Fig 1E). **(E)** Cross-decoding object identity using a classifier trained on the early mask condition. **(F**) The difference between **(D)** and **(E)**. The difference plot reveals an opposite pattern to the main analysis result (Fig 1G and **(C)**), with negative decoding results in the off-diagonal areas (as shown in the square rectangle as in Fig 1G). This occurs due to lower decoding accuracies in the within-condition decoding (early mask) than across-condition decoding (trained on early mask). This also confirms the main results pattern: in the within early mask condition decoding **(D)**, negative off-diagonal decoding results are not veiled by recurrent processes. In the across-conditions decoding **(E)**, results are intermediate between the late mask and early mask results. Subtracting the former from the latter results in negative off-diagonal decoding accuracies. For **(A–F)**, chance level is 50%. Time point combinations with significantly above-chance level decoding are outlined in black dashed lines (*N* = 31, right-tailed permutation tests, cluster definition threshold *p* < 0.005, cluster-threshold *p* < 0.05, 10,000 permutations); vertical and horizontal gray lines indicate stimulus onsets.
(DOCX)

S4 Fig Results of visual object decoding in fMRI within and across masking conditions. **(A)** Results of object identity decoding in the late mask condition, across-conditions decoding while training on the late mask condition, and the difference between them. **(B)** Results of object identity decoding in the early mask conditions, across-conditions decoding while training on the early mask condition, and the difference between them. **(A)** and **(B)** show a qualitatively equivalent results pattern emerges as in Fig 2C. For **(A, B)**, chance level is 50%; significant above-chance level decoding is denoted by black asterisks above the bars (*N* = 27, *p* < 0.05, right-tailed permutation tests, FDR-corrected); error bars indicate standard errors of the mean.
(DOCX)

S5 Fig Visual features encoded in neural object representations revealed by within- and across-conditions decoding. The analysis rationale is consistent with that used for Figs 1D and 2C. We compared the within-conditions decoding results (averaged across within-early-mask and within-late-mask decoding) to the across-conditions decoding results (averaged across both training and testing directions for cross-decoding). This comparison further determines the direct impact of recurrent activity on visual object representations. **(A, B)** Result of RSA linking object representations in **(A)** EVC and **(B)** LOC to a CNN model trained on object categorization (i.e., AlexNet) as revealed by within-condition decoding, across-conditions decoding, and the difference between them. We observe an equivalent result pattern to the main analysis reported in Fig 3B and 3C. Significant correlations are marked by black asterisks above bars (*N* = 27, *p* < 0.05, right-tailed permutation tests, FDR-corrected); error bars depict standard errors of the mean; shaded gray areas indicate the noise ceiling. **(C–E)** RSA results linking the CNN to EEG for the **(C)** within-condition decoding analysis, **(D)** the across-condition analysis, and **(E)** the difference. We observe an equivalent result pattern to the main analysis reported in Fig 3D–3F (for statistical details, see S5 Table). Significant correlations at time points are denoted by asterisks colored by layer (*N* = 31, right-tailed permutation tests, cluster definition threshold *p* < 0.005, cluster-threshold *p* < 0.05, 10,000 permutations); horizontal error bars indicate 95% confidence intervals for peak latencies, shaded gray areas represented the noise ceiling.
(DOCX)

S6 Fig The representational format of visual representations is resolved in space or time as assessed with ResNet50. We obtained RDMs from the layers of a CNN model (here ResNet50, we used the last layer of each of the four residual blocks and the final classification layer), each ROI in fMRI, and each time point in EEG. We then calculated the correlation coefficients between the CNN layer RDMs and the EEG or fMRI RDMs. **(A, B)** RSA results linking **(A)** EVC and **(B)** LOC to layers of ResNet50. In EVC, the differences between masking conditions were not significant. However, in LOC, the differences between masking conditions revealed a shift in correspondences to deeper layer, i.e., the fc layer, with 95% confidence intervals of (block2, fc). For **(A, B)**, significant correlations are marked by black asterisks above bars (*N* = 27, *p* < 0.05, right-tailed permutation tests, FDR-corrected); error bars depict standard errors of the mean; shaded gray areas indicate the noise ceiling. **(C–E)** RSA results linking layers of ResNet50 to EEG in the **(C)** early mask condition, **(D)** late mask condition, and **(E)** the difference between them. For **(D–F)**, significant correlations at time points are denoted by asterisks colored by layer (*N* = 31, right-tailed permutation tests, cluster definition threshold *p* < 0.005, cluster-threshold *p* < 0.05, 10,000 permutations); horizontal error bars indicate 95% confidence intervals for peak latencies, shaded gray areas represented the noise ceiling.
(DOCX)

S7 Fig Spectral characteristics of visual representations as revealed by within and across masking conditions decoding. The analysis rationale is consistent with that used for Figs 1D and 2C. We compared the within-condition decoding results (averaged across within-early-mask and within-late-mask decoding) to the across-condition decoding results (averaged across both training and testing directions for cross-decoding). This comparison helped determine the direct impact of recurrent activity on visual object representations. **(A–F)** Results of time- and frequency-resolved object identity decoding within-conditions, across-conditions, and their differences. The decoding analyses were based on frequency power values in **(A–C)** and phase values in **(D–F)**. For **(A–F)**, chance level was 50%; time-frequency combinations with significant above-chance decoding are outlined by black dash lines (*N* = 31, right-tailed permutation tests, cluster definition *p* < 0.05, significance *p* < 0.05, 10,000 permutations); the vertical gray line indicates stimulus onset.
(DOCX)

S8 Fig Feature format and cortical origin of the spectral components underlying recurrent processing. **(A)** To investigate where in the brain the specific visual features originate and how each of the four spectro-temporally identified components carries them, we conducted commonality analysis based on RSA linking identified time-frequency resolved dynamics (EEG), cortical locus (fMRI), and feature complexity (CNN layers of AlexNet). We calculated coefficients of shared variance among frequency-based EEG RDMs corresponding to each spectro-temporally identified component, fMRI RDMs within each ROI, and CNN RDMs across each layer. This analysis yielded coefficients of shared variance for each of the four identified components and for each CNN layer in EVC and LOC, respectively. **(B–C)** Shared variance for the two identified power components across brain regions and CNN layers. **(D–E)** Shared variance for the two identified phase components across brain regions and CNN layers. We observed three result patterns, all common across the four components and reinforcing together the outcome of the main analyses. First, we observed significant relationships to CNN layers for all components, regions, and both masking conditions (except for EVC and the alpha–beta power component in the early mask condition), demonstrating the analytical feasibility of the approach. Second, the shared variance was generally lower in the early mask condition compared to the late mask condition, as reported for all main analyses. Third, in relation to EVC the components encompassed representations in low- to mid-level visual feature format as indexed by highest correlations to low and mid CNN layers, whereas in LOC the components encompassed representations in mid to high level visual feature format as indexed by highest correlations to mid and high CNN layers. Significant effects at individual CNN layers are marked with asterisks (*N* = 31, right-tailed permutation tests, FDR-corrected *p* < 0.05, 10,000 permutations). “Diff.” denotes late mask minus early mask.
(DOCX)

S1 Table Statistical details for object identity decoding using EEG signals. (DOCX)

S2 Table Statistical details for object naturalness and animacy decoding using EEG signals. (DOCX)

S3 Table Statistical details for peak locations and spatial extent of top 3 clusters in fMRI searchlight analysis. (DOCX)

S4 Table Statistical details for the RSA results linking the AlexNet model to EEG decoding RDMs within the early and late mask conditions, and the difference between these conditions. (DOCX)

S5 Table Statistical details for the RSA results linking the AlexNet model to EEG decoding RDMs within-, across-conditions, and the difference between them. (DOCX)

S6 Table Statistical details for RSA-based commonality analysis results linking RDMs from EEG, fMRI, and the AlexNet model. (DOCX)

S7 Table Statistical details for object identity decoding using spectro-temporally resolved EEG signals. (DOCX)

S8 Table Behavioral performance during EEG and fMRI experiments. (DOCX)

## Abbreviations

2-AFC: 2-alternative forced-choice
CNN: convolutional neural network
EVC: early visual cortex
FWHM: full-width-half-maximum
GLM: general linear model
HRF: hemodynamics response function
ISI: inter-stimulus interval
ITI: inter-trial interval
LOC: lateral occipital complex
LOC: lateral occipital cortex
RDM: representational dissimilarity matrices
ROI: regions of interest
RSA: representational similarity analysis
SNR: signal-to-noise ratio
TGA: temporal generalization analysis
TGA: time-generalization analysis
TGM: temporal generalization matrix.
