# Supplementary material for: Recurrence affects the geometry of visual representations across the ventral visual stream in the human brain
Source: PLoS Biol. 2025 Aug 25;23(8):e3003354. doi: 10.1371/journal.pbio.3003354 (PMC12404645; doi:10.1371/journal.pbio.3003354)
Supplement: S1 Fig — (A, B) Temporal dynamics of object representations across categorical boundaries of naturalness (A) and animacy (B). (C, D) Pairwise object identity decoding results within (green) and across masking conditions (black), along with their differences (brown), are presented separately for the (C) late mask condition and (D) the early mask condition. Cross-classification results are sorted by training set. For (A–D), decoding chance level was 50%; significant above-chance level decoding is denoted by colored asterisks at the corresponding time points (N = 31, p < 0.05, right-tailed permutation tests, cluster definition threshold p < 0.005, cluster-threshold p < 0.05, 10,000 permutations); vertical gray line at 0 ms indicates stimulus onset; shaded margins of time courses indicate 95% confidence intervals of the decoding performance determined by bootstrapping (1,000 iterations); horizontal error bars indicate 95% confidence intervals for peak latencies. (DOCX) [file pbio.3003354.s001.docx]

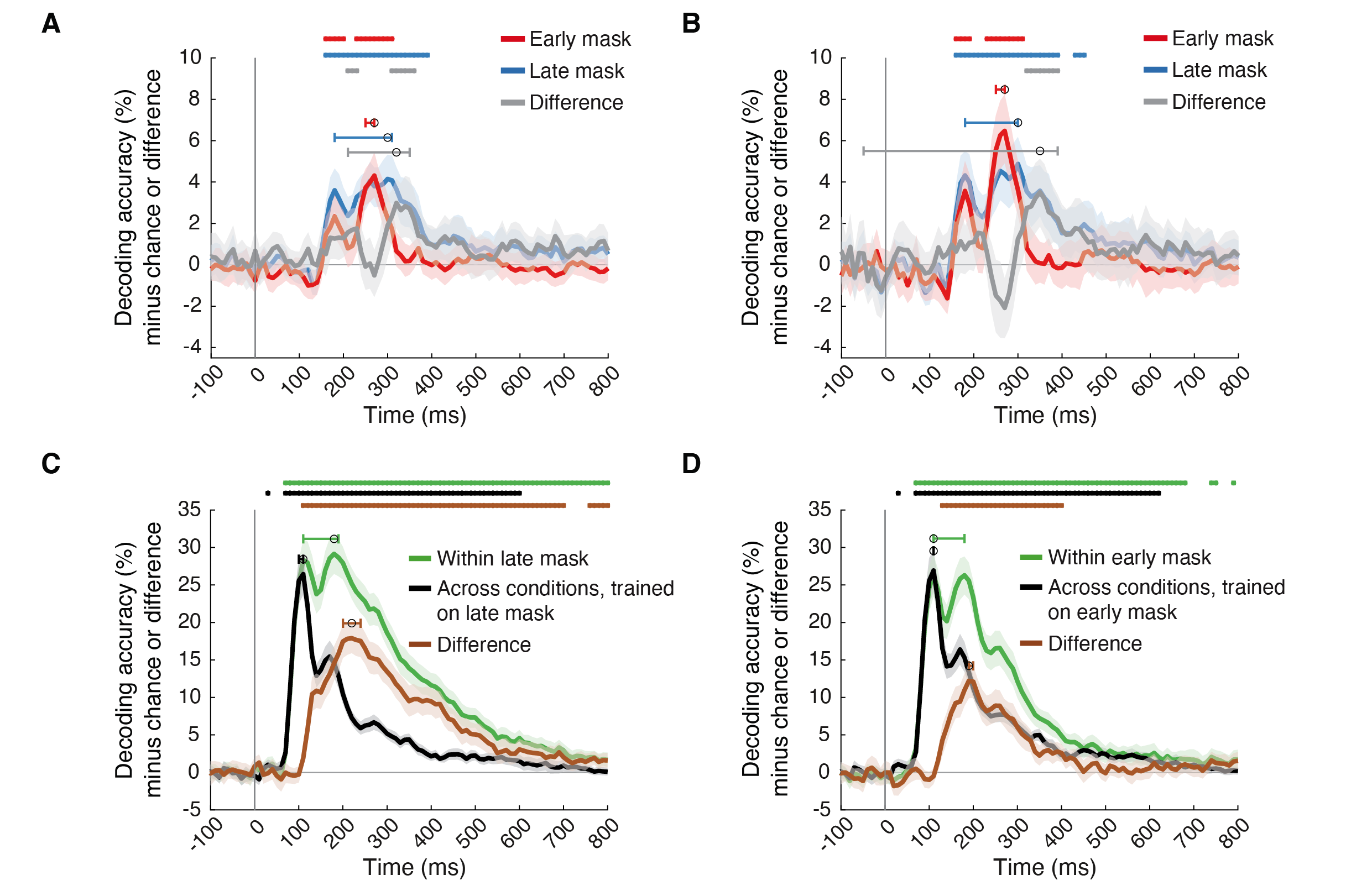


### S1 Fig. Temporal dynamics of visual object representations for the two masking conditions.

**(A, B)** Temporal dynamics of object representations across categorical boundaries of naturalness **(A)** and animacy **(B)**. **(C, D)** Pairwise object identity decoding results within (green) and across masking conditions (black), along with their differences (brown), presented separately for the **(C)** late mask condition and **(D)** the early mask condition. Cross-classification results are sorted by training set. For **(A-D)**, decoding chance level was 50%; significant above-chance level decoding is denoted by colored asterisks at the corresponding time points (N = 31, p < 0.05, right-tailed permutation tests, cluster definition threshold p < 0.005, cluster-threshold p < 0.05, 10,000 permutations); vertical gray line at 0ms indicates stimulus onset; shaded margins of time courses indicate 95% confidence intervals of the decoding performance determined by bootstrapping (1,000 iterations); horizontal error bars indicate 95% confidence intervals for peak latencies.
