# Supplementary material for: Recurrence affects the geometry of visual representations across the ventral visual stream in the human brain
Source: PLoS Biol. 2025 Aug 25;23(8):e3003354. doi: 10.1371/journal.pbio.3003354 (PMC12404645; doi:10.1371/journal.pbio.3003354)
Supplement: S2 Fig — (A) Results of time-generalized object identity decoding in the early mask condition, as shown in Fig 1E. Blue and red dots indicate an example pair of corresponding time points for negative off-diagonal decoding accuracies. (B) Hypothetical oscillatory mechanism underlying negative off-diagonal decoding. Two identical, time-locked responses for object A and B differ in phase (black solid curve and orange dashed curves). For illustration, the phase shift is set to half a cycle (π), corresponding to the time difference between two off-diagonal time points identified in (A) (blue and red circles). At time point 0π, signal A > signal B, whereas at π, signal B > signal A, illustrating a reversal in relative amplitude. A classifier trained at 0π may thus predict object identity at π with opposite labeling, resulting in below-chance classification accuracy. (DOCX) [file pbio.3003354.s002.docx]

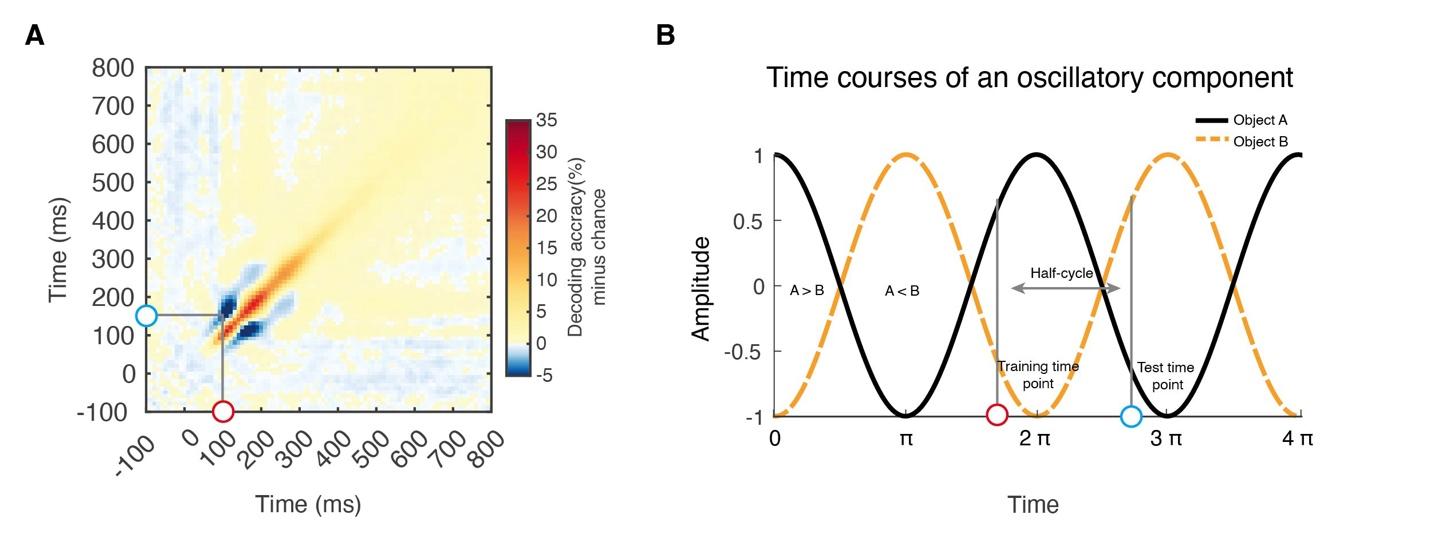


### S2 Fig. Potential neural source of negative off-diagonal decoding accuracies in temporal generalization analysis.

**(A)** Results of time-generalized object identity decoding in the early mask condition, as shown in Fig. 1E. Blue and red dots indicate an example pair of corresponding time points for negative off-diagonal decoding accuracies. **(B)** Hypothetical oscillatory mechanism underlying negative off-diagonal decoding. Two identical, time-locked responses for object A and B differ in phase (black solid curve and orange dashed curves). For illustration, the phase shift is set to half a cycle (π), corresponding to the time difference between two off-diagonal time points identified in (A) (blue and red circles). At time point 0π, signal A > signal B, whereas at π, signal B > signal A, illustrating a reversal in relative amplitude. A classifier trained at 0π may thus predict object identity at π with opposite labeling, resulting in below-chance classification accuracy.
