# Supplementary material for: Recurrence affects the geometry of visual representations across the ventral visual stream in the human brain
Source: PLoS Biol. 2025 Aug 25;23(8):e3003354. doi: 10.1371/journal.pbio.3003354 (PMC12404645; doi:10.1371/journal.pbio.3003354)
Supplement: S3 Fig — (A) Results of time-generalized object identity decoding within the late mask condition (same as Fig 1F). (B) Cross-decoding object identity using a classifier trained on the late mask condition. (C) The differences between (A) and (B). The difference plot reveals positive decoding results in the off-diagonal areas (as shown in the square rectangle in Fig 1G). This occurs due to higher decoding accuracies in the within-condition decoding (late mask) than the across-condition decoding (trained on late mask). This confirms the main results pattern: in the within late mask condition decoding (A), the negative off-diagonal decoding results are veiled by recurrent processes. In the across-conditions decoding (B), results are intermediate between the late mask and early mask results. Subtracting the former from the latter results in positive off-diagonal decoding accuracies. (D) Results of temporal generalization analysis decoding object identity within the early mask condition (same as Fig 1E). (E) Cross-decoding object identity using a classifier trained on the early mask condition. (F) The difference between (D) and (E). The difference plot reveals an opposite pattern to the main analysis result (Fig 1G and (C)), with negative decoding results in the off-diagonal areas (as shown in the square rectangle as in Fig 1G). This occurs due to lower decoding accuracies in the within-condition decoding (early mask) than across-condition decoding (trained on early mask). This also confirms the main results pattern: in the within early mask condition decoding (D), negative off-diagonal decoding results are not veiled by recurrent processes. In the across-conditions decoding (E), results are intermediate between the late mask and early mask results. Subtracting the former from the latter results in negative off-diagonal decoding accuracies. For (A–F), chance level is 50%. Time point combinations with significantly above-chance level decoding are outlined in black dashed lines (N = [file pbio.3003354.s003.docx]

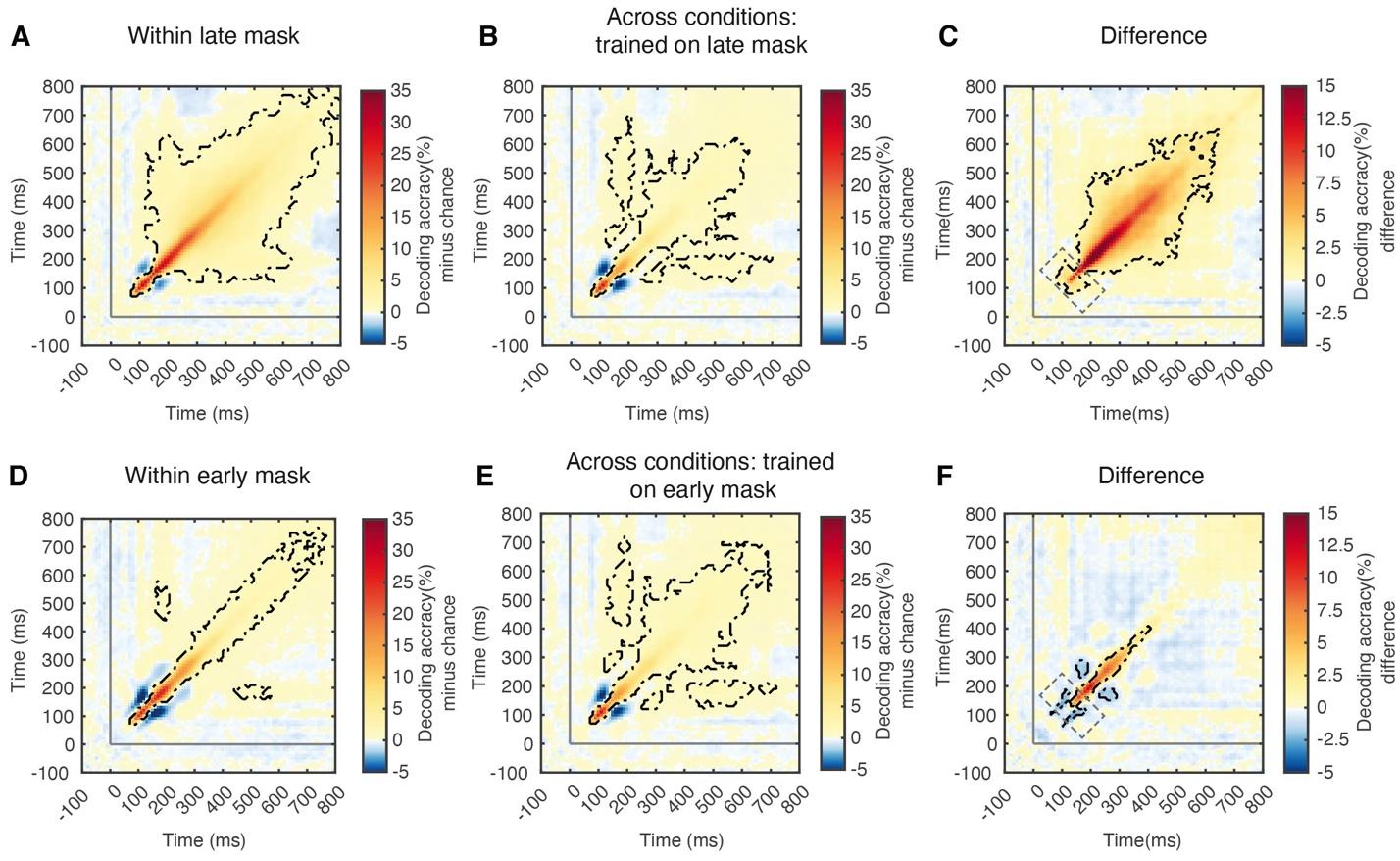


### S3 Fig. Results of temporal generalization analysis decoding object identity within- and across-conditions.

**(A)** Results of time-generalized object identity decoding within the late mask condition (same as Fig. 1F). **(B)** Cross-decoding object identity using a classifier trained on the late mask condition. **(C)** The differences between **(A)** and **(B)**. The difference plot reveals positive decoding results in the off-diagonal areas (as shown in the square rectangle in Fig. 1G). This occurs due to higher decoding accuracies in the within-condition decoding (late mask) than the across-conditions decoding (trained on late mask). This confirms the main results pattern: in the within late mask condition decoding **(A)**, the negative off-diagonal decoding results are veiled by recurrent processes. In the across-conditions decoding **(B)**, results are intermediate between the late mask and early mask results. Subtracting the former from the latter results in positive off diagonal decoding accuracies. **(D)** Results of temporal generalization analysis decoding object identity within the early mask condition (same as Fig. 1E). **(E)** Cross-decoding object identity using a classifier trained on the early mask condition. **(F**) The difference between **(D)** and **(E)**. The difference plot reveals an opposite pattern to the main analysis result (Fig. 1G and **(C)**), with negative decoding results in the off-diagonal areas (as shown in the square rectangle as in Fig. 1G). This occurs due to lower decoding accuracies in the within-condition decoding (early mask) than across-conditions decoding (trained on early mask). This also confirms the main results pattern: in the within early mask condition decoding **(D)**, negative off-diagonal decoding results are not veiled by recurrent processes. In the across-conditions decoding **(E)**, results are intermediate between the late mask and early mask results. Subtracting the former from the latter results in negative off diagonal decoding accuracies. For **(A-F)**, chance level is 50%. Time-point combinations with significantly above-chance level decoding are outlined in black dash lines (N = 31, right-tailed permutation tests, cluster definition threshold p < 0.005, cluster-threshold p < 0.05, 10,000 permutations); vertical and horizontal gray lines indicate stimulus onsets.
