# Supplementary material for: Recurrence affects the geometry of visual representations across the ventral visual stream in the human brain
Source: PLoS Biol. 2025 Aug 25;23(8):e3003354. doi: 10.1371/journal.pbio.3003354 (PMC12404645; doi:10.1371/journal.pbio.3003354)
Supplement: S4 Fig — (A) Results of object identity decoding in the late mask condition, across-conditions decoding while training on the late mask condition, and the difference between them. (B) Results of object identity decoding in the early mask conditions, across-conditions decoding while training on the early mask condition, and the difference between them. (A) and (B) show a qualitatively equivalent results pattern emerges as in Fig 2C. For (A, B), chance level is 50%; significant above-chance level decoding is denoted by black asterisks above the bars (N = 27, p < 0.05, right-tailed permutation tests, FDR-corrected); error bars indicate standard errors of the mean. (DOCX) [file pbio.3003354.s004.docx]

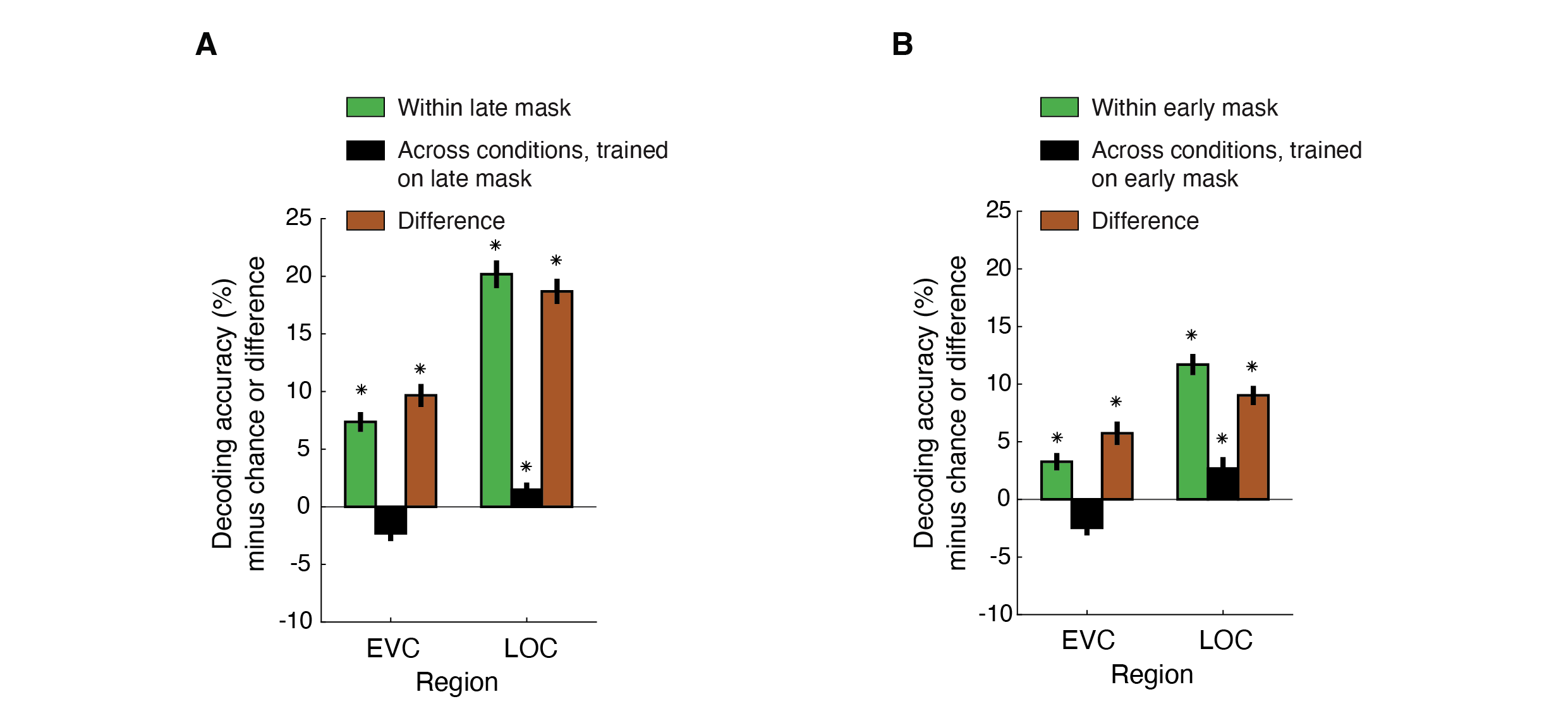


### S4 Fig. Results of visual object decoding in fMRI within and across masking conditions.

**(A)** Results of object identity decoding in the late mask condition, across conditions decoding while training on the late mask condition, and the difference between them. **(B)** Results of object identity decoding in the early mask conditions, across conditions decoding while training on the early mask condition, and the difference between them. **(A)** and **(B)** show a qualitatively equivalent results pattern emerges as in Fig. 2C. For **(A, B)**, chance level is 50%; significant above-chance level decoding is denoted by black asterisks above the bars (N = 27, p < 0.05, right-tailed permutation tests, FDR-corrected); error bars indicate standard errors of the mean.
