# Supplementary material for: Recurrence affects the geometry of visual representations across the ventral visual stream in the human brain
Source: PLoS Biol. 2025 Aug 25;23(8):e3003354. doi: 10.1371/journal.pbio.3003354 (PMC12404645; doi:10.1371/journal.pbio.3003354)
Supplement: S5 Fig — The analysis rationale is consistent with that used for Figs 1D and 2C. We compared the within-conditions decoding results (averaged across within-early-mask and within-late-mask decoding) to the across-conditions decoding results (averaged across both training and testing directions for cross-decoding). This comparison further determines the direct impact of recurrent activity on visual object representations. (A, B) Result of RSA linking object representations in (A) EVC and (B) LOC to a CNN model trained on object categorization (i.e., AlexNet) as revealed by within-condition decoding, across-conditions decoding, and the difference between them. We observe an equivalent result pattern to the main analysis reported in Fig 3B and 3C. Significant correlations are marked by black asterisks above bars (N = 27, p < 0.05, right-tailed permutation tests, FDR-corrected); error bars depict standard errors of the mean; shaded gray areas indicate the noise ceiling. (C–E) RSA results linking the CNN to EEG for the (C) within-condition decoding analysis, (D) the across-condition analysis, and (E) the difference. We observe an equivalent result pattern to the main analysis reported in Fig 3D–3F (for statistical details, see S5 Table). Significant correlations at time points are denoted by asterisks colored by layer (N = 31, right-tailed permutation tests, cluster definition threshold p < 0.005, cluster-threshold p < 0.05, 10,000 permutations); horizontal error bars indicate 95% confidence intervals for peak latencies, shaded gray areas represented the noise ceiling. (DOCX) [file pbio.3003354.s005.docx]

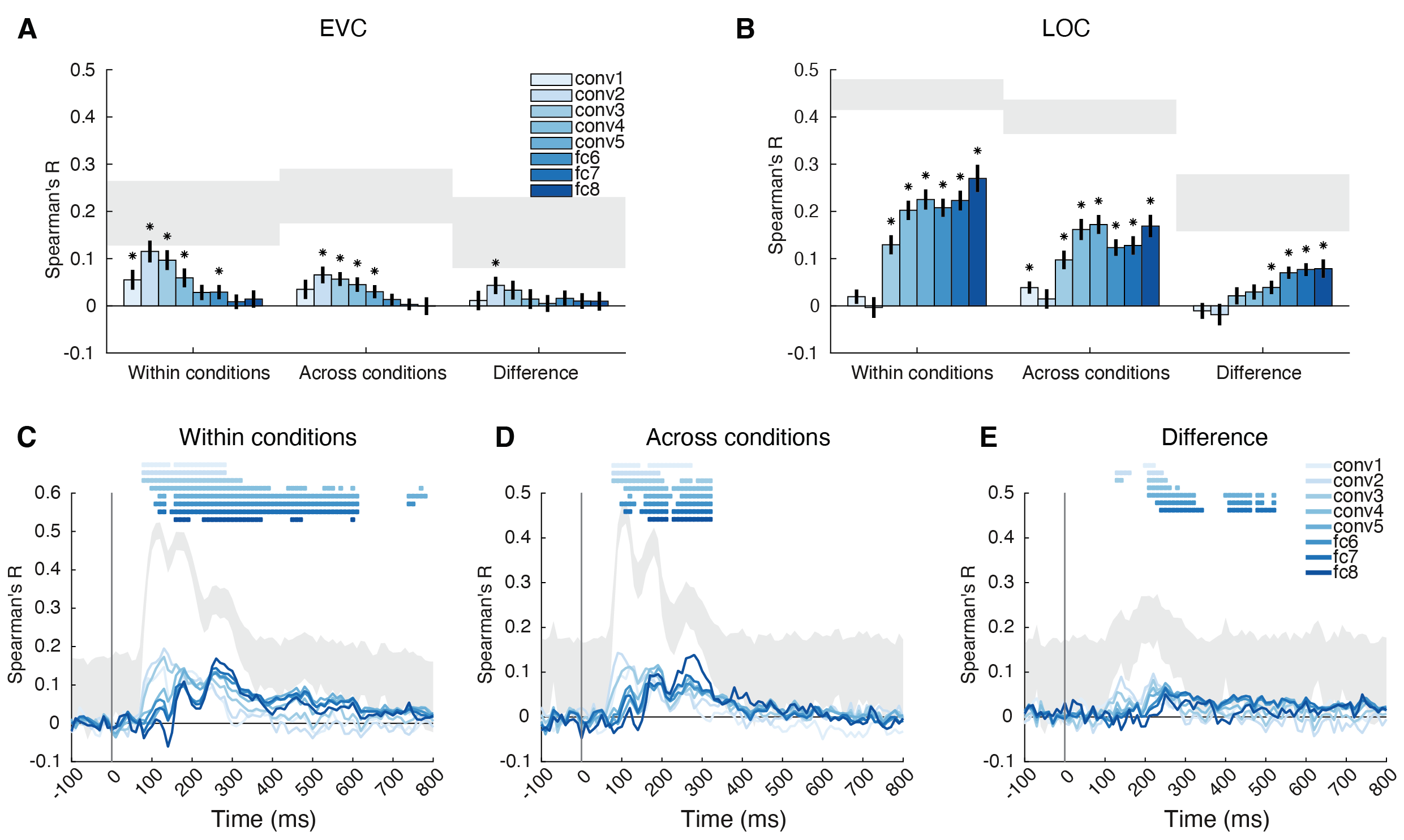


### S5 Fig. Visual features encoded in neural object representations revealed by within- and across-conditions decoding.

The analysis rationale is consistent with that used for Fig. 1D and Fig. 2C. We compared the within-conditions decoding results (averaged across within-early-mask and within-late-mask decoding) to the across-conditions decoding results (averaged across both training and testing directions for cross-decoding). This comparison further determines the direct impact of recurrent activity on visual object representations. **(A, B)** Result of RSA linking object representations in **(A)** EVC and **(B)** LOC to a CNN model trained on object categorization (i.e., AlexNet) as revealed by within condition decoding, across-conditions decoding, and the difference between them. We observe an equivalent result pattern to the main analysis reported in Fig. 3B and 3C. Significant correlations are marked by black asterisks above bars (N = 27, p<0.05, right-tailed permutation tests, FDR corrected); error bars depict standard errors of the mean; shaded gray areas indicate the noise ceiling. **(C-E)** RSA results linking the CNN to EEG for the **(C)** within condition decoding analysis, **(D)** the across condition analysis, and **(E)** the difference. We observe an equivalent result pattern to the main analysis reported in Fig. 3D-F (for statistical details, see Supplementary Table 5). Significant correlations at time points are denoted by asterisks colored by layer (N = 31, right-tailed permutation tests, cluster definition threshold p < 0.005, cluster-threshold p < 0.05, 10,000 permutations); horizontal error bars indicate 95% confidence intervals for peak latencies, shaded gray areas represented the noise ceiling.
