# Supplementary material for: Recurrence affects the geometry of visual representations across the ventral visual stream in the human brain
Source: PLoS Biol. 2025 Aug 25;23(8):e3003354. doi: 10.1371/journal.pbio.3003354 (PMC12404645; doi:10.1371/journal.pbio.3003354)
Supplement: S6 Fig — We obtained RDMs from the layers of a CNN model (here ResNet50, we used the last layer of each of the four residual blocks and the final classification layer), each ROI in fMRI, and each time point in EEG. We then calculated the correlation coefficients between the CNN layer RDMs and the EEG or fMRI RDMs. (A, B) RSA results linking (A) EVC and (B) LOC to layers of ResNet50. In EVC, the differences between masking conditions were not significant. However, in LOC, the differences between masking conditions revealed a shift in correspondences to deeper layer, i.e., the fc layer, with 95% confidence intervals of (block2, fc). For (A, B), significant correlations are marked by black asterisks above bars (N = 27, p < 0.05, right-tailed permutation tests, FDR-corrected); error bars depict standard errors of the mean; shaded gray areas indicate the noise ceiling. (C–E) RSA results linking layers of ResNet50 to EEG in the (C) early mask condition, (D) late mask condition, and (E) the difference between them. For (D–F), significant correlations at time points are denoted by asterisks colored by layer (N = 31, right-tailed permutation tests, cluster definition threshold p < 0.005, cluster-threshold p < 0.05, 10,000 permutations); horizontal error bars indicate 95% confidence intervals for peak latencies, shaded gray areas represented the noise ceiling. (DOCX) [file pbio.3003354.s006.docx]

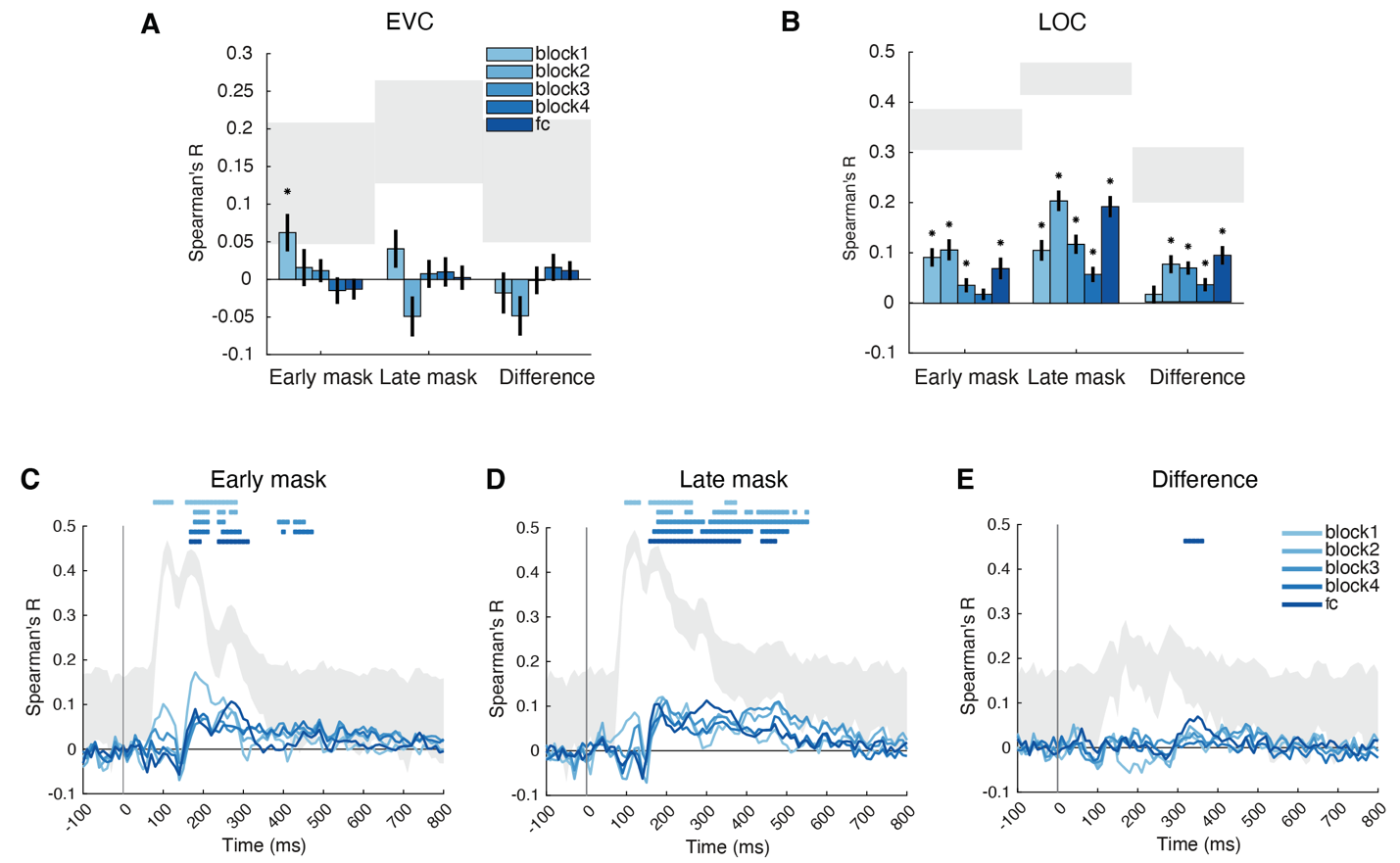


### S6 Fig. The representational format of visual representations resolved in space or time as assessed with ResNet50.

We obtained RDMs from the layers of a CNN model (here ResNet50, we used the last layer of each of the four residual blocks and the final classification layer), each ROI in fMRI, and each time point in EEG. We then calculated the correlation coefficients between the CNN layer RDMs and the EEG or fMRI RDMs. **(A, B)** RSA results linking **(A)** EVC and **(B)** LOC to layers of ResNet50. In EVC, the differences between masking conditions were not significant. However, in LOC, the differences between masking conditions revealed a shift in correspondences to deeper layer, i.e., the fc layer, with 95% confidence intervals of (block2, fc). For **(A, B)**, significant correlations are marked by black asterisks above bars (N = 27, p<0.05, right-tailed permutation tests, FDR corrected); error bars depict standard errors of the mean; shaded gray areas indicate the noise ceiling. **(C-E)** RSA results linking layers of ResNet50 to EEG in the **(C)** early mask condition, **(D)** late mask condition, and **(E)** the difference between them. For **(D-F)**, significant correlations at time points are denoted by asterisks colored by layer (N = 31, right-tailed permutation tests, cluster definition threshold p < 0.005, cluster-threshold p < 0.05, 10,000 permutations); horizontal error bars indicate 95% confidence intervals for peak latencies, shaded gray areas represented the noise ceiling.
