# Supplementary material for: Recurrence affects the geometry of visual representations across the ventral visual stream in the human brain
Source: PLoS Biol. 2025 Aug 25;23(8):e3003354. doi: 10.1371/journal.pbio.3003354 (PMC12404645; doi:10.1371/journal.pbio.3003354)
Supplement: S7 Fig — The analysis rationale is consistent with that used for Figs 1D and 2C. We compared the within-condition decoding results (averaged across within-early-mask and within-late-mask decoding) to the across-condition decoding results (averaged across both training and testing directions for cross-decoding). This comparison helped determine the direct impact of recurrent activity on visual object representations. (A–F) Results of time- and frequency-resolved object identity decoding within-conditions, across-conditions, and their differences. The decoding analyses were based on frequency power values in (A–C) and phase values in (D–F). For (A–F), chance level was 50%; time-frequency combinations with significant above-chance decoding are outlined by black dash lines (N = 31, right-tailed permutation tests, cluster definition p < 0.05, significance p < 0.05, 10,000 permutations); the vertical gray line indicates stimulus onset. (DOCX) [file pbio.3003354.s007.docx]

**
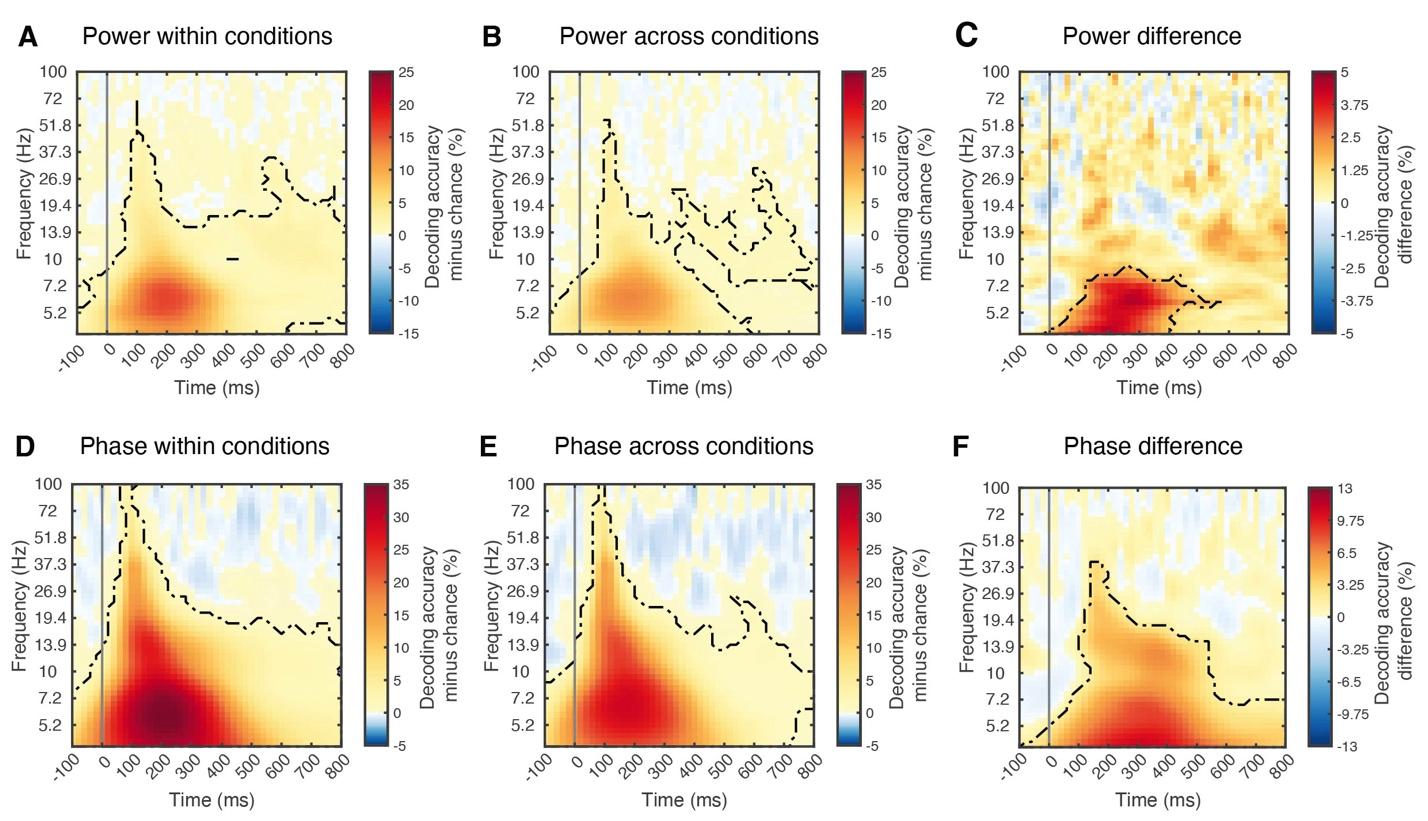
**

### S7 Fig. Spectral characteristics of visual representations as revealed by within and across masking conditions decoding.

The analysis rationale is consistent with that used for Fig. 1D and Fig. 2C. We compared the within-condition decoding results (averaged across within-early-mask and within-late-mask decoding) to the across-condition decoding results (averaged across both training and testing directions for cross-decoding). This comparison helped determine the direct impact of recurrent activity on visual object representations. **(A-F)** Results of time- and frequency-resolved object identity decoding within conditions, across conditions, and their differences. The decoding analyses were based on frequency power values in **(A-C)** and phase values in **(D-F)**. For **(A-F)**, chance level was 50%; time-frequency combinations with significant above-chance decoding are outlined by black dash lines (N = 31, right-tailed permutation tests, cluster definition p < 0.05, significance p < 0.05, 10,000 permutations); the vertical gray line indicates stimulus onset.
