# Supplementary material for: Recurrence affects the geometry of visual representations across the ventral visual stream in the human brain
Source: PLoS Biol. 2025 Aug 25;23(8):e3003354. doi: 10.1371/journal.pbio.3003354 (PMC12404645; doi:10.1371/journal.pbio.3003354)
Supplement: S8 Fig — (A) To investigate where in the brain the specific visual features originate and how each of the four spectro-temporally identified components carries them, we conducted commonality analysis based on RSA linking identified time-frequency resolved dynamics (EEG), cortical locus (fMRI), and feature complexity (CNN layers of AlexNet). We calculated coefficients of shared variance among frequency-based EEG RDMs corresponding to each spectro-temporally identified component, fMRI RDMs within each ROI, and CNN RDMs across each layer. This analysis yielded coefficients of shared variance for each of the four identified components and for each CNN layer in EVC and LOC, respectively. (B–C) Shared variance for the two identified power components across brain regions and CNN layers. (D–E) Shared variance for the two identified phase components across brain regions and CNN layers. We observed three result patterns, all common across the four components and reinforcing together the outcome of the main analyses. First, we observed significant relationships to CNN layers for all components, regions, and both masking conditions (except for EVC and the alpha–beta power component in the early mask condition), demonstrating the analytical feasibility of the approach. Second, the shared variance was generally lower in the early mask condition compared to the late mask condition, as reported for all main analyses. Third, in relation to EVC the components encompassed representations in low- to mid-level visual feature format as indexed by highest correlations to low and mid CNN layers, whereas in LOC the components encompassed representations in mid to high level visual feature format as indexed by highest correlations to mid and high CNN layers. Significant effects at individual CNN layers are marked with asterisks (N = 31, right-tailed permutation tests, FDR-corrected p < 0.05, 10,000 permutations). “Diff.” denotes late mask minus early mask. (DOCX) [file pbio.3003354.s008.docx]

**
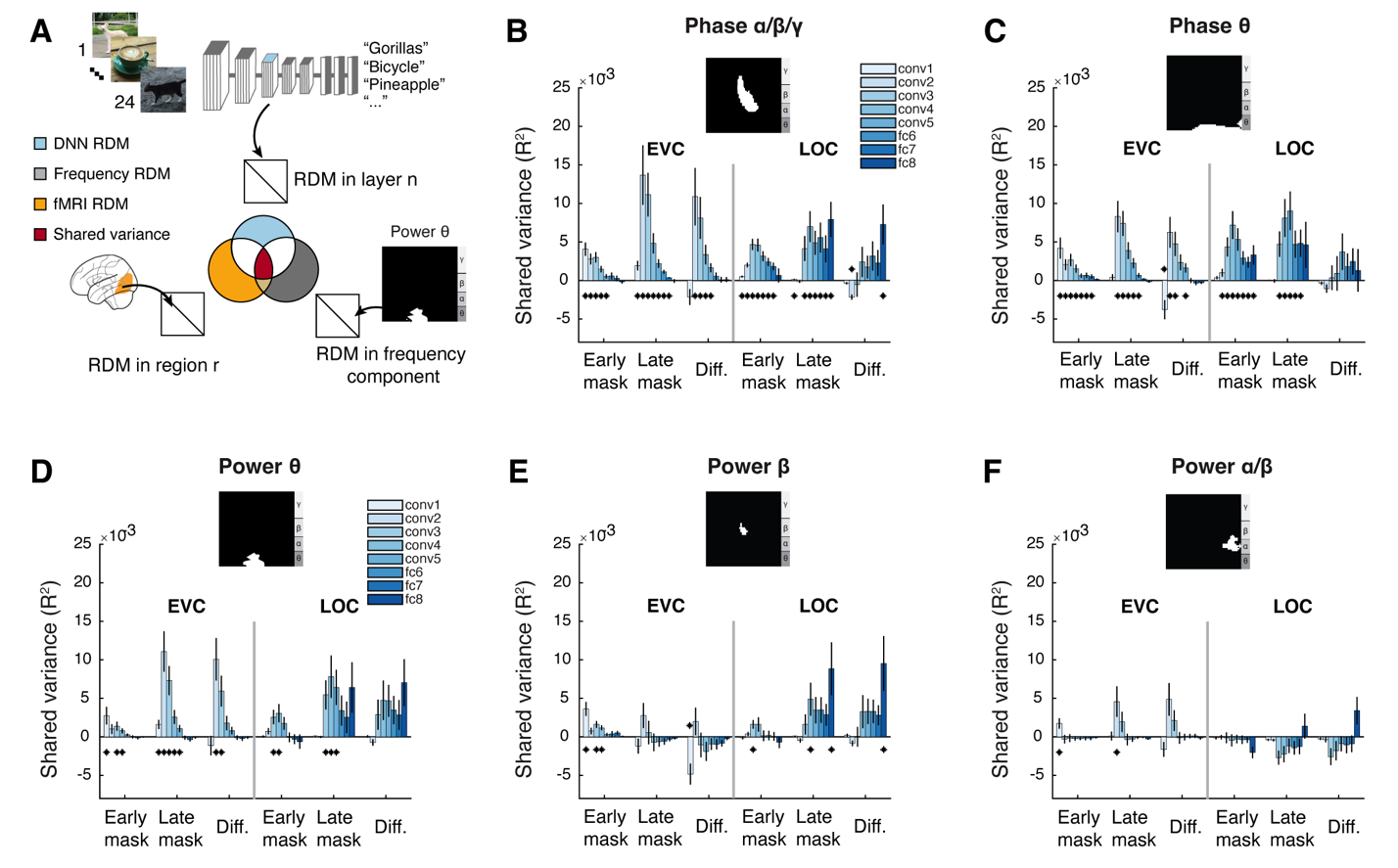
**

### S8 Fig. Feature format and cortical origin of the spectral components underlying recurrent processing.

**(A)** To investigate where in the brain the specific visual features originate and how each of the four spectro-temporally identified components carries them, we conducted commonality analysis based on RSA linking identified time-frequency resolved dynamics (EEG), cortical locus (fMRI) and feature complexity (CNN layers of AlexNet). We calculated coefficients of shared variance among frequency-based EEG RDMs corresponding to each spectro-temporally identified component, fMRI RDMs within each ROI, and CNN RDMs across each layer. This analysis yielded coefficients of shared variance for each of the four identified components and for each CNN layer in EVC and LOC respectively. **(B-C)** Shared variance for the two identified power components across brain regions and CNN layers. **(D-E)** Shared variance for the two identified phase components across brain regions and CNN layers. We observed three result patterns, all common across the four components and reinforcing together the outcome of the main analyses. First*,* we observed significant relationships to CNN layers for all components, regions, and both masking conditions (except for EVC and the alpha-beta power component in the early mask condition), demonstrating the analytical feasibility of the approach. Second, the shared variance was generally lower in the early mask condition compared to the late mask condition, as reported for all main analyses. Third, in relation to EVC the components encompassed representations in low- to mid- level visual feature format as indexed by highest correlations to low and mid CNN layers, whereas in LOC the components encompassed representations in mid to high level visual feature format as indexed by highest correlations to mid and high CNN layers. Significant effects at individual CNN layers are marked with asterisks (N = 31, right-tailed permutation tests, FDR corrected p < 0.05, 10,000 permutations). “Diff.” denotes late-mask minus early-mask.
