# Supplementary material for: Recurrence affects the geometry of visual representations across the ventral visual stream in the human brain
Source: PLoS Biol. 2025 Aug 25;23(8):e3003354. doi: 10.1371/journal.pbio.3003354 (PMC12404645; doi:10.1371/journal.pbio.3003354)
Supplement: S1 Table — (DOCX) [file pbio.3003354.s009.docx]

## Supplementary Table

### S1 Table. Statistical details for object identity decoding using EEG signals.

| **Type of decoding** | **Peak value*** | **Peak latency (95% CI) #** | **Significant time points+** |
| --- | --- | --- | --- |
| Within-condition (early mask) | 26.42% | 110ms (110, 180) | [70:680, 740:750, 790:800] |
| Within-condition (late mask) | 29.14% | 180ms (110, 190) | [70:800] |
| Difference (within late minus within early mask) | 7.89% | 230ms (220, 420) | [110:560] |
| Across-conditions (averaged across both training and testing directions) | 26.69% | 110ms (100, 110) | [30, 70:600, 620] |
| Difference (within-conditions minus across-conditions decoding) | 14.78% | 200ms (200, 210) | [120:540, 570, 590:650, 680:690, 730:800] |

* Decoding accuracy (%) minus chance level (50%)

# The unit of time was milliseconds and the 95% confidence intervals added in parentheses were calculated by bootstrapping participants (n = 1,000)

+ Right-tailed cluster-based permutation tests, cluster definition p < 0.005, significance p < 0.05
