# Supplementary material for: Recurrence affects the geometry of visual representations across the ventral visual stream in the human brain
Source: PLoS Biol. 2025 Aug 25;23(8):e3003354. doi: 10.1371/journal.pbio.3003354 (PMC12404645; doi:10.1371/journal.pbio.3003354)
Supplement: S2 Table — (DOCX) [file pbio.3003354.s010.docx]

### S2 Table. Statistical details for object naturalness and animacy decoding using EEG signals.

| **Type of decoding** | **Peak value*** | **Peak latency (95% CI) #** | **Significant time points+** |
| --- | --- | --- | --- |
| 1. **Naturalness** | | | |
| Within-condition (early mask) | 4.31% | 270ms (250, 270) | [160:200, 230:310] |
| Within-condition (late mask) | 4.15% | 300ms (180, 310) | [160:390] |
| Difference (within late minus within early mask) | 2.99% | 320ms (210, 350) | [210:230, 310:360] |
| 1. **Animacy** | | | |
| Within-condition (early mask) | 6.47% | 270ms (250, 270) | [160:190, 230:310] |
| Within-condition (late mask) | 4.87% | 300ms (180, 300) | [160:390, 430:450] |
| Difference (within late minus within early mask) | 3.50% | 350ms (-50, 390) | [320:390] |
| 1. **Trained on late mask condition** | | | |
| Across-condition (trained on late mask) | 26.45% | 110ms (100, 110) | [30, 70:600] |
| Difference (within late mask minus across-condition trained on late mask) | 17.90% | 220ms (200, 240) | [-180, 110:700, 760:800] |
| 1. **Trained on early mask condition** | | | |
| Across-condition (trained on early mask) | 26.93% | 110ms (110, 110) | [30, 70:620] |
| Difference (within early mask decoding minus across-conditions trained on early mask) | 12.21% | 190ms (190, 200) | [130:400] |

* Decoding accuracy (%) minus chance level (50%)

# The unit of time was milliseconds and the 95% confidence intervals added in parentheses were calculated by bootstrapping participants (n = 1,000)

+ Right-tailed cluster-based permutation tests, cluster definition p < 0.005, significance p < 0.05
