# Supplementary material for: Recurrence affects the geometry of visual representations across the ventral visual stream in the human brain
Source: PLoS Biol. 2025 Aug 25;23(8):e3003354. doi: 10.1371/journal.pbio.3003354 (PMC12404645; doi:10.1371/journal.pbio.3003354)
Supplement: S3 Table — (DOCX) [file pbio.3003354.s011.docx]

### S3 Table. Statistical details for peak locations and spatial extent of top 3 clusters in fMRI searchlight analysis.

| **Type of decoding** | **Peak [X, Y, Z] mm** | **Peak Value*** | **Volume voxel number** | **Assignment based on Maximum Probability Map and Percent of Area activated by Cluster** |
| --- | --- | --- | --- | --- |
| 1. **Within-condition (early mask)** | | | | |
| Cluster 1 | [-46, -74, -8] | 8.7% | 5914 | hOc4lp 40.2%; hOc4la 36.0%; FG2 53.9%; FG4 31.7%; hOc4v [V4(v)] 23.9%; hOc3v [V3v] 17.5%; FG1 35.9%; hIP4 (IPS) 35.5%; FG3 18.4%; hOc4d [V3A] 17.6%; hIP7 (IPS) 23.9%; hOc5 [V5/MT] 47.7%; PGp (IPL) 5.4%; hOc3d [V3d] 6.2%; hOc2 [V2] 2.6%; hIP5 (IPS) 3.4%; hOc1 [V1] 0.7%; PFm (IPL) 0.7%; hPO1 (IPS) 0.6%; PGa (IPL) 0.3% |
| Cluster 2 | [56, -70, -6] | 6.9% | 3155 | hOc4la 38.2%; FG4 25.3%; FG2 39.0%; hOc4v [V4(v)] 13.6%; FG1 32.3%; hOc4lp 11.7%; FG3 12.7%; hIP4 (IPS) 20.6%; hIP7 (IPS) 12.2%; hOc5 [V5/MT] 27.0%; hIP5 (IPS) 6.1%; hOc3v [V3v] 1.9%; PGp (IPL) 1.5%; hOc3d [V3d] 1.0%; hOc4d [V3A] 1.5%; hIP8 (IPS) 0.1% |
| Cluster 3 | [18, -96, -2] | 3.5% | 396 | hOc1 [V1] 6.8%; hOc2 [V2] 4.4%; hOc3v [V3v] 1.5% |
| 1. **Within-condition (late mask)** | | | | |
| Cluster 1 | [-44, -74, -8] | 14.0% | 22,818 | hOc3v [V3v] 77.1%; hOc4v [V4(v)] 91.1%; hOc4lp 87.5%; hOc1 [V1] 33.0%; FG4 73.6%; hOc2 [V2] 35.7%; FG3 87.8%; FG2 99.6%; FG1 96.0%; hIP4 (IPS) 97.7%; hOc3d [V3d] 32.7%; hIP7 (IPS) 86.6%; hOc4d [V3A] 45.3%; PGp (IPL) 23.5%; hIP5 (IPS) 38.8%; hOc5 [V5/MT] 100.0%; CA1 (Hippocampus) 11.8%; hPO1 (IPS) 11.9%; hIP8 (IPS) 6.8%; Subiculum 4.5%; PGa (IPL) 1.6%; 7M (SPL) 8.6%; TE 4 1.0%; DG (Hippocampus) 2.3%; TE 5 0.5%; TE 1.0 1.8%; hOc6 [V6] 1.6%; PFcm (IPL) 0.6%; PFm (IPL) 0.2%; hIP6 (IPS) 0.4%; TE 3 0.2%; Amygdala (VTM) 1.3% |
| Cluster 2 | [-32, -2, -50] | 3.6% | 196 | Entorhinal Cortex 0.1% |
| Cluster 3 | [40, 28, -12] | 3.4% | 135 | OP9 1.2%; Fo7 0.4%; Fo6 0.1% |
| 1. **Difference (within late minus within early mask)** | | | | |
| Cluster 1 | [-42, -80, -12] | 7.2% | 2456 | hOc4la 31.2%; hOc4lp 33.9%; FG2 39.8%; hOc4v [V4(v)] 13.3%; hOc1 [V1] 3.5%; FG3 10.0%; FG4 6.5%; hOc2 [V2] 2.7%; hOc5 [V5/MT] 26.6%; hOc3v [V3v] 3.8%; FG1 8.8%; hOc3d [V3d] 2.5%; hIP4 (IPS) 2.0%; PGp (IPL) 0.3%; hOc4d [V3A] 0.1% |
| Cluster 2 | [54, -74, -2] | 7.4% | 1771 | hOc4la 36.4%; hOc4lp 29.7%; FG2 24.5%; hOc4v [V4(v)] 12.5%; hOc3v [V3v] 5.6%; hIP4 (IPS) 15.5%; FG4 4.3%; FG1 7.7%; PGp (IPL) 1.4%; hOc5 [V5/MT] 9.8%; hOc2 [V2] 0.7%; hIP7 (IPS) 3.0%; hOc3d [V3d] 0.2%; hOc4d [V3A] 0.1% |
| Cluster 3 | [24, -50, -10] | 4.5% | 61 | FG3 0.9%; FG1 0.5%; hOc3v [V3v] 0.1%; hOc4v [V4(v)] 0.1% |

* Decoding accuracy (%) minus chance level (50%)
