# Supplementary material for: Recurrence affects the geometry of visual representations across the ventral visual stream in the human brain
Source: PLoS Biol. 2025 Aug 25;23(8):e3003354. doi: 10.1371/journal.pbio.3003354 (PMC12404645; doi:10.1371/journal.pbio.3003354)
Supplement: S4 Table — (DOCX) [file pbio.3003354.s012.docx]

### S4 Table. Statistical details for the RSA results linking the AlexNet model to EEG decoding RDMs within the early and late mask conditions, and the difference between these conditions.

| **Condition**  **Layer group** | **Early mask** | | | **Late mask** | | | **Difference (late mask minus early mask)** | | |
| --- | --- | --- | --- | --- | --- | --- | --- | --- | --- |
|  | Peak value* | Peak latency (95% CI) # | Significant time points+ | Peak value* | Peak latency (95% CI) # | Significant time points+ | Peak value* | Peak latency (95% CI) # | Significant time points+ |
| 1 | 0.13 | 200ms (100, 210) | [80:130, 160:280] | 0.14 | 130ms (90, 130) | [70:140, 160:270] | n.s. | n.s. | n.s. |
| 2 | 0.15 | 110ms (100, 140) | [80:280] | 0.18 | 130ms (100, 160) | [80:260, 280] | n.s. | n.s. | n.s. |
| 3 | 0.13 | 110ms (110, 220) | [80:320] | 0.17 | 130ms (120, 170) | [80:280] | 0.06 | 160ms (-70, 740) | [160:170] |
| 4 | 0.12 | 260ms (180, 280) | [110:320] | 0.14 | 170ms (160, 190) | [110:400, 430:500, 550:580, 600:610] | 0.08 | 470ms (-100, 610) | [160:170, 470] |
| 5 | 0.12 | 260ms (180, 280) | [160:340] | 0.13 | 180ms (170, 480) | [120:130, 160:610] | 0.07 | 470ms (-100, 480) | [170, 360, 470:480] |
| 6 | 0.12 | 260ms (250, 280) | [160:190, 220:330] | 0.10 | 170ms (170, 480) | [110:130, 150:610] | 0.07 | 350ms (140, 480) | [170, 340:380, 470:480] |
| 7 | 0.13 | 260ms (250, 280) | [160:180, 230:330] | 0.11 | 170ms (170, 480) | [120:130, 150:610] | 0.08 | 470ms (340, 480) | [170, 320:370, 440:480] |
| 8 | 0.16 | 270ms (250, 290) | [170:180, 240:310] | 0.11 | 260ms (180, 360) | [160:190, 230:380, 430:480, 600] | 0.11 | 350ms (-110, 470) | [330:360] |

* Spearman correlation coefficients

# The unit of latency was milliseconds and the 95% confidence intervals added in parentheses were calculated by bootstrapping participants (n = 1,000)

+ Right-tailed cluster-based permutation tests, cluster definition p < 0.005, significance p < 0.05
