# Supplementary material for: Recurrence affects the geometry of visual representations across the ventral visual stream in the human brain
Source: PLoS Biol. 2025 Aug 25;23(8):e3003354. doi: 10.1371/journal.pbio.3003354 (PMC12404645; doi:10.1371/journal.pbio.3003354)
Supplement: S5 Table — (DOCX) [file pbio.3003354.s013.docx]

### S5 Table. Statistical details for the RSA results linking the AlexNet model to EEG decoding RDMs within-, across-conditions, and the difference between them.

| **Condition**  **Layer** | **Within conditions** | | | **Across conditions** | | | **Within - across** | | |
| --- | --- | --- | --- | --- | --- | --- | --- | --- | --- |
|  | Peak value* | Peak latency (95% CI) # | Significant time points+ | Peak value* | Peak latency (95% CI) # | Significant time points+ | Peak value* | Peak latency (95% CI) # | Significant time points+ |
| 1 | 0.15 | 130ms (90, 210) | [80:140, 160:280] | 0.11 | 120ms (90, 190) | [80:140, 170:270] | 0.06 | 210ms (-80, 660) | [200:220] |
| 2 | 0.20 | 130ms (120, 140) | [80:280] | 0.14 | 90ms (90, 130) | [70:190] | 0.10 | 220ms (130, 230) | [130:160, 210:240] |
| 3 | 0.17 | 130ms (120, 160) | [80:320] | 0.11 | 120ms (100, 190) | [80:200. 250:270, 290:320] | 0.08 | 220ms (130, 280) | [130:140, 210:250] |
| 4 | 0.14 | 180ms (160, 280) | [100:390, 440:480, 530:550, 570, 600, 770] | 0.12 | 190ms (170, 270) | [110:210, 230:320] | 0.07 | 220ms (160, 680) | [210:260, 280] |
| 5 | 0.14 | 180ms (180, 280) | [120,130, 160:610, 740:780] | 0.11 | 190ms (170, 270) | [120, 160:220, 250:320] | 0.08 | 250ms (230, 450) | [210:320, 400:460, 480:490, 520] |
| 6 | 0.13 | 260ms (250, 290) | [110:130, 160:610, 740:750] | 0.08 | 200ms (120, 290) | [100:130, 160:210, 240:320] | 0.07 | 250ms (240, 680) | [230:320, 410:460, 480:520] |
| 7 | 0.14 | 260ms (250, 290) | [120:130, 150:610] | 0.09 | 260ms (170, 290) | [110:120, 150:210, 230:320] | 0.06 | 250ms (250, 600) | [240:340, 410:460, 480:520] |
| 8 | 0.17 | 260ms (250, 300) | [160:190, 230:370, 450:470, 600] | 0.14 | 280ms (190, 290) | [170:210, 230:320] | n.s. | n.s. | n.s. |

* Spearman correlation coefficients

# The 95% confidence intervals added in parentheses were calculated by bootstrapping participants (n = 1,000)

+ Right-tailed cluster-based permutation tests, cluster definition p < 0.005, significance p < 0.05
