# Supplementary material for: Recurrence affects the geometry of visual representations across the ventral visual stream in the human brain
Source: PLoS Biol. 2025 Aug 25;23(8):e3003354. doi: 10.1371/journal.pbio.3003354 (PMC12404645; doi:10.1371/journal.pbio.3003354)
Supplement: S6 Table — (DOCX) [file pbio.3003354.s014.docx]

### S6 Table. Statistical details for RSA-based commonality analysis results linking RDMs from EEG, fMRI and the AlexNet model.

| **Condition**  **Layer group** | **Early mask** | | | **Late mask** | | | **Difference (late mask minus early mask)** | | |
| --- | --- | --- | --- | --- | --- | --- | --- | --- | --- |
|  | Peak value* | Peak latency (95% CI) # | Significant time points+ | Peak value* | Peak latency (95% CI) # | Significant time points+ | Peak value* | Peak latency (95% CI) # | Significant time points+ |
| 1. **EVC** | | | | | | | | | |
| 1 | 0.005 | 100ms (-30, 510) | [70:130, 190:210, 490:510, 610, 750:760] | 0.007 | 130ms (90, 130) | [90:150] | n.s. | n.s. | n.s. |
| 2 | 0.008 | 120ms (100, 760) | [80:130, 190:200, 410, 450, 500, 590:610, 650:660, 680, 700:710,740:770] | 0.018 | 130ms (90, 130) | [80:130, 200:240, 620:640] | 0.011 | 130ms (80, 160) | 130 |
| 3 | 0.006 | 110ms (100, 580) | [80:130, 190:200, 410:450, 500:530, 580:600, 640:660, 680:760] | 0.014 | 130ms (100, 130) | [70:140, 200:240, 620:630] | 0.010 | 130ms (70, 170) | [110:130] |
| 4 | 0.003 | 110ms (100, 550) | [80, 100:130, 420:430, 450, 510:520, 580, 650:660, 750:760] | 0.005 | 130ms (90:420) | [90, 110:130, 210:220] | 0.003 | 130ms (10, 430) | 130 |
| 5 | 0.001 | 200ms (-30, 520) | [100, 130, 430] | 0.002 | 120ms (110, 180) | [110:130] | n.s. | n.s. | n.s. |
| 6 | n.s. | n.s. | n.s. | 0.001 | 120ms (110, 180) | [110:130] | n.s. | n.s. | n.s. |
| 7 | n.s. | n.s. | n.s. | n.s. | n.s. | n.s. | n.s. | n.s. | n.s. |
| 8 | n.s. | n.s. | n.s. | n.s. | n.s. | n.s. | n.s. | n.s. | n.s. |
| 1. **LOC** | | | | | | | | | |
| 1 | 0.002 | 180ms (180, 190) | [170:190] | n.s. | n.s. | n.s. | n.s. | n.s. | n.s. |
| 2 | 0.002 | 180ms (160, 180) | [160:190] | n.s. | n.s. | n.s. | n.s. | n.s. | n.s. |
| 3 | 0.007 | 180ms (170, 250) | [160:210, 220, 240:280] | 0.011 | 180ms (170, 190) | [160:210] | n.s. | n.s. | n.s. |
| 4 | 0.011 | 180ms (180, 280) | [160:220, 240:280] | 0.019 | 180ms (170. 190) | [160:220, 250, 340:350, 440] | 0.009 | 170ms (160, 480) | [170, 190] |
| 5 | 0.008 | 180ms (180, 280) | [170:190, 210, 240:280] | 0.018 | 180ms (180, 190) | [160:260, 320:370, 400, 430:450] | 0.011 | 190ms (170, 480) | [170:190, 330:340, 360:370, 440:450] |
| 6 | 0.005 | 270ms (180, 280) | [170:180, 250:280] | 0.011 | 170ms (170, 480) | [160:190, 220, 240:300, 320:360, 400:410, 430:450] | 0.009 | 340ms (170, 710) | [160:170, 190, 320, 340:360, 440:450] |
| 7 | 0.004 | 250ms (180, 280) | [170:180, 250:280] | 0.011 | 180ms (170, 480) | [160:190, 220, 240:300, 320:360, 400:410, 430:450] | 0.009 | 170ms (160, 710 | [160:190, 320:360, 440:450] |
| 8 | 0.008 | 270ms (180, 280) | [170:180, 240:280] | 0.017 | 340ms (170, 710) | [170:180, 240:250, 270:280, 300:380, 400, 440:450] | 0.015 | 340ms (170, 710) | [320:380] |

* Coefficients of shared variance

# The 95% confidence intervals added in parentheses were calculated by bootstrapping participants (n = 1,000)

+ Right-tailed cluster-based permutation tests, cluster definition p < 0.05, significance p < 0.05
