# Supplementary material for: Recurrence affects the geometry of visual representations across the ventral visual stream in the human brain
Source: PLoS Biol. 2025 Aug 25;23(8):e3003354. doi: 10.1371/journal.pbio.3003354 (PMC12404645; doi:10.1371/journal.pbio.3003354)
Supplement: S7 Table — (DOCX) [file pbio.3003354.s015.docx]

### S7 Table. Statistical details for object identity decoding using spectro-temporally resolved EEG signals.

| **Type of decoding** | **Peak value *** | **Peak latency (95% CI) #** | **Peak frequency (95% CI) #** | **Significant time points +** | **Significant frequency ranges +** |
| --- | --- | --- | --- | --- | --- |
| 1. **Frequency power values** | | | | | |
| Within-condition (early mask) | 15.00% | 200ms (180, 220) | 6.77hz (5.56, 7.22) | [-140:800] | [4.00:63.14] |
| Within-condition (late mask) | 17.68% | 200ms (180, 200) | 6.34hz (5.20, 6.77) | [-120:800] | [4.00:39.86] |
| Difference (late mask minus early mask) | 5.21% | 540ms (120, 580) | 10.72hz (4, 26.88) | [0:320, 420:800] | [4.00:32.73] |
| 1. **Frequency phase values** | | | | | |
| Within-condition (early mask) | 35.48% | 200ms (200, 220) | 5.93hz (5.56, 6.77) | [-300:800] | [4.00:100.00] |
| Within-condition (late mask) | 36.06% | 200ms (200, 220) | 5.56hz (4.87, 6.77) | [-300:800] | [4.00:100.00] |
| Difference (late mask minus early mask) | 8.29% | 180ms (160, 220) | 20.67hz (16.97, 23.57) | [-20:800] | [4.00:72.00] |

* Decoding accuracy (%) minus chance level (50%)

# The 95% confidence intervals added in parentheses were calculated by bootstrapping participants (n = 1,000)

+ Right-tailed cluster-based permutation tests, cluster definition p < 0.05, significance p < 0.05
