# Supplementary material for: Recurrence affects the geometry of visual representations across the ventral visual stream in the human brain
Source: PLoS Biol. 2025 Aug 25;23(8):e3003354. doi: 10.1371/journal.pbio.3003354 (PMC12404645; doi:10.1371/journal.pbio.3003354)
Supplement: S8 Table — (DOCX) [file pbio.3003354.s016.docx]

### S8 Table. Behavioral performance during EEG and fMRI experiments.

| **Measure**  **Condition** | **EEG experiment** | | | **fMRI experiment** | | |
| --- | --- | --- | --- | --- | --- | --- |
|  | Mean (S.D.) | Difference (95% CI) | P-value* | Mean (S.D.) | Difference (95% CI) | P-value* |
|  | **Correctness minus chance (%) #** | | | **d-prime** | | |
| Overall | 33.27 (6.91) | | | 3.27 (2.26) | | |
| Early mask | 29.03 (8.67) | 8.48 (6.51, 10.54) | <0.001 | 2.01 (1.01) | 2.51 (1.82, 3.32) | <0.001 |
| Late mask | 37.51 (6.11) |  |  | 4.53 (2.41) |  |  |
|  | **Reaction time (ms)** | | | **Reaction time (ms)+** | | |
| Overall | 504.56 (92.86) | | | 631.62 (154.06) | | |
| Early mask | 513.83 (110.09) | 18.23 (8.10, 30.11) | 0.002 | 652.48 (123.64) | 41.72 (-1.69, 85.37) | 0.029 |
| Late mask | 495.60 (86.73) |  |  | 610.76 (179.74) |  |  |

# Correctness was the corrected response accuracy (i.e., raw response accuracy (%) minus chance accuracy 50%) for the two alternatives-forced choices task following the presentation of the early mask or the late mask conditions in the EEG experiment

+ Participant number was 24 with 3 missing data

* P-values denoted significance level of difference by paired t-tests (N=31 in EEG experiment, N=27 in fMRI experiment) comparing the mean of the measurements taken from the same participant between early mask and late mask conditions
