# Supplementary material for: Recurrence affects the geometry of visual representations across the ventral visual stream in the human brain
Source: PLoS Biol. 2025 Aug 25;23(8):e3003354. doi: 10.1371/journal.pbio.3003354 (PMC12404645; doi:10.1371/journal.pbio.3003354)
Supplement: Attachment — Submitted filename: Response_to_Reviewers.pdf [file pbio.3003354.s017.pdf]

EDITORIAL REQUESTS

As you will see below, the reviewers you will see that the reviewers are overall interested in the study, but raise a few concerns regarding some specifics of the analyses and the conclusiveness of the work. R2 and R3 also don't think that the CNN model is needed and could be removed. We have discussed this with our Academic Editor and encourage you to remove the CNN model from the manuscript.

We thank the reviewers for the thorough evaluation of our work and the constructive comments. We acknowledge the concerns by R2 and R3 concerning the CNN model. In response we have clarified that we use the CNN as a tool to quantitatively assess a property of visual representations - the complexity of the visual features represented - and not as a model of recurrent processing. We believe that clarifying the scope of our CNN-based analyses will address the concerns about the model voiced by R2 and R3. However, we also realized that the CNN analyses perhaps were too prominent and distracted from some of the interesting neural findings, particularly when it comes to the spectral analysis. We thus removed the methods, results and discussions of the CNN model analysis for the spectral responses from the main manuscript. We believe that this has helped to streamline the manuscript and better focus on the most essential findings.

RESPONSE TO REVIEWERS

Please find the detailed point-by-point response below. Reviewer comments are highlighted in grey, our responses are shown in blue, and changes made to the manuscript are indicated in **bold blue** text. All corresponding revisions have also been incorporated into the manuscript itself, where changes appear in red.

REVIEWS:

Reviewer #1 (Ke Jia): This study by Xie and colleagues combined a backward masking paradigm with fMRI and EEG to investigate the mechanisms of recurrence on visual representations in the human brain. By comparing the early and late masking conditions, they found the spatiotemporal dynamics of changes in representation during recurrence. They further used a deep neural network and revealed four distinct spectro-temporal neural components in EVC and LOC. Overall, the research question is interesting and the authors used a lot of sophisticated analyses. However, there are several confounding factors that have not been controlled very well and I have some major concerns that need to be addressed.

We thank the reviewer for the critical and constructive comments.

Major concerns:

1. I agree with the authors that backward masking can potentially affect the recurrent or feedback processing. But the difference between early and late masking conditions might also be

confounded by other factors. For example, the overall signal to noise ratio may be lower for the early than the late masking conditions, especially between ~100 ms (i.e., the first peak) to ~600 ms (i.e., the onset of the masking in the late condition), as the authors only added noise to the visual systems for the early masking condition during this period. The reduced SNR could also explain other findings presented in the manuscript: a) within vs across conditions decoding using EEG data; b) differences in temporal generalization; c) results of the fMRI data. These confounding factors make the conclusion less convincing.

This is an important point - we appreciate the concern that differences in SNR could contribute to the observed representational differences between early and late mask conditions. However, our supplementary analyses suggest that SNR alone cannot account for our findings.

In these analyses (see Rebuttal Fig. 1 reproducing Supplementary Fig. 1D, Supplementary Fig. 4B, Supplementary Fig. 3D-F), we compared within-early-mask-condition decoding (i.e., training and testing on early mask trials) to across-condition decoding (training on early mask trials and testing on late mask trials). If reduced SNR in the early mask condition were the primary driver of the observed effects, we would expect within-early-mask-condition decoding accuracy to be noticeably worse, especially in the time window from ~100ms to ~600ms, when the early mask could be reducing SNR (Supplementary Fig. 1D or Rebuttal Fig. 1A). Contrary to that expectation, within-condition decoding in the early mask condition was actually higher than across-condition decoding (i.e., the green curve has higher decoding accuracy than the black curve). This pattern implies that, despite any potential SNR reduction from the early mask, the neural representations within this condition remain robust enough for training and testing on those same trials. In contrast, when classifiers are trained on early-mask data and tested on late-mask data (or vice versa), decoding accuracy drops heavily, indicating a fundamental difference in the underlying representations between these conditions. Similarly, we observed higher decoding accuracies for within-condition decoding in the early mask condition than across-condition decoding in both fMRI data (Supplementary Fig. 4B, or Rebuttal Fig. 1B) and the time generalization analysis (Supplementary Fig. 3D-F, or Rebuttal Fig. 1C).

We thus interpret the difference between early and late mask decoding as reflecting the difference between the presence (late mask) or disruption (early mask) of recurrent/feedback processes rather than SNR differences. In other words, while the early mask may indeed introduce some reduction in signal strength, it is the altered representational geometry, likely due to less recurrence, that best explains why cross-condition decoding deteriorates and why within-condition decoding remains relatively strong.

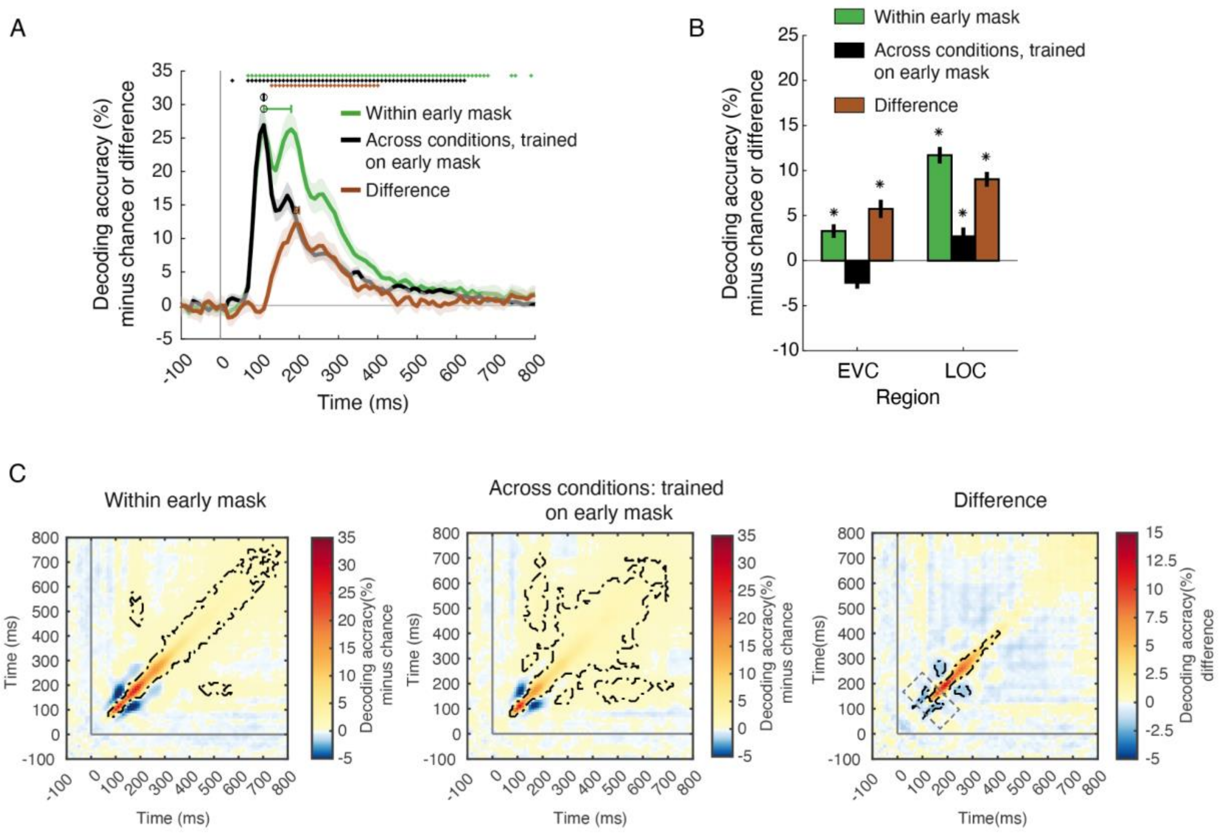

Rebuttal Figure 1 Pairwise object identity decoding results within-early-mask condition and across masking conditions, along with their differences*. The results are presented separately for the temporally resolved decoding analysis for EEG data (A), spatially resolved decoding analysis for fMRI data (B), and time generalization analysis (C).*

We add the following text in the Discussion to clarify why differences in SNR do not fully account for our main findings:

P. 12-13, ll. 387-394 (Discussion):

> **"An alternative explanation is that the early mask condition yields a lower signal-to-noise ratio (SNR) than the late mask condition. However, the supplementary within-condition versus across-condition decoding analyses show that within-condition decoding in the early mask condition remains robust, whereas across-condition decoding between early and late masks relatively deteriorates (Supplementary Fig. 1D, Supplementary Fig. 4B, and Supplementary Fig. 3D-F). This pattern suggests that the SNR in the early mask condition allows for robust decoding, so that the observed effects are plausibly attributable to altered (i.e., disrupted) recurrent processing rather than SNR differences."**

2. I can not find the display information for the EEG and fMRI experiments. This is important because certain displays may show residual images following the offset of the stimuli. This

represents another confounding factor that could explain the difference between early vs. late masking conditions, within vs across conditions decoding analysis and the results of the fMRI data. The authors need to provide more details or conduct control analyses to exclude this possibility.

Thanks for pointing this out. We provide details about the presentation equipment.

P. 16-17, ll. 552-554 (Materials & Methods):

> **"In the EEG experiment, stimuli were presented on a Samsung 2233RZ monitor with a refresh rate of 60 Hz. In the fMRI experiment, stimuli were projected using a Canon SX60 multimedia projector, also at 60 Hz."**

We have used these display settings in other timing-critical experiments and are confident that there are no visible residual images. We do appreciate the concern that relatively prolonged residual images in the late mask condition (which are extinguished by the mask in the early mask condition) may have caused differences between conditions. However, previous results suggest that stimulus duration does not have a huge effect on decoding performance [1,2], even when stimulus duration is manipulated with a fully visible high-contrast stimulus. Any effect of a lingering residual image in the late mask condition is thus bound to be extremely weak.

In addition, I have some minor concerns:

1 In line 76-77, the authors state that 'Because target images and masks were statistically independent by design across trials, classifying objects isolated the neural activity that related to the target from neural activity related to the mask'. Please explain the logic more clearly (i.e., why the authors can 'isolated the neural activity that related to the target')

We rephrased the sentence as follows:

P. 3, ll. 80-85 (Results):

> **"Across trials, the masks were randomly paired with the stimuli. Thus, the successful classification of target images is based on the neural activity elicited by the target image only, even if it overlaps with neural activity elicited by the mask, as the mask content was independent (and thus not predictive) of the target image."**

2 In line 110-116, the authors found a double-peak pattern in the EEG decoding analysis. Is this result in line with previous research? What is the functional role of the double peak?

The double peak pattern is an interesting phenomenon. It is in line with previous research, where a double peak pattern is often seen in M/EEG decoding studies (see examples in Rebuttal Fig. 4).

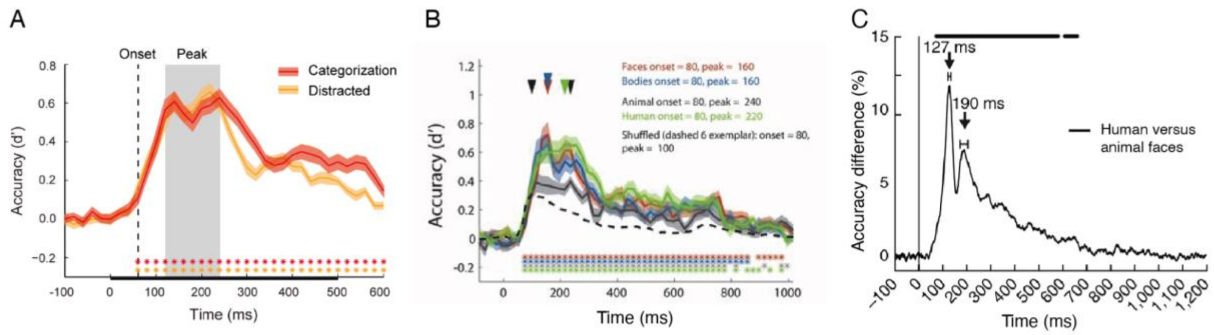

Rebuttal Figure 4 Double peaks pattern in decoding time courses in previous studies. *The double peak pattern is observed e.g. in (A) Ritchie et al. (2015)* [3]*, (B) Carlson et al. (2011)* [4]*, (C) Cichy et al. (2014)* [5]*.*

However, the functional role of these two peaks is unclear, and at this point we can only share speculations. One idea is that the dip between the peaks might also indicate an underlying oscillatory cycle at ca. 10 Hz, leading to "valley" in the decoding signal. Another idea is that the first peak represents predominantly the feedforward sweep, and the second peak represents recurrent activity. This is supported by previous invasive work [6,7], and also by our analyses. For example, our results indicate a change of visual features representations between the two peaks (Fig. 3D, E). At ~100ms, i.e., the first peak, in both the early and late mask conditions, the EEG signals mainly relate to the early and middle layers of the DNN, indicating representations of low complexity; while at ~180ms, i.e., the second peak, they relate less to early and middle, and more to later layers, indicating representations of higher complexity. Thus, the two peaks might indicate two processing stages.

Given that the thoughts above are speculations and neither supported nor rejected by the reported data, we decided to not discuss them in the manuscript.

3 In line 256-265, the authors used the 'shift in the peak correspondence' to investigate the representation format in the fMRI data. In figure 3C, layer 4, layer 5 and layer 8 exhibit high spearman correlation between fMRI RDM and DNN RDM both for the early and the late masking conditions. Is the shift consistent across participants? Please use some statistical methods to support their conclusion.

We appreciate the opportunity to clarify the consistency of the difference (shift) in peak layers across participants (see Rebuttal Fig. 5A). To assess whether this difference was statistically reliable, we performed a permutation test on the participant-level differences in peak DNN layer between the early and late mask conditions. The test empirically builds a null distribution by randomly re-assigning condition labels within each participant and recalculating the mean difference across participants for each permutation (see Rebuttal Fig. 5B). We report the two-tailed p-value and the effect size, calculated using Hedges' g, of the observed difference. This result confirms a systematic difference in peak layers across participants, supporting our conclusion.

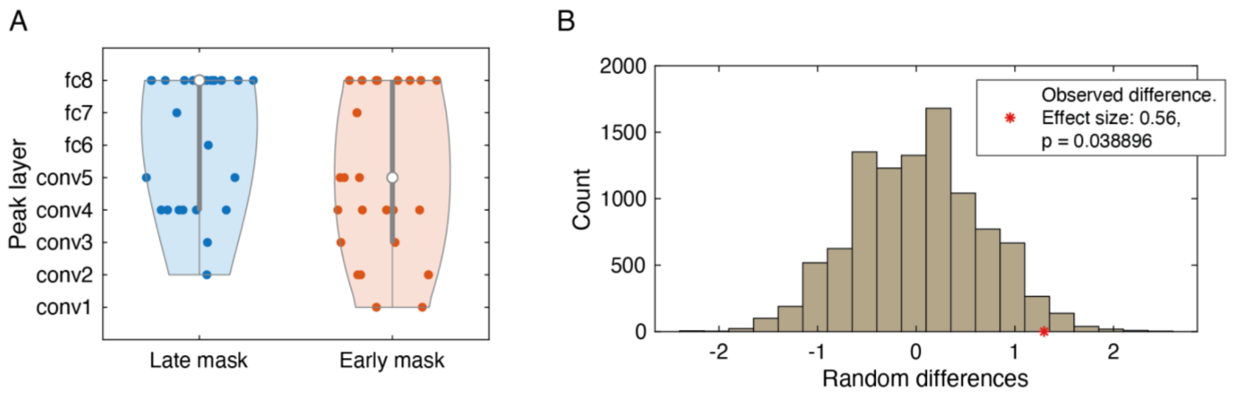

Rebuttal Figure 5 Peak layer differences between early and late mask conditions in the RSA result linking LOC to DNN layers. *(A) Violin plot showing each participant's peak layer in the late mask and early mask conditions (N = 27). The white dot marks the median, and the vertical thick grey bar indicates the interquartile range. (B) Permutation test results showing the observed mean difference relative to the null distribution. The red asterisk marks the observed difference, with an effect size of 0.56 (medium effect) and a two-tailed significance level of p = 0.039.*

This information has been added to the text.

P. 9, ll. 269-274 (Results):

> **"First, although both masking conditions showed correspondence with middle to deep DNN layers (layers 3–8; Fig. 3C), the peak correspondence differed: in the early mask condition, it occurred in a middle layer (conv4; [conv4, fc8]), whereas in the late mask condition, it shifted to the deepest layer (fc8; [fc8, fc8]). This shift had an effect size of 0.56 (Hedges' g) and was statistically significant (p = 0.039, two-tailed permutation test)."**

P. 24, ll. 849-853 (Materials & Methods):

> **"To assess whether the difference in peak layers between masking conditions was statistically reliable, we followed the sign permutation procedure, generating a null distribution by randomly reassigning condition labels within each participant and recalculating the mean difference across 10,000 permutations. We report the two-tailed p-value and the effect size of the observed difference, calculated using Hedges' g."**

Reviewer #2: The study by Xie et al. has investigated recurrent processes in the visual system. The authors have been using a simple but informative paradigm where the presentation of visual stimuli is masked 17 ms after the display while brain activity was recorded using MEG and fMRI. The assumption is that the mask abolishes feedback or recurrent processing. By comparing masked vs unmasked presentations, it is possible to uncover when and where the recurrent processing occurs. Furthermore, the findings are linked to CNNs in order to substantiate the argument on processing

in early versus late regions. The core finding is that recurrent processing indeed is present at ~100 ms in early visual cortex and then emerges at ~175 and ~300 ms in lateral occipital cortex. This demonstrates that the recurrent activity is persistent rather than transient in lateral occipital cortex. A similar analysis is conducted in the spectral domain. This reveals an early effect peaking at 160 ms in the theta-alpha range and an alpha-beta effect peaking at 540 ms. The linking between brain data and CNNs is based on a representational similarity analysis correlation the respective RDMs. This analysis revealed that recurrence effects were associated with the high-level feature complexity of representations in lateral occipital cortex rather than the early visual cortex.

These are informative findings as they speak to visual processing going beyond the first feedforward visual sweep. It is somewhat a surprise that the recurrent processing emerges already at 100 ms in early visual cortex and then appear in lateral occipital cortex at 175 ms. To me this order suggests that the recurrent processing is not due to a feedback sweep (but the authors might want to speculate on this).

We thank the reviewer for their clear summary and the constructive feedback. We respond to each specific comment below.

Specific comments

The write-up is somewhat unspecific on recurrent versus feedback processing. I think it would find it helpful to define recurrent processing as processing within one layer or region explained by lateral connectivity. This is different from feedback processing reflected by the flow from higher to lower-level regions. Clarifying these issues would be useful when interpreting the findings of the paper.

We thank the reviewer for the suggestion to clarify our use of the terms "recurrent" and "feedback" processing. We indeed do not differentiate between (local) recurrent and (longer-range) feedback processing in our paper. This is partly because the field has no unified way to group and distinguish terms such as recurrent, local- and long-range feedback, re-entrant, etc. Here we thus conceptualize recurrence widely as a superset that encompasses local recurrence (e.g. lateral connectivity), as well as short- and long-range feedback. This use echoes that of, for example, Kar et al. (2019) [7], who included within-region lateral as well as between-region cortico-cortical, subcortical, and medium- to long-range intra-areal recurrent circuits in the term recurrence. Further, our study does not provide evidence at the neural circuits level that would allow us to distinguish between the various kinds of recurrent activity, necessitating a broader definition of recurrence for our purpose.

We clarify our terminology in this regard at the beginning of the introduction:

P. 2, ll. 36-38 (Introduction):

> **"Anatomically, this is mediated by feedforward as well as recurrent connections. Here, we define recurrence broadly, including lateral connections within a cortical**

region, as well as short- and long-range, cortico-cortical and subcortical-cortical feedback (1–3).”

I find it surprising that the recurrent processing is present already after 100 ms in early visual regions - and then later in the lateral occipital cortex. This suggests that recurrent activity is not explained by a feedback signal propagating backwards in the ventral stream. Rather the results are in line with local recurrent processing - or the fact that the visual display is changed after 17 ms in the masked condition. The latter concerns could be made explicit in the Discussion when discussing limitations. Also, would an off-response after 17 ms not result in a representational specific activation?

We thank the reviewer for the opportunity to clarify. We structure our response into two parts.

(1) Timing of feedback effects

We agree that one possibility is that the early masking effect indicates recurrent activity through local lateral connections (such as connections within V1). However, another possibility is that it indicates feedback activity within the early-to-mid-level regions of the ventral visual stream. Assuming an inter-regional transmission delay of ~15ms between successive stages, and first feedforward signals reaching V1 at 50ms, they could propagate to V2 at 65ms and return to V1 via feedback at 80ms; reach V3 from V2 around 80ms, and return back to V2 at 95ms, and so on so forth. Though perhaps less likely, even response latencies in frontal regions such as FEF are very short (sometimes even shorter than in V2 [8]). Following this logic, recurrent activity from higher-level regions (both within the ventral visual stream and beyond) can in principle reach early visual areas very rapidly.

Thus, the temporal order of masking effects (first in EVC, then in LOC) does not rule out that the early effect is due to local or long-range feedback from a distant area.

P. 13, ll. 402-407 (Discussion):

> **“In EVC, recurrence affected visual representations rapidly, with a peak effect at 100ms. This is in the range of previously observed early effects of recurrence in non-human primate EVC (7), associated with contextual modulation and figure-ground segregation (92–94), where feedback signals originate from within the ventral visual stream. Our study does not allow for dissecting different types of recurrence, and the observed effects could stem from recurrent activity within (early) visual cortex but also from rapid long-range feedback from beyond visual cortex (33,95)”**

(2) The role of the offset response

We do not believe that the offset response has a strong influence on the results here. This is because visual offset responses gradually increase with the duration of image presentation. As a direct anchor point, at a stimulus duration of 200ms and 500ms, decoding from offset responses is very

weak compared to decoding from the onset response [4,5] (reproduced below, Rebuttal Fig. 6A, B, where the red circles indicate offset response). Extrapolating this effect to the very short image duration here (17ms) suggests that the contribution of the offset response to the signal is negligible, especially compared to the effect sizes for the late versus early mask contrast.

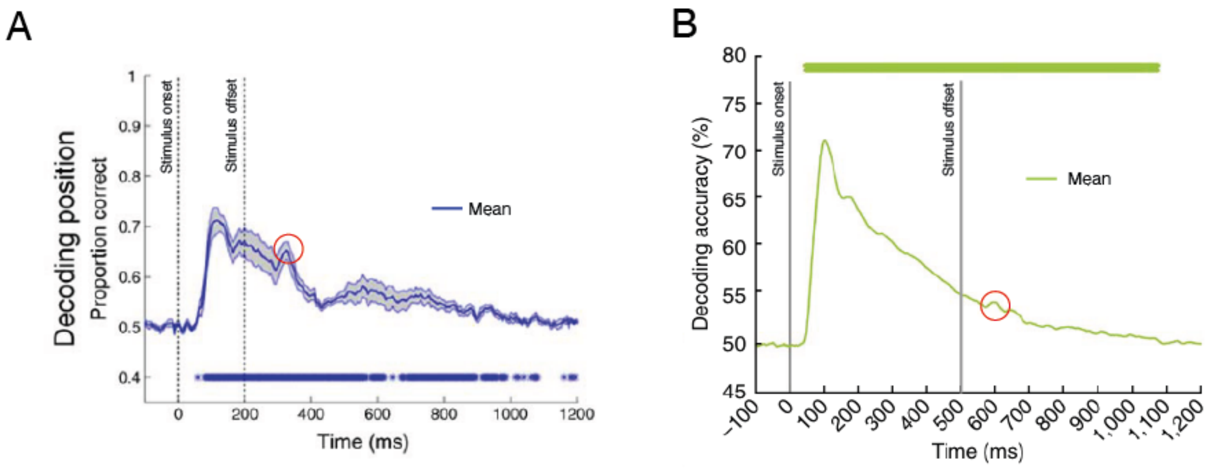

Rebuttal Figure 6 Decoding from image offset responses in previous studies. *The decoding accuracies highlighted with red circles correspond decoding peaks elicited by the offset after 200ms in (A) Carlson et al. (2011)* [4] *and after 500ms in (B) Cichy et al. (2014)* [5].

The AlexNet used in the manuscript is purely feedforward. The authors do acknowledge that the aim is not to model brain activity per se, but then it is not so clear to me what we actually learn from the linking to CNNs. CNNs with recurrent properties (e.g CorNet-S) have been developed and might have been more appropriate? This is briefly mentioned and could be more thoroughly elaborated upon (see e.g., Kubilius, J., Schrimpf, M., Nayebi, A., Bear, D., Yamins, D. L., & DiCarlo, J. J. (2018). Cornet: Modeling the neural mechanisms of core object recognition. BioRxiv, 408385.)

We are happy to clarify this. We indeed did not use CNNs to model brain activity per se, but as a tool to assess the complexity of the feature format of neural representations, leveraging the increase in feature complexity across CNN hierarchies. We agree with the reviewer that recurrent CNNs (like CorNet and successors) are well suited for modelling recurrent dynamics in cortex. However, for our humbler goal of quantifying feature complexity, a feedforward CNN is well suited, as previous work provides us with precise information about how feature complexity evolves across feedforward networks like AlexNet. Recurrent CNNs are comparably less suitable to quantify feature complexity in a straightforward way, given the additional temporal dimension and relatively less work on feature evolution across the networks.

Finally, we agree that explicitly modelling recurrent neural computations is an exciting future topic, and we thus expanded our discussion on this.

P. 14, ll. 456-461 (Discussion):

> **“Please note that here we used feedforward DNNs as an established tool to quantify visual feature complexity (68–70), rather than as a model of recurrent human visual processing. For modelling recurrent visual processing, recurrent DNNs (138–141) are needed. This is a burgeoning research field, delineating how feedforward and recurrent activity (21,141,142) respectively account for core object recognition (16), as well as visual behavior (140). We believe our empirical data could serve as an interesting benchmark for model fitting.”**

The spectral analysis is written as if the effects reflect modulation in oscillatory activity. How do we know that the reported results are not due to evoked activity - which evidently will be reflected in the spectral domain? I would suggest that the authors do not equate spectral findings with oscillations when introducing and describing the results, but bring up the different interpretations (oscillatory versus evoked activity) in the Discussion section.

We thank the reviewer for the cautionary note that helped us to sharpen our claims. We fully agree that we should not equate the results of the spectral analysis with effects on oscillatory activity.

We have thus revised the text such that all results and conclusions related to the time-frequency resolved analyses do not mention oscillations, but refer to spectral components, power and phase changes, etc.

P. 11, ll. 341-342 (Results):

> **“The transmission of visual information in feedforward and recurrent fashion is fundamentally indexed by neural activity in distinct frequency bands (78,79).”**

P. 14, ll. 463-464 (Discussion):

> **“Our results extend prior work by identifying four distinct spectro-temporal components (theta to beta range) with specific temporal profiles that subserve recurrent processing (45,143–145).”**

We also expanded the Discussion to explicitly consider alternative explanations, that is, how phase-locked evoked responses might contribute to the observed spectral signatures versus non-phase-locked (“induced”) oscillations.

P. 15, ll. 473-477 (Discussion):

> **“While our spectral analysis reveals robust stimulus-related changes in power and phase, it is important to note that these spectral signatures may reflect phase-locked evoked transients rather than intrinsic, non-phase-locked oscillatory dynamics. Thus, although we observe spectral modulations, future work is needed to disentangle oscillatory from non-oscillatory components (57,159,160) underlying these effects.”**

The recurrent activities are likely to be highly non-linear. The signals are not simply the sum of feedforward and recurrent activity but rather a complex interaction between both. Please consider this when subtracting the masked versus unmasked conditions and interpreting the results (e.g. Figure 6G (line 424 - 426), 'the spectral basis of recurrent activity in LOC is in power only '; 'recurrent activity is less phase-locked'. Is it for instance possible that the recurrent activity is phase-specific, but it does not affect the feedforward processing in a phase-specific way?

We thank the reviewer for the cautionary note. We see that our interpretation was too strong, and as our main claims do not depend on the problematic statements, we removed them from the manuscript. Also, in response to reviewer 3, we moved Figure 6 to the supplementary material.

We have concerns on the oscillatory component explanation of the off-diagonal negative accuracy. There is no good reason for the representation of a certain stimulus being time-locked with a half-cycle phase shift. Moreover, there are lots of object pairs, so the phase shifts (if they exist) should cover a wide distribution. They cannot always be in anti-phase with each other (if A is anti-phased to B and B is anti-phased to C, then A should be in-phase with C). As such this could be more carefully phrased.

We thank the reviewer for raising this point. We see that our point was not explained well. We have carefully rephrased our description to make clear that the half-cycle shift was used as a simplified example of systematic, stimulus-locked changes in oscillatory phase patterns. We have revised the text to clarify our reasoning.

P. 5, ll. 172-176 (Results):

> **"How are the negative decoding accuracies in off-diagonal regions of the TGA to be explained? While commonly observed (51,55), the underlying neural dynamics are unknown. One tentative idea is that they might reflect systematic, stimulus-locked shifts in oscillatory phase patterns between the two time points at which the negative decoding occurs (56,57) (for graphical illustration, see Supplementary Fig. 2)."**

Supplementary Fig. 2:

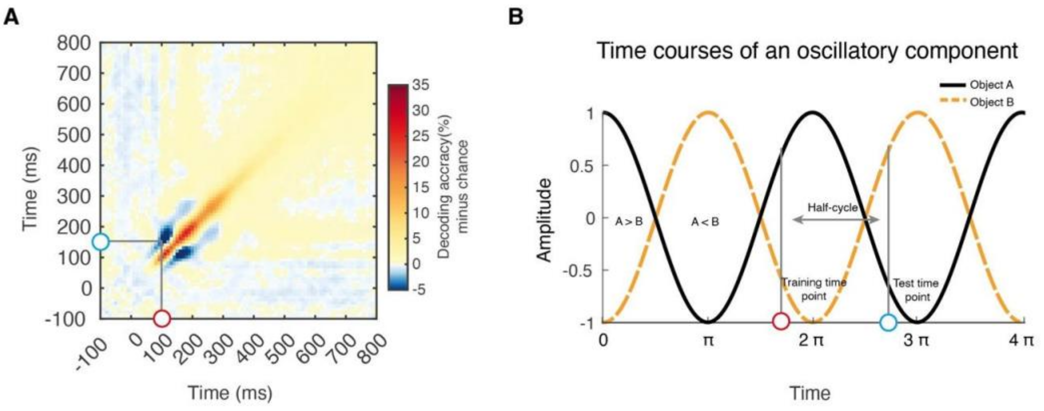

"Supplementary Figure 2 Potential neural source of negative off-diagonal decoding accuracies in temporal generalization analysis. (A) Results of time-generalized object identity decoding in the early mask condition, as shown in Fig. 1E. Blue and red dots indicate an example pair of corresponding time points for negative off-diagonal decoding accuracies. ***(B) Hypothetical oscillatory mechanism underlying negative off-diagonal decoding. Two identical, time-locked responses for object A and B differ in phase (black solid curve and orange dashed curves). For illustration, the phase shift is set to half a cycle (π), corresponding to the time difference between two off-diagonal time points identified in (A) (blue and red circles). At time point 0π, signal A > signal B, whereas at π, signal B > signal A, illustrating a reversal in relative amplitude. A classifier trained at 0π may thus predict object identity at π with opposite labeling, resulting in below-chance classification accuracy."***

One line 426-428 the authors write: 'This suggests an increase of variability in phase over the course of processing, possibly due to accruing variability as information propagates increasingly back and forth along the visual processing hierarchy.' We are not sure if this conclusion appropriate. The difference on the LOC cannot represent the full recurrent processing. In contrast, the EVC difference shows an increased coefficient of α/β phase component, which could then be interpreted as an increase of phase specificity after the information comes back to EVC.

We thank the reviewer for pointing this out. This point echoes the previous point, and since this was only a tentative interpretation rather than a solid conclusion, we have removed the claim from the manuscript.

Line 207 - 209 seems to be a wrong description of the Figure2C? (in regard to bar colors for different conditions)

Thanks! We corrected the error.

Line 430: 'Using deep neural networks to model the representations from EEG and fMRI data (45-47), we found that recurrence changes the feature complexity of representations in LOC, but not in EVC.' This seems too bold of a claim as the DNN is a simple model not including recurrents.

We have revised the text to clarify that the feedforward DNN serves as a tool to quantify representational feature complexity rather than a direct model of recurrence.

P. 14, ll. 431-432 (Discussion):

> **"Using a feedforward DNN to assess feature complexity, we found that recurrence modulates representations in the lateral occipital cortex (LOC) but not in early visual cortex (EVC)."**

Line 527: 'We created a set of image textures to be used for dynamic backward masks. For this, we chose a different subset of 24 object images randomly from the 118-image set and converted the images to textures that conserved the low- and mid-level image statistics of the images without portraying identifiable objects (153).' This step requires more explanation.

Our goal was to create textures that preserve the general low- to mid-level image statistics such as spatial frequency and orientation content, but at the same time remove high-level features that might render the original objects recognizable. To achieve this, we used the parametric texture synthesis algorithm, provided by [9] to iteratively re-synthesize each of the 24 chosen object images into a texture. This algorithm analyzes and matches various spatial statistics (e.g., orientation, scale, phase correlations) in overlapping patches of the source image, ensuring that the resulting texture retains similar color, luminance, and local orientation distributions but discards coherent shape cues. We then verified visually that none of the resulting textures contained identifiable objects.

We have added a detailed description of these steps to the Methods section.

P. 16, ll. 526-534 (Materials and Methods):

> **"Each selected image was converted into a texture using the parametric synthesis method described in (163). This algorithm analyzes and matches various spatial statistics (e.g., orientation, scale, phase correlations) in overlapping patches of the original source images, ensuring that the texture of the resulting image preserves similar color, luminance, and local orientation distributions as the source while discarding coherent shape cues. We then visually confirmed that none of the resulting textures contained identifiable objects. Finally, to create each dynamic mask, we randomly selected 12 textures from our pool of 24 and arranged them in a random sequence, yielding 24 distinct 12-frame masks."**

The use of the HRF is confusing. At some point a voxel specific is derived from the data and later a canonical HRF is used. Please clarify.

Thank you for raising this point. Our workflow involves two separate HRF modelling procedures, each serving a different goal:

(1) We use voxel-specific HRFs for the main experiment runs that have an event-related design. To capture regional and individual variability in BOLD timing, we estimated an empirical hemodynamic response function for each voxel. This data-driven HRF was then used to model voxel responses in the main runs, improving the precision and sensitivity of any analyses that depend on accurate HRF shape (see [10]), using methods established for event-related designs.

(2) We used a canonical HRF for the functional localizer run that had a block design. For defining each participant's early visual cortex (EVC) and object-selective lateral occipital complex (LOC), we fitted a standard (canonical) HRF, suitable for the block design. This is the standard approach in localizer analysis.

In the manuscript we clarify which analyses relied on the voxel-specific HRFs and which used the canonical HRF.

P. 19, ll. 653-672 (Materials and Methods):

> **"We used general linear models (GLMs) to estimate responses for the 48 experimental conditions (i.e., the 24 object images presented in either the early mask condition or the late mask condition) in the main experimental runs and responses for 3 experimental conditions blocks (i.e., objects, scrambled objects, and baseline) in the localizer runs.**
>
> **The analysis was conducted in a participant-specific fashion. In all cases, we applied the GLM estimation to the preprocessed fMRI data for each run. We entered experimental condition onsets and durations as regressors into the GLM. Nuisance regressors comprised noise components and movement parameters. Our workflow involves two separate HRF modelling procedures using GLMs.**
>
> **First, for the experimental runs with an event-related design, we used voxel-specific HRF modelling. To capture regional and individual variability in BOLD timing, we evaluated 20 different GLMs by convolving regressors with 20 distinct hemodynamics response functions (HRFs) as derived from a large fMRI dataset (170) for each voxel. We then identified the HRF that resulted in the lowest average residual (171) per voxel and chose the corresponding estimates for further analysis.**
>
> **Second, for the localizer run with a block design, we used the canonical HRF. We computed two contrasts from the resulting GLM parameter estimates that were used at a later step for voxel selection in the ROI analysis. The first contrast was defined as object + scrambled objects > baseline to define EVC. The second contrast was defined as objects > scrambled objects to define LOC. This yielded two t-value maps for the localizer run per participant."**

Line 842: ' …. clusters based on temporal or spatial contiguity with a p < 0.005 (i.e., cluster-definition threshold) for most analyses, except for time-frequency decoding matrices, which used a threshold of p < 0.05'. Why were different thresholds used?

We realized that using different significance thresholds does not make much sense here. We thus re-ran all cluster-based tests using the stricter **p < 0.005** threshold.

The overall results pattern remains unchanged, although unsurprisingly with the higher threshold, the significant clusters are somewhat smaller. We have updated the manuscript accordingly. For direct comparison, below we reproduce the previous version (p<0.05) and the current version (p<0.005) side by side in Rebuttal Fig. 7 and Rebuttal Fig. 8.

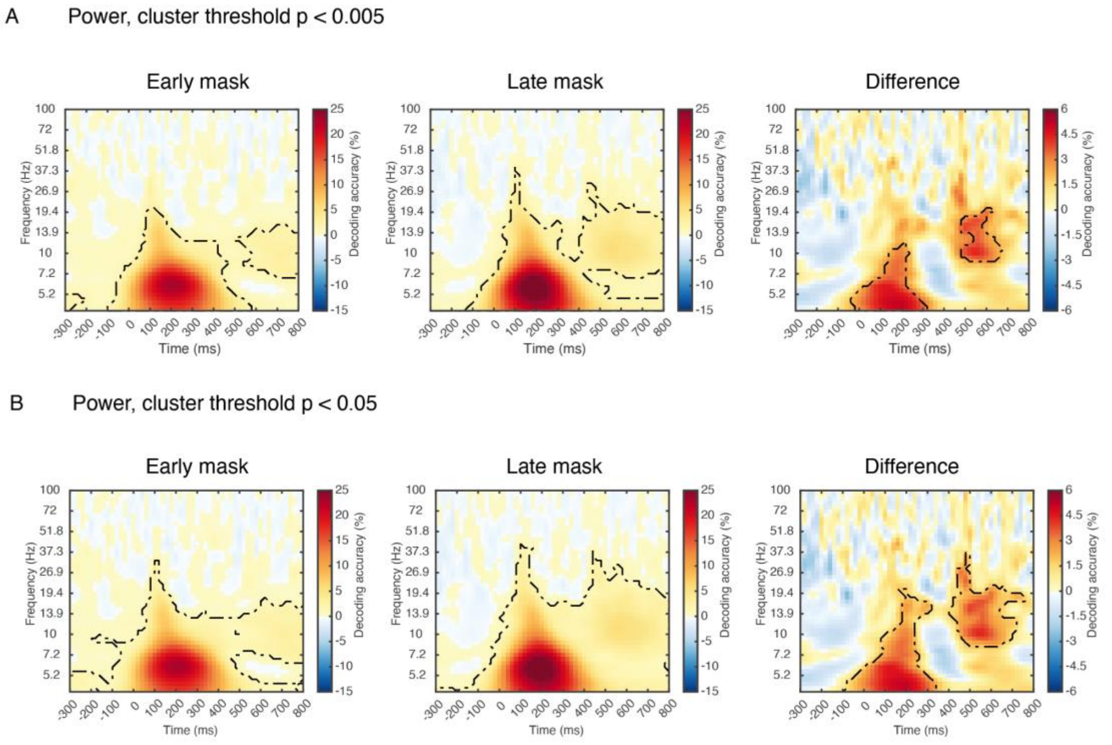

Rebuttal Figure 7. Power decoding. *(A) Permutation test results using a more stringent cluster threshold (p < 0.005) as applied in the current version of the manuscript. (B) Results using the original cluster threshold (p < 0.05) from the previous version of the manuscript.*

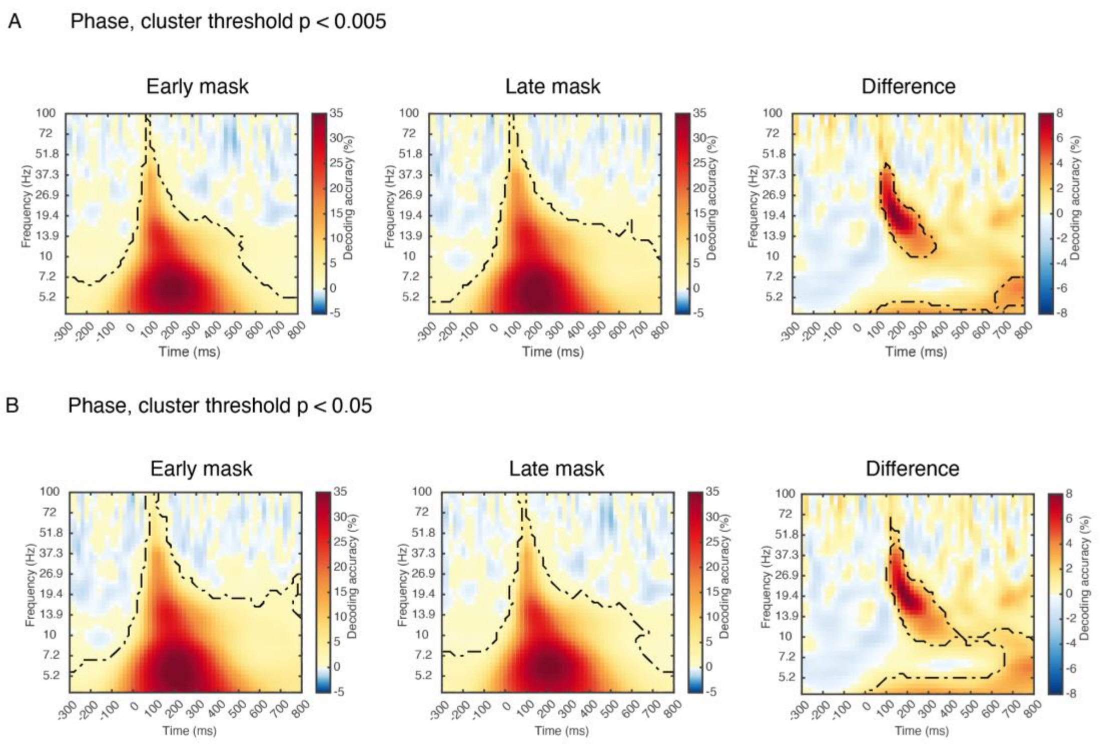

Rebuttal Figure 8. *(A) Permutation test results using a more stringent cluster threshold ($p < 0.005$) as applied in the current version of the manuscript. (B) Results using the original cluster threshold ($p < 0.05$) from the previous version of the manuscript.*

In the same spirit, we also updated Figure 5, using a cluster threshold of **$p < 0.005$** to ensure consistency across the manuscript. The pattern of results remained the same.

Finally, since the extension of the components slightly changed when using the cluster threshold of **$p < 0.005$**, we also re-ran the analysis for Figure 6 (now Supplementary Fig. 8). Also, here the pattern of results remained unchanged.

The English could be improved.

Somewhat unusual formulations:

'…is incompletely understood due…'

'We observed a qualitatively similar and typical results pattern (48,49) in both masking conditions.' Not so clear that typical refer to.

'…conducted in a participant-specific fashion…'

At places many adjacent sentences start with We: ('We …. We…')

We thank the reviewer for this advice. We let a native English speaker revise the manuscript.

Reviewer #3: The interplay between feedforward and recurrent processing is one of the major questions in neuroscience. In the current study, the authors address this question by a combined EEG, fMRI, and CNN study, finding for example that recurrent processing affects feedforward processing already at 100ms. The experiments are carried out in an expert fashion with carefully chosen stimuli. The analysis methods are state of the art and the combination of the various analyses makes this paper an important contribution. The ms is extremely well written and even though the figures are packed they are very clear.

We thank the reviewer for the positive evaluation.

Majors

I do not see what the CNN analysis can tell. Alexnet, nor any other CNN even if recurrent, is a validated model of the human visual system (what human area would correspond to layer 8 in the net?). The authors could as well have used a model for weather forecasting. Btw isn't Alexnet a feedforward net? In any case, I recommend dropping this part entirely.

We see that we need to clarify the CNN analysis better. We fully agree with the reviewer that no existing CNN (whether recurrent or not) is a one-to-one, perfect model of the visual system. Yet, CNNs do reasonably well in some regards, including the prediction of large-scale representational geometry in visual cortex and the approximation of visual feature hierarchies along the ventral stream. The latter point is what we capitalize on here, as we point out in the following.

To be clear, accurately modelling the hierarchical processing stages of the visual system was not the goal here. We rather used the CNN as a tool for assessing the feature complexity in neural representations. Layers in AlexNet architecture increase in feature complexity along its hierarchy [11]. We use this property of AlexNet (and other CNNs) to assess the feature complexity of visual representation in the brain. The CNN analysis thus informs us about the neural feature format on a scale from simple to complex. As CNNs have been shown to mimic the progression from simple to complex features that happens in the visual system, relative feature complexity in the DNN is informative for assessing relative feature complexity in the biological visual system. This argument holds in the absence of a direct mapping between CNN layers and brain regions. For our goal of quantifying feature complexity, a feedforward CNN like AlexNet is well suited, as previous work provides us with precise information about how feature complexity evolves across feedforward networks like AlexNet. Recurrent CNNs are comparably less suitable to quantify feature complexity in a straightforward way, given the additional temporal dimension and relatively less work on feature evolution across the networks.

We believe this approach has merit and adds interpretable information about representational formats. We also realize that our previous manuscript was perhaps too reliant on the CNN findings, which overshadowed some of the interesting neural findings, particularly in the spectral domain. We thus decided to remove the CNN analyses for the spectral results from the manuscript.

We modified the manuscript to clarify our use of the CNN here better. In particular, we changed all phrases that were vague or were misleading, for example, exchanging the phrase "computational modelling" to "computational model comparison as a tool."

P. 12, ll. 366-368 (Discussion):

> **"We combined a backward masking paradigm with multivariate analysis on EEG and fMRI data, along with computational model comparison as a tool, to characterize when, where and how recurrent processing affects object representations."**

Further example changes include:

P. 8, ll. 250-252 (Results):

> **"We used a feedforward DNN as a tool to examine how feature representations of different complexity emerge across space and time when recurrence is intact or disrupted."**

P. 14, ll. 431-432 (Discussion):

> **"Using a feedforward DNN to assess feature complexity, we found that recurrence modulates representations in the lateral occipital cortex (LOC) but not in early visual cortex (EVC)."**

We also explicitly clarified why we did not use the recurrent DNN.

P. 14, ll. 456-461 (Discussion):

> **"Please note that here we used feedforward DNNs as a helpful and established tool to quantify visual feature complexity (65–67), rather than as a model of recurrent human visual processing. For modelling recurrent visual processing, recurrent DNNs (133–136) are needed. This is a burgeoning research field, delineating how feedforward and recurrent activity (18,136,137) respectively account for core object recognition (13), as well as visual behavior (135). We believe our empirical data that we provide could be an interesting benchmark for model fitting."**

Why did the authors use an ISI of 600ms for the late mask? I think this is equivalent to a no mask condition, which is missing.

Thanks for pointing this out. We agree with the reviewer that this is equivalent to a no mask condition in that all effects we observe and interpret are before 600ms.

We opted to present a mask rather than no mask so that both conditions (early vs. late) have the same basic visual elements, varying only in timing. Had we omitted the mask entirely in one condition, then the total visual input across conditions would have differed substantially (i.e., early

mask vs. no mask at all). This would have led to less controlled comparisons, in particular for the fMRI analysis for which the BOLD response to the target and the mask overlaps strongly. Further, adding a third condition (no mask at all) would have lengthened the experiment by 50%. Thus, the compromise was to present a mask as early after the image as possible, but late enough so that it does not affect the recurrent processes we are interested in investigating - resulting in 600ms.

We further clarify the choice of 600ms in the manuscript.

P. 16, ll. 545-546 (Materials and Methods):

> **"Note that the late mask condition, until 600ms, corresponds to a condition without a mask."**

The task does not reflect object recognition but the entire discussion is about it.

We agree that the task does not reflect object recognition. Although the term is commonly used when neural computations in the ventral stream are assessed, we realize that in the context of our manuscript, this might cause confusion as there was no behavioral recognition task.

In the current version of the manuscript, we abstain from mentioning object recognition at all in the discussion section, and we added an explicit statement that our findings concern visual representations, not object recognition.

P. 13-14, ll. 426-429 (Discussion):

> **"A limitation of our work is that it characterizes visual representations without directly linking them to object recognition. Further work should relate the observed effects to behavior, for example by contrasting correct and error trials (118,119) or linking neural activity to behavior on a trial-by-trial basis (120,121)."**

I did not get what the analysis about power and phase is about.

Thanks for the opportunity to clarify. The results pattern we observe is complex, and we see that our presentation of the analysis and its results were not clear enough. We have thus restructured and shortened this part of the manuscript.

In short, based on previous literature, we expected the spectral basis of recurrent activity to be in a low frequency range. We thus analyzed the EEG data in time-frequency resolved fashion, distinguishing power and phase. Our major contribution was the novel identification of four components (distinct in timing and frequency) that differ for the early versus late mask condition, and we thus interpret as relating to recurrent activity (Fig. 5). We kept this section and rewrote the text, streamlining the argumentation.

P. 11, ll. 341-347 (Results):

> **“The transmission of visual information in feedforward and recurrent fashion is fundamentally indexed by neural activity in distinct frequency bands (78,79). Based on previous work in human and non-human primates, we hypothesized that recurrent processing should be evident in the low-frequency range between the theta- and the beta-range (80–83). Thus, in the next step, we investigated the spectral characteristics of visual processing in the early mask condition and the late mask condition. For this, we decoded object identity from EEG data resolved both in time and frequency (Fig. 5A), considering power and phase of the signals separately.”**

In the previous manuscript, further analyses linked the newly identified components to brain regions and characterized them in terms of visual feature complexity (Fig. 6). In the spirit of streamlining, this analysis was moved to the supplementary material (Supplementary Fig. 8).

We also clarified the contribution of the analysis of power and phase in the discussion.

P. 15, ll. 478-482 (Discussion):

> **“Our main contribution lies in identifying four distinct spectro-temporal components, thereby refining our understanding of the spectral basis of recurrent processing. Future work is required to characterize these components in more detail (for a supplementary analysis linking the components to EVC and LOC and clarifying their feature format see Supplementary Fig. 8), and to determine whether they serve distinct functional roles.”**

The explanation why the classifier performance close to the diagonal is negative is a long shot. Isn't it weird to have negative accuracy at all?

We agree that below-chance decoding accuracies are intuitively weird. Yet, negative classifier performance in time-generalization analysis is a common phenomenon (e.g., [12,13]). This is puzzling and requires explanation. The explanation that we put forward in the manuscript is the result of a collaboration with Diego Vidaurre and Mark Woolrich [14], that to our knowledge is also the only account proposed for negative decoding accuracies so far. In the current manuscript, we also acknowledge that the interpretation is tentative, and further research is needed.

P. 5, ll. 172-176 (Results):

> **“How are the negative decoding accuracies in off-diagonal regions of the TGA to be explained? While commonly observed (51,55), the underlying neural dynamics are unknown. One tentative idea is that they might reflect systematic, stimulus-locked shifts in oscillatory phase patterns between the two time points at which the negative decoding occurs (56,57) (for graphical illustration, see Supplementary Fig. 2).”**

Wouldn't it be interesting to analyse the data separately for incorrect and correct trials in the short ISI condition?

We fully agree that analyzing data separately for incorrect and correct trials would be very interesting. However, we collected trial-by-trial responses only for 20% of trials, and only a portion of them were incorrect. Given this limited number of trials, we thus cannot conduct such analysis in a sufficiently powered way. In the discussion, we encourage further research linking neural recurrence to behavior.

P. 13, ll. 426-429 (Discussion):

> **“A limitation of our work is that it characterizes visual representations without directly linking them to object recognition. Further work should relate the observed effects to behavior, for example by contrasting correct and error trials (118,119) or linking neural activity to behavior on a trial-by-trial basis (120,121).”**

Minors:

Intro

39,40: There are no references.

Thanks, we fixed that.

P. 2, ll. 40-41 (Introduction):

> **“Then, recurrent computations begin right after the first influx of feedforward information into the cortex (6–8).”**

56: Would be nice to include here that that "mask" can sometimes be another image

We added this point in the Introduction:

P. 2, ll. 58-59 (Introduction):

> **“[...] naturalistic object stimuli were either backward masked or not using a sequence of synthesized texture images.”**

Results

55-58 Mention the task here?

Acknowledged, we added it in the Introduction.

P.2, ll. 58-61 (Introduction):

> **“Participants performed a 2-alternative forced-choice (2-AFC) task on 20% of EEG trials and identified immediate repetitions in a one-back task on 10% of fMRI trials. We then applied multivariate pattern analysis (41–43) to recover the neural**

representations of object image content across the different masking regimes, over time and space."

Figure 1: could you please add train and test on the axis?

In our time-generalization analysis, we averaged the results across both training-testing directions (i.e., train at time point 1/ test at time point 2, and train at time point 2/ test at time point 1). That is, we made the matrix symmetric and thus the axes are not linked to training/testing anymore. We did this to increase SNR, and because training and testing directions are arbitrary here.

We added a sentence to the caption of Figure 1 to clarify the processing step.

P. 21, ll. 731-733 (Materials and Methods):

> **"As the training and testing directions have no interpretable meaning in this context, we symmetrized the TGM by averaging both train-test directions."**

For the reviewer to ascertain that this processing step did not affect the pattern of results, we provide the result plot that is not averaged across train and test directions in Rebuttal Fig. 9, side by side with the symmetrized matrix:

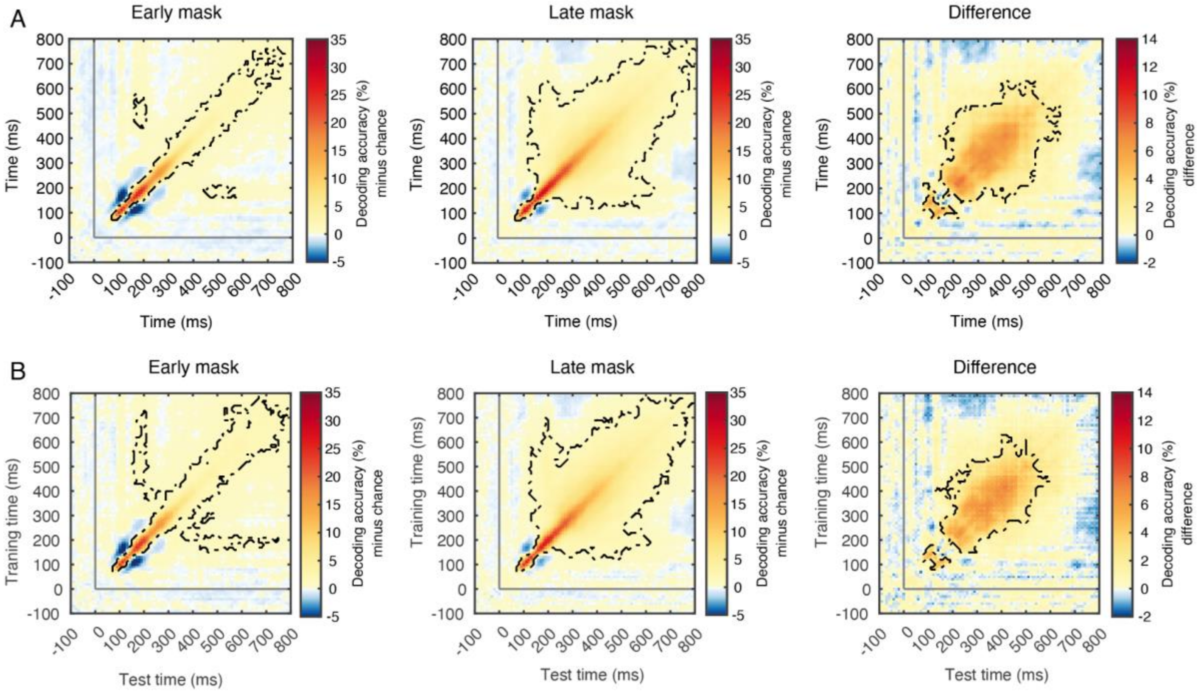

Rebuttal figure 9. Time-generalization results with training and test direction. *The result is organized as presented in Figure 1 E, F, and G for the early mask, late mask conditions, and the differences between the two conditions.*

178: Where is the third statement coming from?

We see that this statement is tentative and would require many assumptions to hold that we cannot test here directly. We thus deleted it, as it does not pertain to our main claims.

Remove the text in original MS P.6, ll. 179-180.

> **"secondly, it contributes specifically to the emergence of persistent representations;"**

186: The greens in C are the average of blue and red in B, isn't it?

Yes, the green bars in C (within condition decoding) are the average of the blue (decoding within late mask condition) and red (decoding within early mask condition) in panel B. We organized these results similarly as in the EEG part. While we have described the rationale in the text for EEG, we now:

(1) refer back to this explanation and clarify it again in the main text.

P. 7, ll. 217-219 (Results):

> **"For this, we compared the within-condition decoding results (Fig. 2C, green bars, corresponds to the average of red and blue bars in Fig. 2B) to the across-conditions results (Fig. 2C, black bars), noting their difference (Fig. 2C, brown bars)."**

(2) add information in the figure captions for Figure 2 as well as the Figure 1.

P. 6, ll.192-195 (Figure 2 caption):

> **"(B) Results of object identity decoding in the early mask condition (red), the late mask condition (blue), and their difference (gray). (C) Results of object identity decoding within (green, corresponds to the average of red and blue in (B)) and across (black) masking conditions and their difference (brown)."**

P. 4, ll. 97-99 (Figure 1 caption):

> **"(D) Results of object identity decoding within (green, corresponds to the average of red and blue in (C)) and across (black) masking conditions and their difference (brown)."**

251: Was it there or not? Not clear

The difference was not statistically significant, although we observed that in the late mask condition the correspondence to the early layers of DNN appeared numerically larger than those to the early mask conditions. We revised the sentence to clarify that this was a nonsignificant numerical trend rather than a statistically significant finding.

P. 9, ll. 261-262 (Results):

> **"Although the difference between masking conditions appeared largest in the early layers, it was not statistically significant."**

262: I don't understand how this proves your hypothesis

The reviewer's comment concerns evidence for the idea that there is a shift in representational format in LOC from lower to higher visual feature complexity through recurrent activity. We see that our writing was unclear, and we have thus changed the text, making our argument more explicit.

P. 9, ll. 267-280 (Results):

> **"[...], in LOC, we observed three key findings that together suggest a shift in representational format from lower to higher visual feature complexity through recurrent activity, indexed by a shift in peak correspondence from early to late DNN layers. First, although both masking conditions showed correspondence with middle to deep DNN layers (layers 3–8; Fig. 3C), the peak correspondence differed: in the early mask condition, it occurred in a middle layer (conv4; [conv4, fc8]), whereas in the late mask condition, it shifted to the deepest layer (fc8; [fc8, fc8]). This shift had an effect size of 0.56 (Hedges' g) and was statistically significant ($p = 0.039$, two-tailed permutation test). Second, comparisons between masking conditions revealed differences in middle to deep layers 4 to 8, with a peak in the deepest layer (i.e., fc8, [fc7, fc8]). Third, in the early mask condition, LOC activity corresponded to early DNN layers (1–2), but this was not observed in the late mask condition. These patterns were also evident when comparing within- and across-conditions decoding results (Supplementary Fig. 5B). Together, they converge to support that recurrent activity in LOC shifts the representational format from lower to higher feature complexity."**

275: How can you deduce it from a feedforward NN?

Figure 3: B,C All layers are significant. D,E here is are some correspondences between the early layers of the DNN and the early/late mask but still you're trying to make conclusions about recurrent connections by using a feedforward NN, isn't it?

Thank you for the questions and comments, echoing your first concern about using feedforward neural networks in this research. We hope our clarification of using the neural nets only as a tool to determine a property of visual representations (their feature complexity) rather than modelling recurrent activity, and the changes affected throughout the manuscript (also pertaining to the specific points here) alleviates the concerns.

Discussion

388: What is an image-specific geometry?

We meant to refer to a representational geometry that is defined by the relationships between images. We see that this term obscures more than it helps, so we cut the sentence from the text.

475: This paragraph could go at the beginning

We restructured the discussion as suggested, bringing the part in Discussion “Backward masking as a tool to dissect recurrent processing” to the beginning.

Now this section is in **P. 12-13, ll. 381-399 (Discussion)**.

Materials and Methods

564: Since participants have high task performance in both masking conditions, it is unclear to what extent masking worked

We agree that the difference in task performance is small. This was a choice we made, striking a balance between effective masking while still allowing for sufficient neural activity to emerge to allow analysis. However, we believe that masking still worked in affecting neural activity, as in spite of arguably small behavioral change, we observed very large change in decoding due to masking (e.g. across compared to within condition in EEG drop by 50% at peak, or fMRI 50% late vs. early mask).

567: Why not showing task performance in the main text?

We added information on task performance in the Results section:

P. 3, ll. 77-79 (Results):

> **“Participants had high task performance in both masking conditions in both EEG and fMRI experiment sessions, and as expected, the task performance was worse for the early mask than the late mask condition.”**

Nitpicking: Helsinki requires pre-registration

We acknowledge that in the newest version (2024), that is after our study was conceptualized and begun, pre-registration was added to the Declaration of Helsinki. We acknowledge the lack of pre-registration.

P. 15, ll. 498-500 (Materials and Methods):

> **“The study was conducted according to the Declaration of Helsinki, with the exception that it was not preregistered, and approved by the local ethics committee of the Freie Universität Berlin.”**

Reference

1. Mohsenzadeh, Y., Qin, S., Cichy, R. M. & Pantazis, D. Ultra-Rapid serial visual presentation reveals dynamics of feedforward and feedback processes in the ventral visual pathway. *eLife* **7**, e36329 (2018).
2. Robinson, A. K., Grootswagers, T. & Carlson, T. A. The influence of image masking on object representations during rapid serial visual presentation. *NeuroImage* **197**, 224–231 (2019).
3. Ritchie, J. B., Tovar, D. A. & Carlson, T. A. Emerging Object Representations in the Visual System Predict Reaction Times for Categorization. *PLOS Comput. Biol.* **11**, e1004316 (2015).
4. Carlson, T. A., Hogendoorn, H., Kanai, R., Mesik, J. & Turret, J. High temporal resolution decoding of object position and category. *J. Vis.* **11**, 9–9 (2011).
5. Cichy, R. M., Pantazis, D. & Oliva, A. Resolving human object recognition in space and time. *Nat. Neurosci.* **17**, 455–462 (2014).
6. Cauchoix, M., Crouzet, S. M., Fize, D. & Serre, T. Fast ventral stream neural activity enables rapid visual categorization. *NeuroImage* **125**, 280–290 (2016).
7. Kar, K., Kubilius, J., Schmidt, K., Issa, E. B. & DiCarlo, J. J. Evidence that recurrent circuits are critical to the ventral stream's execution of core object recognition behavior. *Nat. Neurosci.* **22**, 974–983 (2019).
8. Schmolesky, M. T. *et al.* Signal timing across the macaque visual system. *J. Neurophysiol.* **79**, 3272–3278 (1998).
9. Portilla, J. & Simoncelli, E. P. A Parametric Texture Model Based on Joint Statistics of Complex Wavelet Coefficients. *Int. J. Comput. Vis.* **40**, 49–70 (2000).

10. Prince, J. S. *et al.* Improving the accuracy of single-trial fMRI response estimates using GLMsingle. *eLife* **11**, e77599 (2022).

11. Krizhevsky, A., Sutskever, I. & Hinton, G. E. Imagenet classification with deep convolutional neural networks. *Adv. Neural Inf. Process. Syst.* **25**, (2012).

12. King, J.-R., Pescetelli, N. & Dehaene, S. Brain Mechanisms Underlying the Brief Maintenance of Seen and Unseen Sensory Information. *Neuron* **92**, 1122–1134 (2016).

13. King, J.-R. & Dehaene, S. Characterizing the dynamics of mental representations: the temporal generalization method. *Trends Cogn. Sci.* **18**, 203–210 (2014).

14. Vidaurre, D., Cichy, R. M. & Woolrich, M. W. Dissociable Components of Information Encoding in Human Perception. *Cereb. Cortex* **31**, 5664–5675 (2021).
