# Supplementary material for: Recurrence affects the geometry of visual representations across the ventral visual stream in the human brain
Source: PLoS Biol. 2025 Aug 25;23(8):e3003354. doi: 10.1371/journal.pbio.3003354 (PMC12404645; doi:10.1371/journal.pbio.3003354)
Supplement: Attachment — Submitted filename: R2_Response_to_reviewers.docx [file pbio.3003354.s018.docx]

Note: This file provides detailed point-by-pint response to the editorial requests and reviewers’ comments. Both requests and comments are highlighted in grey, our response is shown in blue, and changes made to the manuscript are indicated in **bold blue**. All corresponding revisions have also been incorporated into the manuscript, where changes appear in red.

**EDITORIAL REQUESTS**

The reviews are appended below. Both reviewers seem to agree that the study has been strengthened in this revision, although reviewer 1 raises a couple of points that we think should be addressed before publication. Reviewer 3 continues to suggest that you remove the CNN data from the paper. I have discussed this request with the Academic Editor, and we think that the way you have used these neural network analyses, as a way to approximate the representations of feature complexity in the visual cortex, is OK. Therefore, we would make the removal of those analyses optional. However, if you do opt to keep this analysis in, we think it may be helpful to explain the use of this model in a bit more depth (for example, we were a bit confused by the rationale for using a feedforward model when you are interested in recurrent dynamics).

Thank you for the evaluation and comments. We decided to leave the CNN analysis in their current form in the manuscript. While our focus indeed is on recurrent dynamics in the brain, we used a feedforward model (AlexNet) for two reasons. First, masking allows us to experimentally isolate feedforward and feedback processes in cortex, so that a separation in a CNN is per se not necessary. Second, to assess how recurrence alters feature complexity, we need a reference model with a well-defined, interpretable feature hierarchy - something feedforward CNNs like AlexNet provide. Recurrent CNNs, by contrast, are less suitable for this purpose due to their temporal complexity and lack of clearly established feature progression across layers. To clarify this rationale, we revised the framing in the Introduction:

**P. 2, ll. 66-72 (Introduction):**

**“Finally, we assessed how recurrence shapes visual representations by comparing neural data to those from a feedforward convolutional neural network (CNN) (48–50). It is important to note that we did not use the CNN to capture brain activity and its recurrent dynamics. The CNN rather serves as a reference model for inferring representational formats in visual cortex, where the model’s well-characterized hierarchical structure allows us to gauge the relative feature complexity within neural representations.”**

**IMPORTANT: In addition to addressing these last reviewer comments, please address the following editorial requests.

1) TITLE: After some discussion within the team, we would like to suggest an edit to the title of your piece. If you agree (and if supported), perhaps you could change the title to

"Recurrence affects the geometry of visual representations across the ventral visual stream in the human brain" or a broader title could be "Elucidating the spatiotemporal dynamics of recurrent processing in visual object recognition"

Thank you for the title suggestions. We have updated the title to:

**“Recurrence affects the geometry of visual representations across the ventral visual stream in the human brain.”**

2) ETHICS STATEMENT: Thank you for including an ethics statement in your methods section. Please update this to include information about the form of consent (written/oral) given for research involving human participants. Please also include an approval number for the protocol approved by the ethics committee at Freie Universität Berlin.

We added this information to the main text.

**p. 16, ll. 509-513 (Materials and Methods):**

**“The study was conducted according to the Declaration of Helsinki, with the exception that it was not preregistered, and approved by the ethics committee of the Department of Education and Psychology at Freie Universität Berlin (protocol approval number: 021.2023). Written informed consent was obtained from all participants prior to data collection.”**

3) DATA: Thank you for providing the underlying code and data for your study as a deposition to OSF. Please add a sentence to each figure legend pointing readers to this dataset. (For example, you can add the sentence "The data underlying this figure can be found at <https://osf.io/U3VG9/>)

Thank you. We have added the sentence **“The data underlying this figure can be found at** [**https://osf.io/U3VG9/**](https://osf.io/U3VG9/)**”** to each figure legend as requested.

**REVIEWERS’ COMMENTS**

Reviewer #1, Ke Jia (note, reviewer 1 has signed this review): I would like to thank the authors for their revised manuscript, which has addressed all of my previous concerns. I have only two minor comments for further consideration:

We thank the reviewer for their thoughtful comments and for the positive evaluation of our revised manuscript.

1 The authors may consider adding more details (e.g., statistical results) regarding behavioral performance in the main text, as this would provide a more comprehensive understanding of the task performance.

Thank you for the suggestion. To keep the main text concise, we have added a summary of task performance to the Results section, with full statistical details provided in the supplementary material.

**P. 3, ll. 81-87 (Results):**

**“Participants had high task performance in both masking conditions across both EEG and fMRI sessions, with high correctness and d-prime scores overall (EEG correctness minus chance (50%): early mask = 29.03 ± 8.67%, late mask = 37.51 ± 6.11%; fMRI d-prime: early mask = 2.01 ± 1.01, late mask = 4.53 ± 2.41), and as expected, the task performance was worse for the early mask than the late mask condition (EEG: p<0.001; fMRI: p<0.001; see Supplementary Table 8 for full behavioral results).”**

2 In the working memory field, emerging evidence suggests that the brain may alter the way it represents memorized information when facing distraction (similar to the design in this manuscript). Given that this study employs a task similar to working memory paradigms, could the observed lower cross-condition decoding (compared to within-condition decoding, which is the main finding) be explained by a change in neural representation during distraction rather than by recurrent/feedback mechanisms?

Thank you for this comment. To clarify, the mask in our study is not used as a distractor. Both the early-mask and late-mask condition include the same masking stimulus; only the timing differs. Thus, difference in cross-condition decoding cannot be attributed to distraction. Future work could investigate how working memory demands or distractors affect representational geometry across regions with varying distraction resistance (e.g., posterior parietal cortex vs. occipitotemporal cortex; Bettencourt and Xu, 2016, Nat Neuroscience) and time. To keep our manuscript streamlined, we decided not to include this in our main text.

Reviewer #3: The authors have addressed most of my concerns well. Still, I think the entire CNN part needs to go, not only spectral analysis. Contrary to the authors claim, CNNs are in no respect validated models of the human brain.

While we appreciate the concerns regarding the CNN analysis, we followed the editors’ assessment and decided to leave the CNN analysis in the manuscript.
