# Supplementary material for: Recurrence affects the geometry of visual representations across the ventral visual stream in the human brain
Source: PLoS Biol. 2025 Aug 25;23(8):e3003354. doi: 10.1371/journal.pbio.3003354 (PMC12404645; doi:10.1371/journal.pbio.3003354)
Supplement: Peer review history

# Editor report: Decision Letter 0

**DOI:** 10.1371/journal.pbio.3003354.r001

**Authors:** Suzanne De Bruijn

30 Oct 2024

Dear Dr Xie,

Thank you for submitting your manuscript entitled "The representational nature of spatio-temporal recurrent processing in visual object recognition" for consideration as a Research Article by PLOS Biology.

Your manuscript has now been evaluated by the PLOS Biology editorial staff as well as by an academic editor with relevant expertise and I am writing to let you know that we would like to send your submission out for external peer review.

However, before we can send your manuscript to reviewers, we need you to complete your submission by providing the metadata that is required for full assessment. To this end, please login to Editorial Manager where you will find the paper in the 'Submissions Needing Revisions' folder on your homepage. Please click 'Revise Submission' from the Action Links and complete all additional questions in the submission questionnaire.

Once your full submission is complete, your paper will undergo a series of checks in preparation for peer review. After your manuscript has passed the checks it will be sent out for review. To provide the metadata for your submission, please Login to Editorial Manager (https://www.editorialmanager.com/pbiology) within two working days, i.e. by Nov 01 2024 11:59PM.

If your manuscript has been previously peer-reviewed at another journal, PLOS Biology is willing to work with those reviews in order to avoid re-starting the process. Submission of the previous reviews is entirely optional and our ability to use them effectively will depend on the willingness of the previous journal to confirm the content of the reports and share the reviewer identities. Please note that we reserve the right to invite additional reviewers if we consider that additional/independent reviewers are needed, although we aim to avoid this as far as possible. In our experience, working with previous reviews does save time.

If you would like us to consider previous reviewer reports, please edit your cover letter to let us know and include the name of the journal where the work was previously considered and the manuscript ID it was given. In addition, please upload a response to the reviews as a 'Prior Peer Review' file type, which should include the reports in full and a point-by-point reply detailing how you have or plan to address the reviewers' concerns.

During the process of completing your manuscript submission, you will be invited to opt-in to posting your pre-review manuscript as a bioRxiv preprint. Visit http://journals.plos.org/plosbiology/s/preprints for full details. If you consent to posting your current manuscript as a preprint, please upload a single Preprint PDF.

Feel free to email us at plosbiology@plos.org if you have any queries relating to your submission.

Kind regards,

Suzanne

Suzanne De Bruijn, PhD

Associate Editor

PLOS Biology

sbruijn@plos.org

# Peer review: Decision Letter 1

**DOI:** 10.1371/journal.pbio.3003354.r002

**Authors:** Christian Schnell, PhD

11 Dec 2024

Dear Dr Xie,

Thank you for your patience while your manuscript "The representational nature of spatio-temporal recurrent processing in visual object recognition" was peer-reviewed at PLOS Biology. It has now been evaluated by the PLOS Biology editors, an Academic Editor with relevant expertise, and by several independent reviewers.

In light of the reviews, which you will find at the end of this email, we would like to invite you to revise the work to thoroughly address the reviewers' reports.

As you will see below, the reviewers you will see that the reviewers are overall interested in the study, but raise a few concerns regarding some specifics of the analyses and the conclusiveness of the work. R2 and R3 also don't think that the CNN model is needed and could be removed. We have discussed this with our Academic Editor and encourage you to remove the CNN model from the manuscript.

Given the extent of revision needed, we cannot make a decision about publication until we have seen the revised manuscript and your response to the reviewers' comments. Your revised manuscript is likely to be sent for further evaluation by all or a subset of the reviewers.

We expect to receive your revised manuscript within 3 months. Please email us (plosbiology@plos.org) if you have any questions or concerns, or would like to request an extension.

At this stage, your manuscript remains formally under active consideration at our journal; please notify us by email if you do not intend to submit a revision so that we may withdraw it.

**IMPORTANT - SUBMITTING YOUR REVISION**

Your revisions should address the specific points made by each reviewer. Please submit the following files along with your revised manuscript:

1. A 'Response to Reviewers' file - this should detail your responses to the editorial requests, present a point-by-point response to all of the reviewers' comments, and indicate the changes made to the manuscript.

*NOTE: In your point-by-point response to the reviewers, please provide the full context of each review. Do not selectively quote paragraphs or sentences to reply to. The entire set of reviewer comments should be present in full and each specific point should be responded to individually, point by point.

You should also cite any additional relevant literature that has been published since the original submission and mention any additional citations in your response.

2. In addition to a clean copy of the manuscript, please also upload a 'track-changes' version of your manuscript that specifies the edits made. This should be uploaded as a "Revised Article with Changes Highlighted" file type.

*Re-submission Checklist*

When you are ready to resubmit your revised manuscript, please refer to this re-submission checklist: https://plos.io/Biology_Checklist

To submit a revised version of your manuscript, please go to https://www.editorialmanager.com/pbiology/ and log in as an Author. Click the link labelled 'Submissions Needing Revision' where you will find your submission record.

Please make sure to read the following important policies and guidelines while preparing your revision:

*Published Peer Review*

Please note while forming your response, if your article is accepted, you may have the opportunity to make the peer review history publicly available. The record will include editor decision letters (with reviews) and your responses to reviewer comments. If eligible, we will contact you to opt in or out. Please see here for more details:

https://blogs.plos.org/plos/2019/05/plos-journals-now-open-for-published-peer-review/

*PLOS Data Policy*

Please note that as a condition of publication PLOS' data policy (http://journals.plos.org/plosbiology/s/data-availability) requires that you make available all data used to draw the conclusions arrived at in your manuscript. If you have not already done so, you must include any data used in your manuscript either in appropriate repositories, within the body of the manuscript, or as supporting information (N.B. this includes any numerical values that were used to generate graphs, histograms etc.). For an example see here: http://www.plosbiology.org/article/info%3Adoi%2F10.1371%2Fjournal.pbio.1001908#s5

*Blot and Gel Data Policy*

We require the original, uncropped and minimally adjusted images supporting all blot and gel results reported in an article's figures or Supporting Information files. We will require these files before a manuscript can be accepted so please prepare them now, if you have not already uploaded them. Please carefully read our guidelines for how to prepare and upload this data: https://journals.plos.org/plosbiology/s/figures#loc-blot-and-gel-reporting-requirements

*Protocols deposition*

To enhance the reproducibility of your results, we recommend that if applicable you deposit your laboratory protocols in protocols.io, where a protocol can be assigned its own identifier (DOI) such that it can be cited independently in the future. Additionally, PLOS ONE offers an option for publishing peer-reviewed Lab Protocol articles, which describe protocols hosted on protocols.io. Read more information on sharing protocols at https://plos.org/protocols?utm_medium=editorial-email&utm_source=authorletters&utm_campaign=protocols

Thank you again for your submission to our journal. We hope that our editorial process has been constructive thus far, and we welcome your feedback at any time. Please don't hesitate to contact us if you have any questions or comments.

Sincerely,

Christian

Christian Schnell, PhD

Senior Editor

PLOS Biology

cschnell@plos.org

------------------------------------

REVIEWS:

Reviewer #1 (Ke Jia): This study by Xie and colleagues combined a backward masking paradigm with fMRI and EEG to investigate the mechanisms of recurrence on visual representations in the human brain. By comparing the early and late masking conditions, they found the spatiotemporal dynamics of changes in representation during recurrence. They further used a deep neural network and revealed four distinct spectro-temporal neural components in EVC and LOC. Overall, the research question is interesting and the authors used a lot of sophisticated analyses. However, there are several confounding factors that have not been controlled very well and I have some major concerns that need to be addressed.

Major concerns:

1. I agree with the authors that backward masking can potentially affect the recurrent or feedback processing. But the difference between early and late masking conditions might also be confounded by other factors. For example, the overall signal to noise ratio may be lower for the early than the late masking conditions, especially between ~100 ms (i.e., the first peak) to ~600 ms (i.e., the onset of the masking in the late condition), as the authors only added noise to the visual systems for the early masking condition during this period. The reduced SNR could also explain other findings presented in the manuscript: a) within vs across conditions decoding using EEG data; b) differences in temporal generalization; c) results of the fMRI data. These confounding factors make the conclusion less convincing.

2. I can not find the display information for the EEG and fMRI experiments. This is important because certain displays may show residual images following the offset of the stimuli. This represents another confounding factor that could explain the difference between early vs. late masking conditions, within vs across conditions decoding analysis and the results of the fMRI data. The authors need to provide more details or conduct control analyses to exclude this possibility.

In addition, I have some minor concerns:

1 In line 76-77, the authors state that 'Because target images and masks were statistically independent by design across trials, classifying objects isolated the neural activity that related to the target from neural activity related to the mask'. Please explain the logic more clearly (i.e., why the authors can 'isolated the neural activity that related to the target')

2 In line 110-116, the authors found a double-peak pattern in the EEG decoding analysis. Is this result in line with previous research? What is the functional role of the double peak?

3 In line 256-265, the authors used the 'shift in the peak correspondence' to investigate the representation format in the fMRI data. In figure 3C, layer 4, layer 5 and layer 8 exhibit high spearman correlation between fMRI RDM and DNN RDM both for the early and the late masking conditions. Is the shift consistent across participants? Please use some statistical methods to support their conclusion.

Reviewer #2: The study by Xie et al. has investigated recurrent processes in the visual system. The authors have been using a simple but informative paradigm where the presentation of visual stimuli is masked 17 ms after the display while brain activity was recorded using MEG and fMRI. The assumption is that the mask abolishes feedback or recurrent processing. By comparing masked vs unmasked presentations, it is possible to uncover when and where the recurrent processing occurs. Furthermore, the findings are linked to CNNs in order to substantiate the argument on processing in early versus late regions. The core finding is that recurrent processing indeed is present at ~100 ms in early visual cortex and then emerges at ~175 and ~300 ms in lateral occipital cortex. This demonstrates that the recurrent activity is persistent rather than transient in lateral occipital cortex. A similar analysis is conducted in the spectral domain. This reveals an early effect peaking at 160 ms in the theta-alpha range and an alpha-beta effect peaking at 540 ms. The linking between brain data and CNNs is based on a representational similarity analysis correlation the respective RDMs. This analysis revealed that recurrence effects were associated with the high-level feature complexity of representations in lateral occipital cortex rather than the early visual cortex.

These are informative findings as they speak to visual processing going beyond the first feedforward visual sweep. It is somewhat a surprise that the recurrent processing emerges already at 100 ms in early visual cortex and then appear in lateral occipital cortex at 175 ms. To me this order suggests that the recurrent processing is not due to a feedback sweep (but the authors might want to speculate on this).

Specific comments

The write-up is somewhat unspecific on recurrent versus feedback processing. I think it would find it helpful to define recurrent processing as processing within one layer or region explained by lateral connectivity. This is different from feedback processing reflected by the flow from higher to lower-level regions. Clarifying these issues would be useful when interpreting the findings of the paper.

If find it surprising that the recurrent processing is present already after 100 ms in early visual regions - and then later in the lateral occipital cortex. This suggests that recurrent activity is not explained by a feedback signal propagating backwards in the ventral stream. Rather the results are in line with local recurrent processing - or the fact that the visual display is changed after 17 ms in the masked condition. The latter concerns could be made explicit in the Discussion when discussing limitations. Also, would an off-response after 17 ms not result in a representational specific activation?

The AlexNet used in the manuscript is purely feedforward. The authors do acknowledge that the aim is not to model brain activity per se, but then it is not so clear to me what we actually learn from the linking to CNNs. CNNs with recurrent properties (e.g CorNet-S) have been developed and might have been more appropriate? This is briefly mentioned and could be more thoroughly elaborated upon (see e.g. Kubilius, J., Schrimpf, M., Nayebi, A., Bear, D., Yamins, D. L., & DiCarlo, J. J. (2018). Cornet: Modeling the neural mechanisms of core object recognition. BioRxiv, 408385.)

The spectral analysis is written as if the effects reflect modulation in oscillatory activity. How do we know that the reported results are not due to evoked activity - which evidently will be reflected in the spectral domain? I would suggestion that the authors do not equate spectral findings with oscillations when introducing and describing the results, but bring up the different interpretations (oscillatory versus evoked activity) in the Discussion section.

The recurrent activities are likely to be highly non-linear. The signals are not simply the sum of feedforward and recurrent activity but rather a complex interaction between both. Please consider this when subtracting the masked versus unmasked conditions and interpreting the results (e.g. Figure 6G (line 424 - 426), 'the spectral basis of recurrent activity in LOC is in power only '; 'recurrent activity is less phase-locked'. Is it for instance possible that the recurrent activity is phase-specific, but it does not affect the feedforward processing in a phase-specific way?

We have concerns on the oscillatory component explanation of the off-diagonal negative accuracy. There is no good reason for the representation of a certain stimulus being time-locked with a half-cycle phase shift. Moreover, there are lots of object pairs, so the phase shifts (if exits) should cover a wide distribution. They cannot always be in anti-phase with each other (if A is anti-phased to B and B is anti-phased to C, then A should be in-phase with C). As such this could be more carefully phrased.

One line 426-428 the authors write: 'This suggests an increase of variability in phase over the course of processing, possibly due to accruing variability as information propagates increasingly back and forth along the visual processing hierarchy.' We are not sure if this conclusion appropriate. The difference on the LOC cannot represent the full recurrent processing. In contrast, the EVC difference shows an increased coefficient of α/β phase component, which could then be interpreted as an increase of phase specificity after the information comes back to EVC.

Line 207 - 209 seems to be a wrong description of the Figure2C? (in regard to bar colors for different conditions)

Line 430: 'Using deep neural networks to model the representations from EEG and fMRI data (45-47), we found that recurrence changes the feature complexity of representations in LOC, but not in EVC.' This seems to bold of a claim as the DNN is a simple model not including recurrents.

Line 527: 'We created a set of image textures to be used for dynamic backward masks. For this, we chose a different subset of 24 object images randomly from the 118-image set and converted the images to textures that conserved the low- and mid-level image statistics of the images without portraying identifiable objects (153).'

This step requires more explanation.

The use of the HRF confusing. At some point a voxel specific is derived from the data and later a canonical HRF is used. Please clarify.

Line 842: ' …. clusters based on temporal or spatial contiguity with a p < 0.005 (i.e., cluster-definition threshold) for most analyses, except for time-frequency decoding matrices, which used a threshold of p < 0.05'

Why were different thresholds used?

The English could be improved.

Somewhat unusual formulations:

'…is incompletely understood due…'

'We observed a qualitatively similar and typical results pattern (48,49) in both masking conditions.' Not so clear that typical refer to

'…conducted in a participant-specific fashion…'

At places many adjacent sentences start with We: ('We …. We…')

Reviewer #3: The interplay between feedforward and recurrent processing is one of the major questions in neuroscience. In the current study, the authors address this question by a combined EEG, fMRI, and CNN study, finding for example that recurrent processing affects feedforward processing already at 100ms. The experiments are carried out in an expert fashion with carefully chosen stimuli. The analysis methods are state of the art and the combination of the various analyses makes this paper an important contribution. The ms is extremely well written and even though the figures are packed they are very clear.

Majors

I do not see what the CNN analysis can tell. Alexnet, nor any other CNN even if recurrent, is a validated model of the human visual system (what human area would correspond to layer 8 in the net?). The authors could as well have used a model for weather forecasting. Btw isn't Alexnet a feedforward net? In any case, I recommend dropping this part entirely.

Why did the authors use an ISI of 600ms for the late mask? I think this is equivalent to a no mask condition, which is missing.

The task does not reflect object recognition but the entire discussion is about it.

I did not get what the analysis about power and phase is about.

The explanation why the classifier performance close to the diagonal is negative is a long shot. Isn't it weird to have negative accuracy at all?

Wouldn't it be interesting to analyse the data separately for incorrect an correct trials in the short ISI condition?

Minors:

Intro

39,40: There are no references.

56: Would be nice to include here that that "mask" can sometimes be another image

Results

55-58 Mention the task here?

Figure 1: could you please add train and test on the axis?

178: Where is the third statement coming from?

186: The greens in C are the average of blue and red in B, isn't it?

251: Was it there or not? Not clear

262: I don't understand how this proves your hypothesis

275: How can you deduce it from a feedforward NN? See above.

Figure 3: B,C All layers are significant. D,E here is are some correspondences between the early layers of the DNN and the early/late mask but still you're trying to make conclusions about recurrent connections by using a feedforward NN, isn't it?

Discussion

388: What is an image-specific geometry?

475: This paragraph could go at the beginning

Materials and Methods

564: Since participants have high task performance in both masking conditions, it is unclear to what extent masking worked

567: Why not showing task performance in the main text?

Nitpicking: Helsinki requires pre-registration

# Author comment: Author response to Decision Letter 2

**DOI:** 10.1371/journal.pbio.3003354.r003

6 Jun 2025

Attachment Submitted filename: Response_to_Reviewers.pdf

# Peer review: Decision Letter 2

**DOI:** 10.1371/journal.pbio.3003354.r004

**Authors:** Lucas Smith

15 Jul 2025

Dear Dr Xie,

Thank you for your patience while we considered your revised manuscript "The representational nature of spatio-temporal recurrent processing in visual object recognition" for consideration as a Research Article at PLOS Biology. Your revised study has now been evaluated by the PLOS Biology editors, the Academic Editor and by two of the original reviewers.

The reviews are appended below. Both reviewers seem to agree that the study has been strengthened in this revision, although reviewer 1 raises a couple of points that we think should be addressed before publication. Reviewer 3 continues to suggest that you remove the CNN data from the paper. I have discussed this request with the Academic Editor, and we think that the way you have used these neural network analyses, as a way to approximate the representations of feature complexity in the visual cortex, is OK. Therefore, we would make the removal of those analyses optional. However, if you do opt to keep this analysis in, we think it may be helpful to explain the use of this model in a bit more depth (for example, we were a bit confused by the the rationale for using a feedforward model when you are interested in recurrent dynamics).

In light of the reviews, we are pleased to offer you the opportunity to address the remaining points from the reviewers in a revision that we anticipate should not take you very long. We will then assess your revised manuscript and your response to the reviewers' comments with our Academic Editor aiming to avoid further rounds of peer-review, although we might need to consult with the reviewers, depending on the nature of the revisions.

**IMPORTANT: In addition to addressing these last reviewer comments, please address the following editorial requests.

1) TITLE: After some discussion within the team, we would like to suggest an edit to the title of your piece. If you agree (and if supported), perhaps you could change the title to

"Recurrence affects the geometry of visual representations across the ventral visual stream in the human brain"

or a broader title could be

"Elucidating the spatiotemporal dynamics of recurrent processing in visual object recognition"

2) ETHICS STATEMENT: Thank you for including an ethics statement in your methods section. Please update this to include information about the form of consent (written/oral) given for research involving human participants. Please also include an approval number for the protocol approved by the ethics committee at Freie Universität Berlin.

3) DATA: Thank you for providing the underlying code and data for your study as a deposition to OSF. Please add a sentence to each figure legend pointing readers to this dataset. (For example, you can add the sentence "The data underlying this figure can be found at https://osf.io/U3VG9/)

In addition to these revisions, you may need to complete some formatting changes, which you will receive in a follow up email. A member of our team will be in touch with a set of requests shortly. If you do not receive a separate email within a few days, please assume that checks have been completed, and no additional changes are required.

We expect to receive your revised manuscript within 1 month. Please email us (plosbiology@plos.org) if you have any questions or concerns, or would like to request an extension.

At this stage, your manuscript remains formally under active consideration at our journal; please notify us by email if you do not intend to submit a revision so that we withdraw the manuscript.

**IMPORTANT - SUBMITTING YOUR REVISION**

Your revisions should address the specific points made by each reviewer. Please submit the following files along with your revised manuscript:

1. A 'Response to Reviewers' file - this should detail your responses to the editorial requests, present a point-by-point response to all of the reviewers' comments, and indicate the changes made to the manuscript.

*NOTE: In your point-by-point response to the reviewers, please provide the full context of each review. Do not selectively quote paragraphs or sentences to reply to. The entire set of reviewer comments should be present in full and each specific point should be responded to individually.

You should also cite any additional relevant literature that has been published since the original submission and mention any additional citations in your response.

2. In addition to a clean copy of the manuscript, please also upload a 'track-changes' version of your manuscript that specifies the edits made. This should be uploaded as a "Revised Article with Changes Highlighted " file type.

*Resubmission Checklist*

When you are ready to resubmit your revised manuscript, please refer to this resubmission checklist: https://plos.io/Biology_Checklist

To submit a revised version of your manuscript, please go to https://www.editorialmanager.com/pbiology/ and log in as an Author. Click the link labelled 'Submissions Needing Revision' where you will find your submission record.

Please make sure to read the following important policies and guidelines while preparing your revision:

*Published Peer Review*

Please note while forming your response, if your article is accepted, you may have the opportunity to make the peer review history publicly available. The record will include editor decision letters (with reviews) and your responses to reviewer comments. If eligible, we will contact you to opt in or out. Please see here for more details:

https://blogs.plos.org/plos/2019/05/plos-journals-now-open-for-published-peer-review/

*PLOS Data Policy*

Please note that as a condition of publication PLOS' data policy (http://journals.plos.org/plosbiology/s/data-availability) requires that you make available all data used to draw the conclusions arrived at in your manuscript. If you have not already done so, you must include any data used in your manuscript either in appropriate repositories, within the body of the manuscript, or as supporting information (N.B. this includes any numerical values that were used to generate graphs, histograms etc.). For an example see here: http://www.plosbiology.org/article/info%3Adoi%2F10.1371%2Fjournal.pbio.1001908#s5

*Blot and Gel Data Policy*

We require the original, uncropped and minimally adjusted images supporting all blot and gel results reported in an article's figures or Supporting Information files. We will require these files before a manuscript can be accepted so please prepare them now, if you have not already uploaded them. Please carefully read our guidelines for how to prepare and upload this data: https://journals.plos.org/plosbiology/s/figures#loc-blot-and-gel-reporting-requirements

*Protocols deposition*

To enhance the reproducibility of your results, we recommend that if applicable you deposit your laboratory protocols in protocols.io, where a protocol can be assigned its own identifier (DOI) such that it can be cited independently in the future. Additionally, PLOS ONE offers an option for publishing peer-reviewed Lab Protocol articles, which describe protocols hosted on protocols.io. Read more information on sharing protocols at https://plos.org/protocols?utm_medium=editorial-email&utm_source=authorletters&utm_campaign=protocols

Thank you again for your submission to our journal. We hope that our editorial process has been constructive thus far, and we welcome your feedback at any time. Please don't hesitate to contact us if you have any questions or comments.

Sincerely,

Luke

Lucas Smith, Ph.D.

Senior Editor

PLOS Biology

lsmith@plos.org

----------------------------------------------------------------

REVIEWS:

Reviewer #1, Ke Jia (note, reviewer 1 has signed this review): I would like to thank the authors for their revised manuscript, which has addressed all of my previous concerns. I have only two minor comments for further consideration:

1 The authors may consider adding more details (e.g., statistical results) regarding behavioral performance in the main text, as this would provide a more comprehensive understanding of the task performance.

2 In the working memory field, emerging evidence suggests that the brain may alter the way it represents memorized information when facing distraction (similar to the design in this manuscript). Given that this study employs a task similar to working memory paradigms, could the observed lower cross-condition decoding (compared to within-condition decoding, which is the main finding) be explained by a change in neural representation during distraction rather than by recurrent/feedback mechanisms?

Reviewer #3: The authors have addressed most of my concerns well. Still, I think the entire CNN part needs to go, not only spectral analysis. Contrary to the authors claim, CNNs are in no respect validated models of the human brain.

# Author comment: Author response to Decision Letter 3

**DOI:** 10.1371/journal.pbio.3003354.r005

5 Aug 2025

Attachment Submitted filename: R2_Response_to_reviewers.docx

# Editor report: Decision Letter 3

**DOI:** 10.1371/journal.pbio.3003354.r006

**Authors:** Lucas Smith

7 Aug 2025

Dear Dr Xie,

Thank you for the submission of your revised Research Article "Recurrence affects the geometry of visual representations across the ventral visual stream in the human brain" for publication in PLOS Biology and thank you for addressing the last reviewer and editorial requests in this revision. On behalf of my colleagues and the Academic Editor, Christopher Pack, I am pleased to say that we can in principle accept your manuscript for publication, provided you address any remaining formatting and reporting issues. These will be detailed in an email you should receive within 2-3 business days from our colleagues in the journal operations team; no action is required from you until then. Please note that we will not be able to formally accept your manuscript and schedule it for publication until you have completed any requested changes.

Please take a minute to log into Editorial Manager at http://www.editorialmanager.com/pbiology/, click the "Update My Information" link at the top of the page, and update your user information to ensure an efficient production process.

PRESS

We frequently collaborate with press offices. If your institution or institutions have a press office, please notify them about your upcoming paper at this point, to enable them to help maximize its impact. If the press office is planning to promote your findings, we would be grateful if they could coordinate with biologypress@plos.org. If you have previously opted in to the early version process, we ask that you notify us immediately of any press plans so that we may opt out on your behalf.

We also ask that you take this opportunity to read our Embargo Policy regarding the discussion, promotion and media coverage of work that is yet to be published by PLOS. As your manuscript is not yet published, it is bound by the conditions of our Embargo Policy. Please be aware that this policy is in place both to ensure that any press coverage of your article is fully substantiated and to provide a direct link between such coverage and the published work. For full details of our Embargo Policy, please visit http://www.plos.org/about/media-inquiries/embargo-policy/.

Thank you again for choosing PLOS Biology for publication and supporting Open Access publishing. We look forward to publishing your study. 

Sincerely, 

Luke

Lucas Smith, Ph.D.

Senior Editor

PLOS Biology

lsmith@plos.org
